# Guidelines for DNA recombination and repair studies: Cellular assays of DNA repair pathways

**DOI:** 10.15698/mic2019.01.664

**Published:** 2019-01-07

**Authors:** Hannah L. Klein, Giedrė Bačinskaja, Jun Che, Anais Cheblal, Rajula Elango, Anastasiya Epshtein, Devon M. Fitzgerald, Belén Gómez-González, Sharik R. Khan, Sandeep Kumar, Bryan A. Leland, Léa Marie, Qian Mei, Judith Miné-Hattab, Alicja Piotrowska, Erica J. Polleys, Christopher D. Putnam, Elina A. Radchenko, Anissia Ait Saada, Cynthia J. Sakofsky, Eun Yong Shim, Mathew Stracy, Jun Xia, Zhenxin Yan, Yi Yin, Andrés Aguilera, Juan Lucas Argueso, Catherine H. Freudenreich, Susan M. Gasser, Dmitry A. Gordenin, James E. Haber, Grzegorz Ira, Sue Jinks-Robertson, Megan C. King, Richard D. Kolodner, Andrei Kuzminov, Sarah AE Lambert, Sang Eun Lee, Kyle M. Miller, Sergei M. Mirkin, Thomas D. Petes, Susan M. Rosenberg, Rodney Rothstein, Lorraine S. Symington, Pawel Zawadzki, Nayun Kim, Michael Lisby, Anna Malkova

**Affiliations:** 1 Department of Biochemistry and Molecular Pharmacology, New York University School of Medicine, New York, NY, USA.; 2 Department of Biology, University of Copenhagen, DK-2200 Copenhagen N, Denmark.; 3 Department of Radiation Oncology, University of Texas Health Science Center at San Antonio, 7703 Floyd Curl Drive, San Antonio, TX, USA.; 4 Friedrich Miescher Institute for Biomedical Research (FMI), 4058 Basel, Switzerland.; 5 Department of Biology, University of Iowa, Iowa City, IA, USA.; 6 Dan L Duncan Comprehensive Cancer Center, Baylor College of Medicine, Houston, TX, USA.; 7 Department of Molecular and Human Genetics, Baylor College of Medicine, Houston, TX, USA.; 8 Department of Biochemistry and Molecular Biology, Baylor College of Medicine, Houston, TX, USA.; 9 Department of Molecular Virology and Microbiology, Baylor College of Medicine, Houston, TX, USA.; 10 Centro Andaluz de BIología Molecular y Medicina Regenerativa-CABIMER, Universidad de Sevilla, Seville, Spain.; 11 Department of Microbiology, University of Illinois at Urbana-Champaign, Urbana, IL, USA.; 12 Yale School of Medicine, New Haven, CT, USA.; 13 Department of Microbiology and Immunology, Columbia University Medical Center, New York, NY, USA.; 14 Systems, Synthetic and Physical Biology Graduate Program, Rice University, Houston, TX, USA.; 15 Department of Molecular Biosciences, University of Texas at Austin, Austin, TX, USA.; 16 Institut Curie, PSL Research University, CNRS, UMR3664, F-75005 Paris, France.; 17 Sorbonne Université, Institut Curie, CNRS, UMR3664, F-75005 Paris, France.; 18 NanoBioMedical Centre, Faculty of Physics, Adam Mickiewicz University, Umultowska 85, 61-614 Poznan, Poland.; 19 Department of Biology, Tufts University, Medford, MA USA.; 20 Ludwig Institute for Cancer Research, University of California School of Medicine, San Diego, La Jolla, CA, USA.; 21 Department of Medicine, University of California School of Medicine, San Diego, La Jolla, CA, USA.; 22 Institut Curie, PSL Research University, CNRS, UMR3348 F-91405, Orsay, France.; 23 University Paris Sud, Paris-Saclay University, CNRS, UMR3348, F-91405, Orsay, France.; 24 Genome Integrity and Structural Biology Laboratory, National Institute of Environmental Health Sciences, Durham, NC, USA.; 25 Department of Biochemistry, University of Oxford, South Parks Road, Oxford, OX1 3QU, UK.; 26 Department of Molecular Genetics and Microbiology and University Program in Genetics and Genomics, Duke University Medical Center, Durham, NC USA.; 27 Department of Environmental and Radiological Health Sciences, Colorado State University, Fort Collins, CO, USA.; 28 Program in Genetics, Tufts University, Boston, MA, USA.; 29 Department of Biology and Rosenstiel Basic Medical Sciences Research Center Brandeis University, Waltham, MA, USA.; 30 Department of Molecular Genetics and Microbiology, Duke University Medical Center, Durham, NC USA.; 31 Department of Cellular and Molecular Medicine, University of California School of Medicine, San Diego, La Jolla, CA, USA.; 32 Moores-UCSD Cancer Center, University of California School of Medicine, San Diego, La Jolla, CA, USA.; 33 Institute of Genomic Medicine, University of California School of Medicine, San Diego, La Jolla, CA, USA.; 34 Department of Genetics & Development, Columbia University Irving Medical Center, New York, NY, USA.; 35 Department of Microbiology and Molecular Genetics, The University of Texas Health Science Center at Houston, Houston, TX, USA.

**Keywords:** chromatin dynamics, chromosome rearrangements, crossovers, DNA breaks, DNA repair centers, DNA resection, DSBs, gene amplification, gene conversion, genome instability, gross chromosome rearrangements, fluorescent proteins, Holliday junctions, homologous recombination, mitotic recombination, mutagenesis, pulsed field gel electrophoresis, R-loops, single-particle tracking, replication fork stalling, sister repetitive sequences, sister chromatid recombination, site-specific chromosome breaks, toxic recombination intermediates, yeast artificial chromosome

## Abstract

Understanding the plasticity of genomes has been greatly aided by assays for recombination, repair and mutagenesis. These assays have been developed in microbial systems that provide the advantages of genetic and molecular reporters that can readily be manipulated. Cellular assays comprise genetic, molecular, and cytological reporters. The assays are powerful tools but each comes with its particular advantages and limitations. Here the most commonly used assays are reviewed, discussed, and presented as the guidelines for future studies.

## INTRODUCTION

Genomes are subject to spontaneous and induced DNA damage throughout the life cycle of the host organism. DNA damage can be repaired to the original DNA sequence without a change or altered through mutagenesis and recombination events. To detect changes, genetic and molecular assays have been developed in model systems. As mutagenesis and recombination repair are rare events, reporter assays have been developed that allow for the selection of rare events. Cytological assays provide information as to real time repair events, with detection of the spatio-temporal organization of DNA and protein complexes during the repair process.

The genetic assays are based on either nutritional selection or drug/compound resistance and may be combined with physical assays of the interacting DNA molecules. Each assay is tailored to the specific type of repair event under study. Thus, it is important to bear in mind that not all repair events are captured, and that the assay is a read-out seen as a change in phenotype that may arise from more than one type of repair process. Nonetheless, this approach has proved to be very informative for the characterization of proteins involved and even specific mutations within these proteins that incapacitate various activities such as a nuclease or a DNA helicase or a protein interaction. The reporter assays have also been useful in parsing out the response to a particular DNA damaging agent, to determine how the DNA is damaged and repaired. These assays have also been applied to the repair pathway elucidation through the use of reporters in multiple mutant background strains. When genetic assays are combined with molecular analyses of the repair products, the combination becomes a powerful tool for the understanding of the specific repair pathway. This is further augmented with cytological studies.

Molecular assays for DNA damage and repair require that most or all of the cells have undergone the same event. Such assays can be used to examine the consequences of damage repair, such as chromosome rearrangements. Molecular assays can also be used to examine the processing and repair of induced damage, most frequently a double strand break. In these types of assays a break is induced at a specific site by placing an enzyme recognition site at a specific chromosome locus, surrounded by DNA sequences that are known and can be readily assayed by known restriction enzyme recognition sites. These assays can detect transient intermediates if sufficiently abundant, toxic intermediates and final products. Repair can be monitored in real time by PCR analysis, Southern blot studies, or pulsed field gel electrophoresis to detect large DNA molecules.

Cytological assays allow for detection of DNA lesions, DNA repair intermediates and DNA repair proteins at the single-cell level thereby providing an opportunity to deconstruct the order of events during the DNA damage response in living cells. The single cell approach can reveal cell-to-cell heterogeneity in the progression of repair processes and uncover rare events that are often lost in population-based assays. Cytological assays have also revealed global changes in nuclear organization of chromatin during DNA repair. By use of digital image analysis software, biochemical information such as diffusion rates, binding constants, and stoichiometry of repair complexes can be deduced for DNA repair proteins in their natural environment.

Here, these three areas for the study of DNA recombination and repair are presented. Although there is a similarity in some of the assays, each has been tailored to study specific repair processes. Some of the assays have been portable into mammalian cells with modification of the reporter readouts. They have proved to be powerful tools for the study of genome instability in cancer cells with defects in DNA HR genes, cell cycle checkpoint genes and non-HR repair processes. Mutations in all of these types of genes have been associated cancers and other human diseases. These guidelines should prove useful for the wider application of the current protocols and the development of additional assays. Individual author contributions and contact information are available in Supplementary Table 1.

## GENETIC ASSAYS TO DETECT DNA RECOMBINATION AND REPAIR

### Methods to detect mutagenesis

#### Forward mutation assays

Mutations negatively impacting the normal function of the gene product can be detected, provided that a practical method of counter-selection is available. In such “Forward mutation assays”, the rates of mutations can be a general indicator of the repair efficiency of a given genetic background as well as the mutagenic effect of a particular treatment. In *Saccharomyces cerevisiae*, mutations occurring anywhere in the *CAN1* or *URA3* genes are detected by the resistance to the drug canavanine (Can) or 5-fluoroorotic acids (5-FOA), respectively. *CAN1*, which encodes an arginine permease spanning the plasma membrane, has been a useful tool as a counter-selectable marker of mutagenesis since the import of the amino acid arginine as well as its toxic analog canavanine is disrupted when *CAN1* gene is mutated, leading to Can^R^ phenotype. Forward mutations in *URA3* result in resistance to the drug 5-FOA, which is not toxic unless converted to 5-fluorouracil (5-FU) by the *URA3*-encoded orotidine-5-phosphate decarboxylase. Genetic or chemical disruption in the DNA repair pathways or the addition of the DNA damaging agents will manifest as the elevation of the rate of Can^R^ or 5-FOA^R^ mutations. The identity of mutations analyzed by Sanger sequencing of *CAN1* ORF in the Can^R^ mutants has led to interesting findings regarding the mutagenesis mechanism under particular conditions. For example, in cells treated with the alkylating agent methyl methanesulfonate (MMS), the mutations at the *CAN1* gene largely comprised of base substitutions, most prominently G:C to T:A transversions [1]. Another experiment showed that the *CAN1* mutation spectra shifted from comprising mostly of base-substitutions (>80%) to mostly of 2-5 bp deletions (>50%) when the transcription of the gene was elevated [2]. The deletion of topoisomerase I-encoding *TOP1* gene led to the disappearance of the 2-5 bp deletions, demonstrating how the forward mutation assay combined with the subsequent survey of the mutation spectrum could yield critical details about the mechanism underlying a particular type of mutations. In a *URA3* forward mutation assay in yeast cells expressing mutant polymerase δ or ε, specific hot spots of mutation were identified that serve as the mark of the error introduced by the respective mutant DNA polymerases [3, 4]. By incorporating the *URA3* gene adjacent to the early firing replication origin ARS306 in two different orientations, the sequences of the 5-FOA^R^ mutations were used to determine whether polymerase ε or polymerase δ functions mainly during leading- or lagging-strand synthesis. Despite their great utility as the preliminary indicator of the mutagenicity, however, the analyses into the types of mutations occurring like those described above is generally hampered by the relatively large size of the *CAN1* (1770 nt) or *URA3* (801 nt).

BOX 1: **GENETIC ASSAYS TO DETECT DNA RECOMBINATION AND REPAIR**

**Forward mutation assays |** Use of genes where loss of function recessive mutations can be selected through resistance to medium compound**.**

**Reversion mutation assays |** Restoration of function or prototrophy through reversion of a specific mutation. These assays may detect base pair changes or frameshift mutations.

**Sister chromatid recombination |** Assays to detect double strand break repair through exchange between sister chromatids.

**Direct repeat assays |** Measures gene conversion, single strand annealing and crossover recombination using heteroallelic repeats to detect rare events. Events may be spontaneous or induced by a double strand break at a cut site introduced into one of the repeats.

**Recombination in diploid cells |** Use of color assays for red/white colony sectoring to detect gene conversion with or without an associated crossover. This assay is often used for loss of heterozygosity (LOH) events.

**Gross chromosomal rearrangements |** These assays detect translocations, deletions, amplifications and chromosome fusions, all termed gross chromosomal rearrangements. The basic design of the assays use multiple counterselectable markers embedded in the non-essential terminal regions of chromosome arms.

**Repeat expansions and genome instability |** Assays to monitor repeat expansion through interference of intron function in a counterselectable gene. This assay can be adapted to many repetitive DNA sequences to determine instability.

**Yeast artificial chromosomes and DNA sequence fragility |** Insertion of simple repeat tracts in an artificial chromosome with counterselectable markers is used to monitor breakage and aberrant repair of these sequences.

**Chromosome rearrangements associated with gene amplification |** Use of genes that result in resistance/tolerance to cytotoxic compounds in a dosage-dependent fashion. This type of assay detects copy number variation (CNV) and can be used to detect chromosome rearrangements associated with gene amplification.

#### Reversion mutation assays

A mutation type of particular interest at a defined location can be detected by purposely designed reversion mutation assays. In mutagenesis assays where an in-frame stop codon is inserted into the open reading frame (ORF) of a selective marker gene, a range of base-substitutions negating the stop codon can be detected by the phenotypic reversion. *LYS2* gene encodes an alpha aminoadipate reductase, essential for the lysine biosynthesis. Mutations at the TAA stop codon inserted into the *LYS2* ORF- other than TAA to TGA or TAG – results in the selectable Lys+ phenotype. While the rate of Lys+ mutation at the *lys2-TAA* allele can be a measure of the DNA damage and/or repair efficiency, the types of mutations can provide further information into how these mutations occur. Although TAA to GAA or TCA mutations are prevalent when the base excision repair pathway is disrupted, these mutations are drastically reduced upon the deletion of translesion polymerase-encoding genes *REV1* or *REV3* [5]. Further, the study showed that the dCMP-transferase activity of Rev1 is critical in the T:A to G:C transversion mutations. Another example of a reversion assay is the *trp5* assay. A screen for the trp- auxotroph identified the Glu-50 residue of the Trp5 protein to be essential for its function in tryptophan biosynthesis [6]. Starting with the *trp5* alleles with NAA or GNA at the codon 50 (nt position 148, 149, and 150), the rate of true reversion mutations, restoring the GAA codon, are identified by selecting for Trp+ cells. Starting with six *trp5* mutant strains (A148, C148, T148, C149, G149, T149), this assay allows the comparative analysis of the rate of six different base substitutions (e.g. A148G (AAA to GAA) and G149A (GGA to GAA). Using this assay, the mutation signatures of UV and 5-AZ were identified as G to A and C to G, respectively.

#### Versatile, frameshift reversion assays to detect frameshift mutations

**Figure 1:**
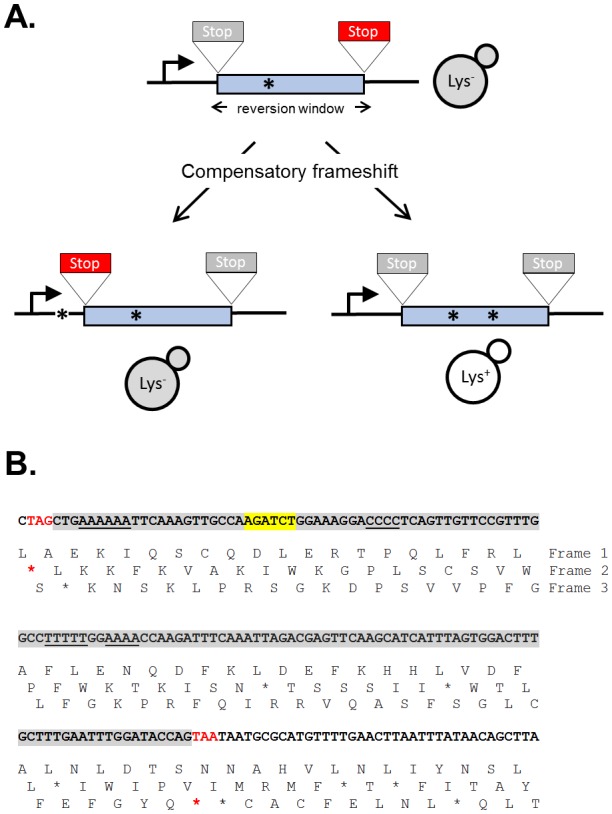
FIGURE 1: *LYS2*-based frameshift reversion assays. **(A)** Cartoon illustrating how stop codons in alternative reading frames define a reversion window (blue). The horizontal arrow indicates the direction of transcription and asterisks correspond to frameshift mutations. A stop codon encountered during translation is red; a gray stop codon is in a different reading frame and does not affect translation. **(B)** Sequence of the region of the *LYS2* ORF that contains the *lys2ΔBgl* reversion window (gray), which is delimited by the boxed stop codons. Mononucleotide runs >3N are underlined and the *Bgl*II site is highlighted yellow.

The sequence of a gene contains three alternative reading frames that reflect the triplet structure of codons. Only one of these frames encodes the functional product and is designated as the ORF; the other two frames specify different amino acids and are punctuated by frequent stop codons. A frameshift mutation is defined as a net addition or deletion of base pairs (bp) in a non-multiple of three (+1 and -1 frameshifts, respectively), which “shifts” the ORF into one of the two alternative reading frames. After the shift, a translating ribosome adds incorrect amino acids to the growing peptide until the first stop codon is encountered and this almost invariably gives rise to a non-functional product. Reversion of a frameshift mutation can occur by reversal of the original mutation or through acquisition of a compensatory frameshift mutation of net opposite sign. The compensatory frameshift is constrained to occur in a theoretical window defined by the most proximal stop codons in the alternative reading frames (**Figure 1A**). A compensatory mutation downstream (promoter distal) of a frameshift mutation, for example, must occur before the first stop codon is encountered in the shifted reading frame. Upstream of the frameshift mutation, the compensatory change must not occur so far upstream that it leads to the encounter of a stop codon in the other, alternative frame. The region between the original and compensatory frameshifts specifies incorrect amino acids that may inactivate the encoded protein, and this can further restrict the theoretical reversion window.

The yeast *LYS2* gene is essential for lysine production, and mutants fail to grow on minimal medium lacking lysine. Selection for growth of mutants allows the ready identification of Lys^+^ revertants, and forward mutations in *LYS2* can additionally be identified by their ability to grow in the presence of the toxic compound α-aminoadipate [7]. The *LYS2* ORF is large by yeast standards (∼4.2 kb) and its utility in frameshift reversion assays was discovered following the filling in of a unique *Bgl*II restriction site located ∼390 nt from the start codon [8]. This adds 4 bp to the sequence and creates a net +1 frameshift mutation. Whereas most frameshift mutations fail to revert in the absence of an added mutagen, the resulting *lys2ΔBgl* allele reverts spontaneously at a rate of ∼2 x 10^-9^. The theoretical reversion window where a compensatory, net -1 frameshift can occur is ∼150 bp and is highlighted gray in **Figure 1B**. The *Bgl*II site is indicated in yellow and the ORF is reading frame 1; the addition of 4 bp shifts translation to reading frame 3. A compensatory frameshift downstream of the filled in *Bgl*II site must occur before the first stop codon (boxed) in reading frame 3 is encountered and this delimits the distal end of the reversion window. In the other alternative reading frame (frame 2), an upstream compensatory frameshift will terminate translation if it occurs before the first stop codon upstream of the engineered frameshift. In early studies, compensatory, net -1 frameshift mutations were scattered throughout the window, suggesting tolerance to most if not all amino acid substitutions in the corresponding region of the Lys2 protein. After characterization of *lys2ΔBgl* revertants, an analogous -1 frameshift allele (*lys2ΔA746*) was constructed in the same region, allowing the isolation and characterization of net +1 compensatory frameshifts [9]. More recently, it was found that large deletions that remove the frameshift mutation and generate an in-frame fusion protein produce a functional Lys2 protein, revealing that the first ∼700 bp of the ORF are functionally dispensable (J.E. Cho and S. Jinks-Robertson, unpublished). In addition, the insertion of at least 1 kb of in-frame exogenous sequence into the reversion window is tolerated (Y.F. Hum and S. Jinks-Robertson, unpublished).

The *lys2* frameshift-reversion assays have been used to study a variety of DNA-metabolic processes. Studies typically involve the measurement of reversion rates coupled with sequencing of the reversion window to determine mutation patterns and rates of specific of frameshift types. Early studies with the *lys2ΔBgl* and *lys2ΔA746* alleles revealed, for example, that the most common mutations were deletions and additions of a single bp, respectively. Most occurred due to DNA polymerase slippage in mononucleotide runs >3N (underlined in **Figure 1B**), and these “hotspots” were further amplified upon disruption of the post -replicative mismatch repair machinery [8, 9]. To fur-ther examine properties of DNA polymerase slippage and subsequent mismatch repair, 10N runs that were either out-of-frame or in-frame were inserted into the reversion window, with the latter being used to identify forward mutations that alter the length of the engineered run [10, 11]. In addition to examining frameshift mutagenesis that occurs in the context of replicative DNA synthesis, the *lys2ΔA746* assay has been used to study the genetic regulation of DNA damage bypass by Pol ζ, an error-prone translesion synthesis DNA polymerase [12]. DNA damage that persists in the absence of the nucleotide excision repair pathway, for example, is associated with appearance of distinctive mutation hotspots in which the selected +1 frameshift mutation is associated with one or more base substitutions. These novel events require Pol ζ activity [13]. Given the bias for frameshift mutations to occur in mononucleotide runs, these runs were eliminated in order to study other potential types of frameshifts. *De novo* duplications not detected previously became prominent and were shown to require the non-homologous end-joining pathway that is used to repair double-strand breaks [14]. Finally, the *lys2* frameshift reversion assays have been the platform for assessing the effects of high levels of transcription on stability of the underlying DNA template [15]. This has enabled the study of templated mutations that occur at quasi-palindromes [16] and has been particularly useful for studying a 2-bp deletion signature that reflects Top1 activity [17]. Finally, the functional constraints on sequence within the reversion window has allowed for the introduction of out-of-frames cleavage sites for mega-endonucleases and the study of their subsequent repair by error-prone nonhomologous end-joining (S. Shaltz and S. Jinks-Robertson, unpublished) or homologous recombination [18].

The dispensability of the amino terminus of the *LYS2* gene has permitted the construction/insertion of defined types of frameshift alleles and the study of the diverse mechanisms that revert them. Although small changes are limited to the reversion window, this window could be expanded to at least a kb by inserting a synthetic fragment that lacks stop codons in all three reading frames. In addition, the ability of the Lys2 protein to function without its amino terminus allows large deletions that create functional fusion proteins to be identified. Although the description here has been limited to the *LYS2* gene, the same principles can be applied to any gene with a similarly dispensable region or to N-terminal fusion proteins.

### Methods to detect mitotic recombination

DNA breaks are among the most harmful lesions; they block transcription and replication causing cell lethality unless properly repaired. Double strand breaks (DSBs) can be repaired by different mechanisms, from non-homologous (NHEJ) and Micro-homology-mediated End Joining (MMEJ) to homologous recombination (HR). HR is the most prominent error-free mechanism of DSB repair. It relies on the interchange of information between two homologous DNA sequences and on the copying of information from a homologous template to seal the break [19, 20]. DSBs occurring in the S-G2 period of the cell cycle are preferentially repaired by HR, when the sister chromatid can be used as a template in an error-free manner to maintain the stability of the genome [21–23].

Homologous recombination has been studied both in mitosis and meiosis since the beginning of the century in Drosophila, bacteria, yeast, filamentous fungi and in superior organisms using genetic assays. The detection of mitotic recombination originally relied on crosses between strains carrying heterozygous markers that allows to distinguish phenotypically the recombinant products from the parental configuration. Thus, mitotic recombination was studied between homologous chromosomes in eukaryotic diploids or prokaryotic merodiploids. The capacity to artificially engineer genomes allowed to generate intrachromosomal systems for the detection of HR that fostered the studies on mitotic recombination [24, 25, 26]. All systems for detection of recombination developed in the last three decades have proved to be tremendously effective to decipher and expand our knowledge on the different mechanisms of HR, including Single-Strand Annealing, Synthesis-Dependent Strand Annealing, Gene Conversion, Crossovers, etc. [27, 28]. Many different assays have been developed for the genetic detection of recombination. Along the years, a number of them have been developed, both in plasmids and integrated in chromosomes for the analysis of different mitotic recombination events and mechanisms. A detailed description of them together with the methodology of use has been published [29].

#### Method to detect Sister Chromatid Recombination

**Figure 2:**
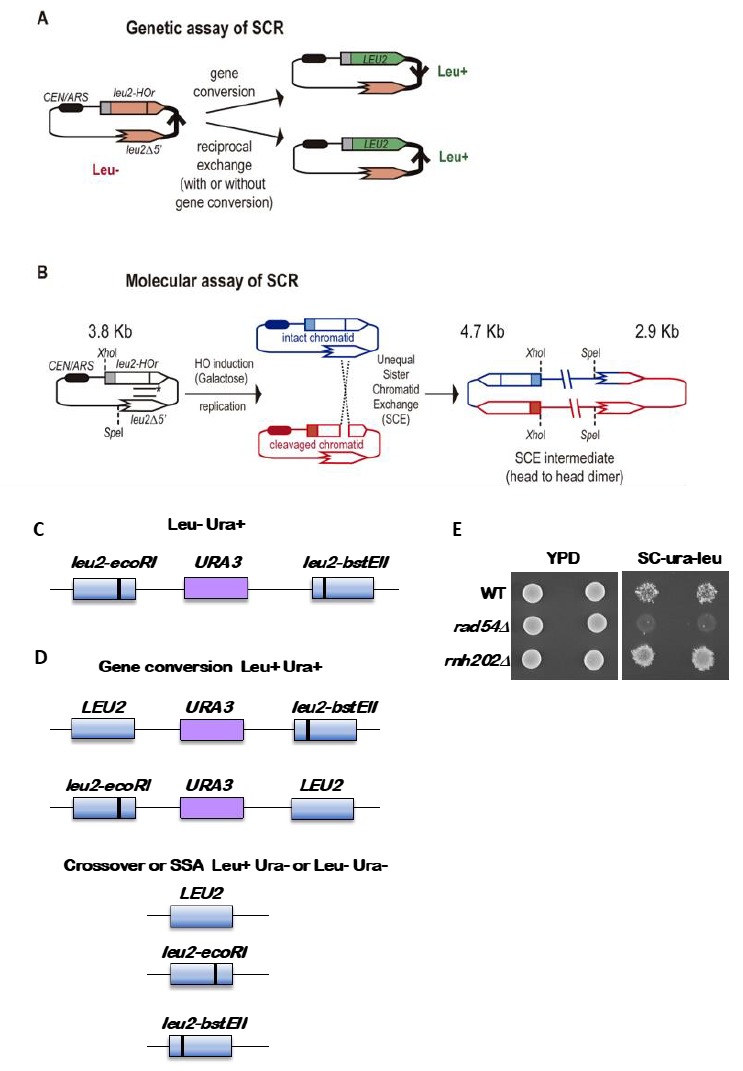
FIGURE 2: Assays to detect chromatid recombination. **(A)** Genetic assay of sister chromatid recombination (SCR). **(B)** Molecular assay of sister chromatid recombination (SCR). **(C)** Cartoon of the direct repeated *leu2* genes with the *URA3* gene inserted in between, with the black vertical line indicating the *leu2* mutations. The entire reporter is integrated at the *LEU2* locus and strains with the reporter have a Leu- Ura+ phenotype. **(D)** Recombination outcomes. Gene conversions are Leu+ Ura+ and retain the duplication, with one copy of the *LEU2* gene now being wild type. Loss of the *URA3* gene by a crossover or single strand annealing (SSA) yields Ura- segregants that can be Leu+ or Leu-. **(E)** Examples of Leu+ Ura+ papillae from three strains, wild type, *rad54,* which has very low gene conversion recombination rate, and *rnh202*, which has an increased gene conversion rate.

Provided that spontaneous breaks arise commonly during and after replication, and that the sister chromatid is the preferred template for HR, Sister Chromatid Recombination (SCR) can be considered the major mechanism for HR repair. It is, therefore, important to decipher whether SCR uses the same HR factors and mechanisms than recombination between homologous chromosomes as well as to determine which are the factors that specifically condition SCR. The first assay to study SCR in the yeast *S. cerevisiae* was a chromosomal genetic system based on two truncated repeats of the *HIS3* gene in a direct orientation [30]. Recombination with the other repeat on the sister chromatid (unequal Sister-Chromatid Exchange, SCE) leads to the triplication of the *HIS3* allele and allows the genetic detection of His+ recombinants [30]. This assay is not only limited to the analysis of unequal SCE but also does not allow physical detection of recombination intermediates and therefore the analysis of the kinetics of the reaction is not possible. To bypass this limitation, the two plasmid recombination systems (*pL2-HOr* and *pTINV*) that enable the study of SCR by physical methods were developed [23, 31]. In both of them, a 24-bp mini-HO site leads preferentially to nicks in the DNA after activation of the HO endonuclease, which is expressed from a *GAL1* promoter. HO-induced nicks are then converted to DSBs by replication [31]. The *pL2-HOr* system is based on a mutated copy of the *LEU2* gene containing the mini-HO site [23]. Replication-born DSBs can be repaired via SCR with the equal repeat in the sister-chromatid (equal SCE) leading to DNA intermediates that can be detected physically by Southern-blot [23]. The *pTINV* system, by contrast, is based on two mutated repeats of the *LEU2* gene, one of which contains the mini-HO site, placed in an inverted orientation. This system allows both the physical detection of SCR intermediates (arising from unequal SCE in this case) as well as the genetic detection of Leu+ recombinant products (**Figure 2**) [23, 31]. In order to use these plasmids, the HO gene under the *GAL* promoter can be either integrated in the genome (such as in *ade3::GAL-HO* strains [23, 31] or expressed from a plasmid [32]. The strain to be studied should have the endogenous *LEU2* gene deleted as well as an ‘inconvertible' variant at the *MAT* locus (*MATa-inc* mutation) [33] that impedes HO cleavage. Importantly, although only the *pL2-HOr* allows the detection of equal SCE intermediates, the unequal SCE gave the same results as the equal SCE, concluding that unequal SCE in the pTINV system can be used an accurate indicator of the proficiency of total SCR [23, 31, 34].

Genetically, the frequency of SCR is assayed as the frequency of Leu+ recombinants after a five hours treatment with galactose (see detailed description in [29]) (**Figure 2A**). Briefly, the different wild-type and mutant strains transformed with the *pTINV* plasmid are grown either in glycerol-lactate or raffinose-containing media to ensure glucose consumption. Doxycycline is also added to the media to prevent transcription of the *leu2* repeats, which is driven by the *TET* promoter. Adding galactose to the media induces HO-induced expression. Spontaneous (at time 0) and HO-induced (five hours later) recombination frequencies are calculated by performing serial dilutions and counting the number of colonies that grow in total and recombinant-selective media (lacking Leucine). The median frequency of recombination is calculated for each of the cultures and the average of at least three independent transformants is usually considered as a reliable value.

In order to analyze the appearance and kinetics of SCR intermediates at the molecular level, a time-course experiment must be performed after the galactose addition (usually 0, 0.5, 1, 1.5, 2, 3, 4, 6, and 24 h). DNA samples from each time point are digested with *Xho*I and *Spe*I restriction enzymes and subjected to standard southern-blot hybridization with a *LEU2* specific probe (**Figure 2B**). At this step, the purity and specificity of the probe is essential to avoid hybridization with the endogenous genome or spurious intermediates. Upon *Xho*I-*Spe*I digestion, the HO-induced DSB results in 2.4 and 1.4 Kb bands while unequal SCE generates a head to head dimer that produces 4.7 and 2.9 Kb bands. Whereas the 4.7 Kb band is specific for unequal SCE, a 2.9 Kb product can also arise as a consequence of intrachromatid recombination. Therefore, SCR levels can be calculated as the ratio between the signal at the 4.7 Kb band and the total plasmid DNA (the sum of the signal at all bands). When the 2.9 Kb is sharp enough, the frequency of intrachromatid recombination can be estimated by subtracting the signal at the 4.7 Kb band from the signal at the 2.9 Kb band. This can give a measurement of the efficiency of recombination when it does not occur with the sister chromatid in our mutant conditions. In addition, general recombination assays are used to assay for the specificity of the SCR defect detected (see for example [32] or [35]). With these assays, in addition to the role of several general HR factors in SCR [34, 36, 37], specific factors required for SCR which so far do not affect HR between ectopic sequences or homologous chromosomes have been identified, such as cohesins, HST histone acetylases, the Rrm3 helicase, the Smc5-6 complex, etc [31, 32, 35, 38].

#### Monitoring recombination using direct repeat assays

Recombination can occur between sister chromatids, within a chromatid, between homologous chromosomes, or between repeated sequences at different chromosomal locations, called ectopic recombination. The ability to make direct repeat recombination reporters has allowed haploid cells to be readily screened for recessive mutations that alter recombination rates [39, 40]. While each recombination assay has its limitations, the use of direct repeat reporters has proved to be a powerful tool to determine mitotic gene conversion rates and to characterize mutations that increase or decrease gene conversion rates.

The standard reporter used for monitoring recombination between direct repeats is shown in **Figure 2C** [41]. The key features are 1) use of a nutritional selectable phenotype, here the ability to grow on medium lacking leucine, 2) different non-reverting mutations in the duplicated genes, and 3) inclusion of an additional selectable marker between the duplicated genes. We have routinely used duplications of the *LEU2* gene separated by a copy of the *URA3* gene with plasmid sequences adjacent to the *URA3* gene. The entire construct is integrated at the *LEU2* locus. To avoid interactions between the endogenous *URA3* locus and the *URA3* copy in the duplication, the endogenous locus allele should ideally be a deletion or if not, a non-reverting allele. The *LEU2* alleles are *leu2-BstEII* and *leu2-EcoRI.* Both were formed by fill-in synthesis and ligation following cutting with the restriction enzymes *Bst*EII or *Eco*RI of a copy of *LEU2* on a plasmid. The fill-in synthesis ablates the restriction site and causes a frameshift mutation in the *LEU2* gene. Reversions of the restriction enzyme site mutations are not detectable, and therefore do not confound low recombination rates. The *URA3* marker allows one to distinguish between various recombination events that result in a Leu+ phenotype, as shown in **Figure 2D**. These are gene conversion, crossovers between the two *leu2* alleles, and some cases of single strand annealing (SSA). Gene conversion only events are Leu+ Ura+, while crossovers and SSA events are Ura3- but may be Leu+ or Leu- (**Figure 2D**). For optimal detection of gene conversion events, it is important to have the *leu2* alleles separated within the *LEU2* gene by several hundred nucleotides. In the example shown here, the alleles are separated by about 600 nucleotides. If the alleles are too close together, many gene conversion events will cover both alleles (called co-conversion) and will result in a Leu- phenotype. Leu+ gene conversion events result from conversion of only one of the two alleles.

Recombination rates are determined by fluctuation tests [42]. Two basic approaches can be used, the use of the median as first described by Lea and Coulson [43] or from the p_0_ class, the number of cultures with no recombination events, adapted from the Luria-Delbruck fluctuation test [44] and updated by Lang and Murray [45]. Fluctuation tests are used to avoid the overweighted impact of “jackpot” events, colonies that experience a gene conversion event early in the growth of the colonies, such that a large proportion of cells are generated with the recombination phenotype but representing only one event. Use of the median or the p_0_ class eliminates this issue. Rates are determined from multiple colonies of the same genotype, and from these data either standard deviations or 95% confidence limits are derived.

When doing fluctuation tests by the median method, there is a limit on the range of rates that can be detected. The wildtype rate of gene conversion using the reporter shown in **Figure 2C** is on the order of 8 x 10^-6^. This can be readily detected using single colonies as the starting material. However, if rates are 10X lower, as occurs in homologous recombination mutants, this cannot be detected using a single colony as the starting material as a yeast colony has about 5 x 10^7^ cells. Instead the colony is used to inoculate a small culture, which is then grown overnight to generate more cells. The caveat here is that plating too many cells on the selection medium is inhibitory to growth of the rare Leu+ Ura+ recombination. To avoid this, the cells must be plated on several plates, with a maximum of 10^8^ cells spread on one plate.

Several other caveats regarding the starting strain need to be considered. First, some mutations that reduce recombination have intrinsic increased mutation rates. Thus, it is important to first confirm that the recombination reporter is intact and gives a Leu- Ura+ phenotype. On selection medium of -leu-ura medium, a wildtype colony when replica-plated from nonselective medium will give small areas called papillations of growth (**Figure 2E**). A mutant with a lowered recombination rate will give few to no papillations, while a mutant with increased gene conversion will give many more papillations. Loss of the reporter as shown in **Figure 2D** will result in no growth or complete growth on the medium and is not informative. To avoid the accumulation of additional mutations, particularly those that result in dysfunctional mitochondria and a petite phenotype, cells should first be passaged on plates with glycerol as a carbon source. This eliminates petites that have arisen in the strain. Another concern is the potential differential growth of parental versus recombinant cells. While this most likely is not a concern for recombination assays, if it seems that the recombinant segregants have a different growth rate from the parental strain, a modified median estimator can be applied as has been for estimation of mutation rates [46]. Lastly, if recombination rates are extremely high, such that the rate is 10^-3^ or higher, fluctuation tests are not valid as new events cannot be distinguished from progeny of earlier events. In this case other approaches must be used that measure events within one generation [47]. However, this is not a significant concern for gene conversion assays.

#### Ade2-based colony color sectoring assays to detect mitotic recombination

**Figure 3:**
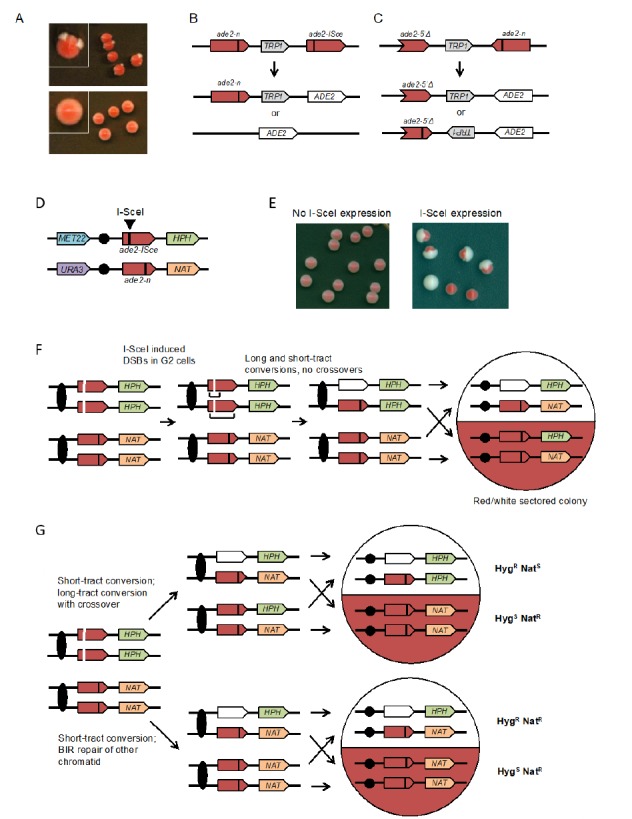
FIGURE 3: Colony color sectoring assays to detect spontaneous or DSB-induced recombination in haploid and diploids cells. **(A)** The upper panel shows wild-type colonies with the inverted-repeat reporter; *rad52Δ* (recombination-deficient) colonies are shown in the lower panel. **(B)** Direct-repeat recombination reporter: the *ade2-n* allele was generated by restriction enzyme fill-in of a *Nde*I site, the *ade2-ISce* allele was made by inserting the I-SceI cut site at the *Aat*II site. Gene conversion events retain the *TRP1* marker, whereas crossovers (CO) or SSA result in loss of *TRP1*. **(C)** Inverted-repeat recombination reporter: the *ade2-n* and *ade2-5′Δ* alleles are place in inverted orientation and separated by *TRP1*. Gene conversion events retain *TRP1* in the original configuration, whereas CO or long-tract sister-chromatid conversion flips the orientation of *TRP1*. **(D)** Diploid with *ade2-n* and *ade2-ISce* heteroalleles, heterozygous markers 150 kb downstream of the *ade2* loci and heterozygous markers on the other chromosome arm. **(E)** Examples of colonies before and after I-SceI induction. **(F)** I-SceI cuts both chromatids with the *ade2-ISce* allele in G2 cells and repair occurs by short or long tract conversion yielding *ADE2* or *ade2-n* allele, respectively. If both conversion events are non-CO, the colony is white/red sectored and the two halves retain heterozygosity for *HPH* (hygromycin resistance) and *NAT* (nourseothricin resistance). **(G)** If repair of one chromatid is associated with a CO and the recombinant chromatids segregate to different daughter cells, reciprocal LOH is detected. Note that if the recombinant chromatids segregate to the same daughter cell at mitosis, the CO is not detected and this must be taken into account when calculating the frequency of CO. BIR results in a half sector that retains heterozygosity and the other has LOH of the *HPH* marker.

Spontaneous mitotic recombination can be detected between chromosome homologs in diploids, or between artificial duplications in haploid or diploid cells. Because spontaneous mitotic recombination occurs at low frequencies, a selection step is generally required to detect it. In most assays, the two recombining sequences contain two different mutant alleles (heteroalleles) of a selectable gene to allow detection of rare recombination events during growth of a culture [48]. To provide a visual assay for mitotic recombination, several recombination reporters based on the *ADE2* gene have been developed [49–52]. Yeast *ade2* mutants accumulate a red pigment, resulting in red colonies, whereas cells with functional *ADE2* form white colonies. Diploid cells with *ade2* heteroalleles, or haploids with artificial *ade2* repeats, form mostly red colonies with rare recombination events detected as white Ade^+^ sectors or papillae (**Figure 3A**). The colony-sectoring phenotype provides a qualitative read out and has been useful to identify mutants with altered rates of recom-bination [50, 53, 54]. A fluctuation test can be performed to measure the rate of Ade^+^ recombinants within a culture.

The design of the assay consists of two recombination reporters with *ade2* heteroalleles placed in direct or inverted orientation on the same chromosome (**Figures 3B** and **3C**) [50–52]. Recombination can occur intra-chromosomally or between repeats of misaligned sister chromatids. For direct repeats, gene conversion events retain the intervening marker, while a crossover between repeats results in loss of the marker and one of the repeats. Deletion of one of the repeats and intervening sequence can also occur by single-strand annealing (SSA), a Rad51-independent mechanism. The inverted-repeat substrate was originally designed to avoid the contribution of SSA to recombination events. Conversion of *ade2-n* by the *ade2-5′Δ* allele restores *ADE2*. Around 50% of Ade^+^ recombinants exhibit inversion of the *TRP1* gene located between the heteroalleles; these events could occur by a crossover or long tract conversion between misaligned sister-chromatids [55, 56]. One of the challenges with using the *ade2* reporters is that the red pigment is slightly toxic; therefore, Ade^+^ recombinants have a growth advantage. To avoid this problem, it is important that colonies are picked after only three days growth on rich medium to perform fluctuation tests; if grown for longer, the apparent rate of recombination increases.

To facilitate analysis of unselected recombination events, a version of the direct repeat recombination reporter was generated with an I-SceI cut site inserted in one of the *ade2* repeats (**Figure 3B**) [51]. In these strains, I-SceI nuclease is expressed from a galactose-inducible promoter. The frequency of recombination is measured by the number of colonies that grow on medium containing galactose (I-SceI constitutively expressed) compared with the number on glucose-containing medium (I-SceI off). Most of the colonies that survive I-SceI expression are recombinants because imprecise NHEJ to eliminate the I-SceI site is very rare in yeast. Although most of the DSB-induced recombinants recovered on non-selective medium are Ade^+^, some are Ade^-^ due to copying the *ade2-n* mutation from the donor allele during recombination. Gene conversion products can also be detected in real time by Southern blot of genomic DNA digested with appropriate restriction enzymes [57].

#### Ade2-based assays to detect recombination between heteroalleles in diploid cells

The rate of spontaneous recombination between heteroalleles on chromosome homologs of diploid cells is ∼40-fold lower than observed for heteroalleles oriented as direct repeats in haploids [51]. Recombination between heteroalleles in diploid cells can occur by gene conversion with or without an associated crossover. Use of diploids with heterozygous markers on opposite chromosome arms allows detection of crossovers by loss of heterozygosity (LOH) for the marker centromere distal to the heteroalleles. However, LOH can also occur by break-induced replication and these events can only be distinguished from crossovers if both products of the daughter cells from the recombination event are recovered. To facilitate such analysis, an I-SceI site was incorporated into one chromosome homolog to enable analysis of unselected recombination events (**Figure 3D**) [49]. Induction of I-SceI results in a large increase in white and red/white sectored colonies (**Figure 3E**). LOH events can be detected by replica plating colonies to medium containing hygromycin or nourseothricin. Crossovers are detected by one sector that is Hyg^R^ Nat^S^ while the other is Hyg^S^ Nat^R^ (**Figure 3F**). BIR results in colonies in which one sector shows LOH for the *HPH* marker and the other retains heterozygosity (**Figure 3G**). If both broken chromatids repair by a crossover, the event cannot be distinguished from one with no crossovers. Colonies or half sectors that fail to repair a broken chromatid are detected by simultaneous loss of the *MET22* and *HPH* markers. The diploid assay shown can also be used to measure spontaneous (no I-SceI induction) LOH by selection for colonies that grow on 5-FOA-containing medium [49]. Events that are Ura^-^ Hyg^S^ Nat^R^ are due to chromosome loss, whereas Ura^-^ Hyg^R^ Nat^R^ events result from mitotic recombination.

#### Detection and analysis of mitotic recombination events

There have been three major challenges for the development of genetic assays of mitotic exchange: 1) if recombination events are resolved in G_2_ of the cell cycle, two daughter cells with recombinant products will be generated, and most selective methods detect only one of these products [58]; 2) the rate of spontaneous mitotic recombination events is four to five orders of magnitude less than for meiotic recombination [59], requiring selective or very efficient screening methods for detection; 3) most studies have been limited to analyzing a single genetic locus rather than a more global analysis of recombination events throughout the genome.

**Figure 4:**
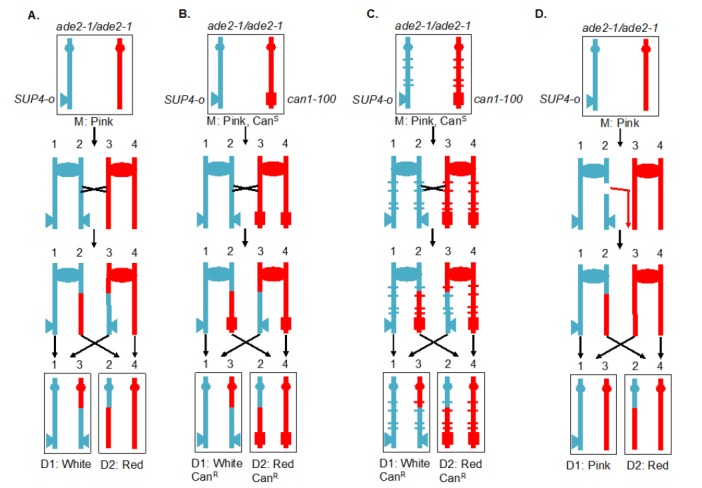
FIGURE 4: Recombination between homologous chromosomes during mitosis. Sectoring assay for monitoring mitotic crossover in the interval between the centromere (oval) and the *SUP4-o* insertion (triangle). Blue and red indicate the two homologs in the diploid cell. In this version of the assay, the markers are located on the left arm of chromosome V. **(A)** System for screening mitotic crossover by red/white sectoring assay. **(B)** Selection system of reciprocal crossovers. **(C)** Hybrid diploid strains with selection system for recovering reciprocal daughter cells after a crossover event, and with sequence polymorphisms (marked by blue and red ticks) for mapping positions of crossovers. **(D)** Detection of a non-reciprocal recombination event by a sectoring assay. In the diagram, a DSB on the chromosome containing the *SUP4-o* gene is repaired by a break-induced replication (BIR) event using the other homolog. The centromere-distal fragment containing the *SUP4-o* gene is lost, resulting in a pink/red sectored colony.

A selectable red/white sectoring system that largely overcame some of these challenges [59] is shown in **Figure 4A**. The starting diploid is homozygous for the *ade2-1* allele. This allele has a nonsense mutation at codon 65 that can be partially suppressed by the tRNA suppressor encoded by *SUP4-o*. In the absence of the suppressor, strains with the *ade2-1* mutation accumulate a red pigment, resulting in a red colony. A diploid with a single copy of *SUP4-o* results in partial suppression, producing a pink colony. If a crossover occurs between the centromere and the heterozygous *SUP4-o* marker and if the two recombined chromatids segregate into different daughter cells, one daughter cell will receive zero copies of *SUP4-o* (producing a red colony or a red sector) and the other daughter will receive two *SUP4-o* copies (producing a white colony or a white sector). Thus, if the crossover event occurs at the first cell division after plating, a red/white sectored colony will be formed. The two reciprocal products of the crossover can be purified from the two sectors. The system shown in **Figure 4A**, therefore, solves the first challenge described above. It should be noted that this system detects only half of the crossovers, since segregation of the two recombined chromatids into one cell and two unrecombined chromatids into the other, does not produce a sectored colony. The two different types of segregation are approximately equally frequent [60].

The system described above is a screen for recombination events rather than a selection. A modification of this system allows for selection of crossovers [59]. A diploid was constructed in which one copy of *CAN1* (a gene located near the left end of chromosome V) contained the *can1-100* allele, an ochre-suppressible mutation. The other copy of *CAN1* was deleted and replaced by *SUP4-o*. Strains with a functional *CAN1* gene are sensitive to the arginine analog canavanine. In the diploid shown in **Figure 4B**, the strain is sensitive to canavanine because the *can1-100* allele is suppressed by *SUP4-o*. In addition, as in the strain shown in **Figure 4A**, the diploid forms pink colonies because of the partial suppression of *ade2-1*. A crossover between the *SUP4-o/can1-100* markers and the centromere of chromosome V, followed by the appropriate segregation pattern, results in one cell that is canavanine-resistant (Can^R^) because it lacks the *SUP4-o* suppressor and one cell that is Can^R^ because it lacks the suppressible *can1-100* allele. Thus, mitotic crossovers are selected as Can^R^ red/white sectors (**Figure 4B**). This system can select for events that occur at a rate of <10^-6^/cell division.

Although the systems shown in **Figures 4A** and **4B** allow one to estimate the rate of crossovers between the *can1-100/SUP4-o* marker and the centromere, the location of the crossover within this interval is not determined. The system was slightly modified by constructing the diploid by mating haploid strains with about 0.4% sequence divergence (**Figure 4C**) [61–63]. In the resulting diploid, single-nucleotide polymorphisms (SNPs) occur at an average distance of <1 kb. In such a strain, when genomic samples are prepared from each side of a sectored colony, they can be analyzed by SNP-specific microarrays. In these arrays, each SNP is represented by four 25-base oligonucleotides, two with Watson and Crick strands of one allele and two with Watson and Crick strands of the second allele. Genomic samples heterozygous for a particular SNP hybridize about equally well to all four oligonucleotides, whereas samples with homozygous for one SNP hybridize better to one pair of oligonucleotides than the other. With the appropriate control DNA samples, such arrays can readily distinguish whether the sector is heterozygous or homozygous for each SNP. The position of LOH identifies the position of the crossover [62]. This type of analysis can be performed on sectored colonies to examine events on a single chromosome arm or, in strains with elevated levels of recombination events, throughout the genome. The pattern of crossovers and associated gene conversion events detected in such experiments can be revealing about the mechanisms of spontaneous and induced mitotic recombination [63–66]. Although many of the earlier experiments were done using SNP-specific microarrays, sequencing of genomic DNA isolated from the sectored colonies allows mapping of recombination events to even greater resolution.

Originally, the experiments were done on the left arm of chromosome V. Since this arm is relatively short (about 150 kb), it has a low rate of crossovers and a selective system for detection of the events is essential. It should be noted, however, that the selection system does not function well for every chromosome location. When the *can1-100* and *SUP4-o* genes were inserted near the right telomere of chromosome IV, the diploid had only partial sensitivity to canavanine [63], preventing an accurate measurement of the rate of Can^R^ red/white sectors. The red/white sectoring system, however, allowed non-selective screening for mitotic crossovers which occurred at a frequency of about 6 x 10^-5^/division. There are several advantages to using the non-selective red/white sectoring assay on chromosome IV. First, the right arm of IV contains about 1 Mb of DNA, roughly 10% of the yeast genome, whereas the left arm of chromosome V represents only 1% of the genome. Second, the non-selective method allows detection of recombination events that are non-reciprocal. For example, a break-induced replication (BIR) event on chromosome IV would produce a red/pink sectored colony instead of a red/white colony (**Figure 4D**). Such a colony would not be detectable by the selective method shown here. Third, unrepaired DNA lesions that stimulate recombination in the second division following cell plating can be detected by identifying a pink/white/red sectored colony [66].

The genetic systems described above have been used to map recombination events induced by gamma or UV radiation-induced recombination events [62, 65] and in mutants that have elevated levels of genetic instability. In the latter class are mutants that lack topoisomerase [67] or that have reduced expression of replicative DNA polymerases [68, 69]. In principle, these methods can be used to examine many genome-destabilizing conditions.

### Methods to detect gross chromosomal rearrangements

Gross chromosomal rearrangements (GCRs), including translocations, deletions, amplifications, and chromosome fusions, are believed to arise due to misrepair of DNA damage [70]. This DNA damage appears to result from cell metabolism, such as errors during DNA replication or reactive oxygen species but can also result from genetic defects that alter cell metabolism and exogenous sources of DNA damage. The rate of formation of GCRs is also influenced by features in the eukaryotic genome such as DSB-inducing sites and the presence of different types of repeated sequences; these genomic features also influence the structures of the GCRs that are formed (reviewed in [70]). Multiple pathways suppress the formation of GCRs, including pathways that are relatively specific for suppressing GCRs that result from different genomic features such as duplicated sequences. Stable GCRs are known to underlie a variety of genetic diseases [71], and the presence of GCRs and ongoing formation of GCRs are a characteristic feature of many types of cancers [72–74]. Moreover, mutations in a number of the genes encoding proteins that act in suppressing the formation of GCRs have been implicated as causal defects in inherited cancer susceptibility syndromes [70, 75].

**Figure 5:**
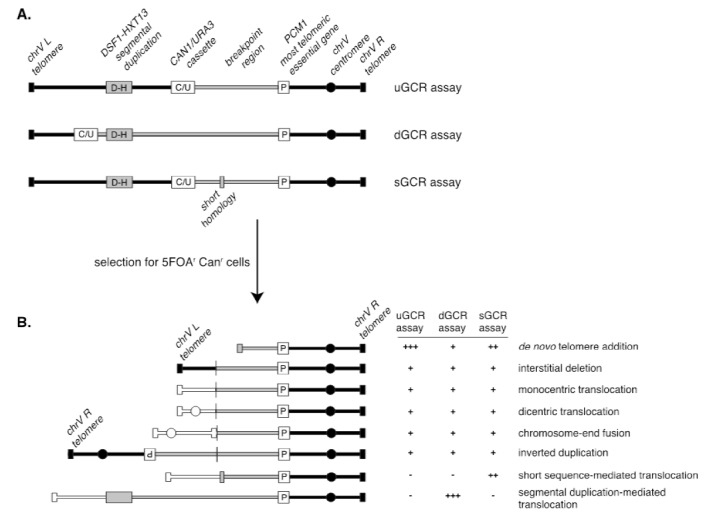
FIGURE 5. Assays to study gross chromosomal rearrangements. **(A)** Diagram of the uGCR, dGCR, and sGCR assays depicting the position of the *CAN1/URA3* counter-selectable cassette (white box labeled “C/U”) relative to the *DSF1-HXT13* segmental duplication (grey box labeled “D-H”), and the most telomeric essential gene *PCM1* (white box labeled “P”). The grey region of the chromosome corresponds to the breakpoint region, which lies between *PCM1* and the *CAN1/URA3* cassette and is the region where one of the breaks associated with the GCR must occur. The grey box in the sGCR assay corresponds to a region of short homology containing a repetitive tRNA and ∼100 bp of a repetitive delta sequence. **(B)** Selection of GCR assay-containing strains for resistance to 5-fluoroorotic acid (5-FOA) and canavanine (Can) selects for strains in which one of a number of GCRs has formed; these types of GCRs are selected at different rates in the different GCR assays. White chromosomes indicate translocations to other chromosomes (or alternatively different regions of the assay chromosomes).

A series of assays was developed for measuring the rates at which GCRs form in haploid *S. cerevisiae* strains to study the pathways that mediate the formation or suppression of GCRs [76–78]. These assays have two key features: 1) the assays are “undirected” as they do not require the formation of a specific GCR; and 2) the assays depend on loss of counter-selectable genetic markers present in non-essential terminal regions of chromosome arms, such as the left arm of chromosome V from *PCM1* to the telomere. The structures of three widely used GCR assay chromosomes are shown in **Figure 5**. A key feature of these assay chromosomes is a cassette containing two genes, *URA3* and *CAN1*, which confer sensitivity to 5-FOA and canavanine, respectively. In the unique sequence GCR (uGCR or *yel068c::CAN1/URA3*) assay, the cassette is inserted into a site centromeric to the *DSF1-HXT13* segmental duplication, whereas in the duplicated sequence GCR (dGCR *yel072w::CAN1/URA3*) assay, the cassette is inserted into a site telomeric to the *DSF1-HXT13* segmental duplication; the sGCR assay is the uGCR assay in which a region of short homology was inserted into the breakpoint region. The *DSF1-HXT13* segmental duplication is homologous to divergent regions on chromosomes IV, X and XIV. GCRs arise at low rates during the growth of cells containing these assay chromosomes that result in the loss of the left arm of chromosome V containing the *URA3 CAN1* cassette and healing of the apparently broken chromosome V by a diversity of DNA rearrangements that restore a functional telomere on the left end of chromosome V. Cells containing these GCRs can be selected by plating cultures on media containing 5-FOA and canavanine because GCR containing cells lack the cassette and are resistant to these two toxic compounds.

GCR rates are determined by fluctuation tests [79] using the method of the median [43], which avoids the impact of "jackpot" events due to the formation of a GCR early in the growth of a culture. Because of the low GCR rates of wild-type strains, large culture volumes (10-50 mL) must be plated onto selective media to obtain enough GCR-containing colonies from all of the cultures analyzed in an experiment to allow calculation of wild-type GCR rates. However, the dynamic ranges of the assays are very large, allowing analysis of mutants that have greatly increased GCR rates. For example, simultaneous loss of the *MEC1* and *TEL1* DNA damage checkpoint genes causes a >10,000-fold increase in the GCR rate, and loss of *ESC2*, which is involved in promoting sumoylation by the Smc5-6 complex, causes a >30,000-fold increase in the GCR rate [78]. The ability to analyze GCR rates has allowed the identification of many genes that function in the suppression or formation of GCRs and facilitated pathway analysis of these genes.

Because the GCR assays are undirected and can select for many different kinds of GCRs, structural characterization of the GCRs selected has provided important insights into the structure of GCRs and the mechanisms by which they are formed [70]. Multiple complementary strategies have been developed to characterize the structures of the rearranged chromosomes, including pulsed-field gel electrophoresis, PCR mapping, array comparative genomic hybridization, and multiplexed ligation-mediated primer amplification (reviewed in [70]). The adoption of whole genome sequencing (WGS), however, is rapidly supplanting these other methods, as WGS is fast and economical since many GCR-containing isolates can be multiplexed and sequenced simultaneously [79].The types of GCRs identified include terminal deletions healed by *de novo* telomere addition, interstitial deletions, monocentric translocations, dicentric translocations, dicentric inverted duplications (isoduplications), and dicentric chromosome end-fusions (**Figure 5B**; reviewed in [70]). Note that dicentric GCRs are unstable and undergo subsequent rounds of rearrangement in which the unstable dicentric GCRs are resolved into stable monocentric GCRs [70, 80]; the resulting GCRs can be quite complex.

A key feature of GCR assays is that they are strongly influenced by the chromosomal features present in the breakpoint region, which lies between the most telomeric essential gene and the most centromeric counter-selectable marker gene (**Figure 5A**). Numerous versions of the basic GCR assay have been developed by multiple labs to probe the effect of specific chromosomal features on the formation of GCRs by placing these features in the breakpoint region, including duplicated sequences, inverted repeats, trinucleotide repeat-containing sequences, G-quartet containing sequences, and HO sites (reviewed in [70]). The best-characterized GCRs assays are the classic assay, which are the uGCR assay, the dGCR assay, and the short-sequence homology (sGCR) assay (**Figure 5A**) [76–78]. In general, most mutations causing increased GCR rates in the classic, uGCR, and sGCR assays also cause increased GCR rates in the dGCR assays, but the converse is not true. Hence, the dGCR assay is more useful for screening for new GCR-inducing mutations than the other GCR assays. However, the structures of the GCRs selected in the dGCR assay are dominated by translocations formed by non-allelic recombination when the assay strains are recombination-proficient [78], and thus determining the structures of these GCRs is primarily only useful in providing insights into the control of non-allelic recombination and recombination between divergent DNA sequences (**Figure 5B**). In contrast, a much greater diversity of types of GCRs is selected in the classic and uGCR assays, and, in particular, in the sGCR assay (**Figure 5B**) [77, 80–82]; this makes this group of assays more useful in determining the effects of different mutations on the formation of a much greater diversity of types of GCRs.

### Methods to detect genome instability induced by repetitive sequences

#### Methods to detect the repeat expansions and the repeat-mediated genome instability using a yeast artificial intron

Expansions of simple tandem DNA repeats are responsible for the ever-growing number of hereditary genetic disorders in humans, such as fragile X syndrome, Friedreich's ataxia, Huntington's disease, myotonic dystrophy and many others [83, 84]. A startling feature of these mutational events is that the longer the repeat, the more unstable it is, which results in a progressively higher rate of its subsequent expansions or contractions [85]. Hence, these mutations are called dynamic DNA mutations [86], as opposed to classical static mutational changes in DNA. Studies conducted in many labs over the last two decades revealed that the instability of these repeats varies strongly depending on their sequence, length, location in the genome, ability to form alternative secondary structures, and the genetic background of the carrier cell/organism. It has also become clear that repeat expansions and other forms of repeat-mediated instability occur during practically all major DNA transactions, including replication, repair, recombination, and transcription [87–91].

**Figure 6:**
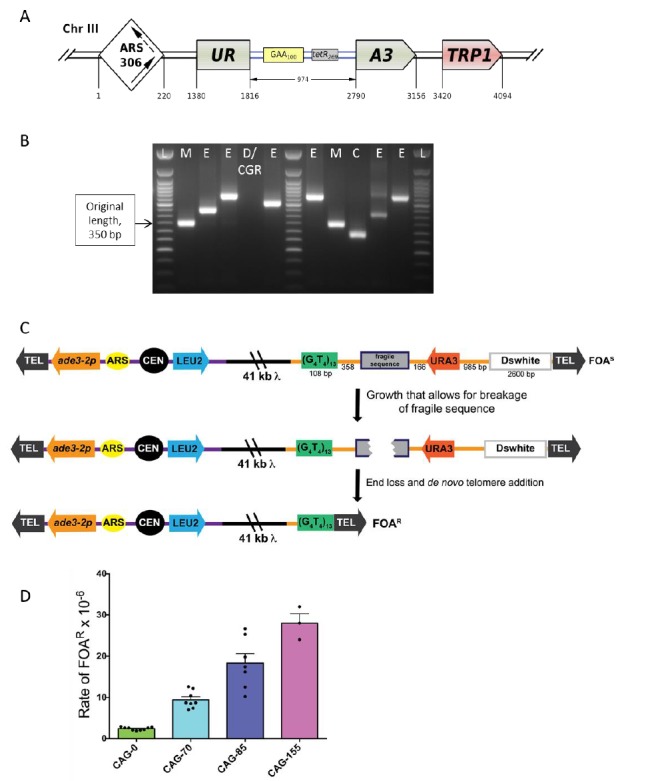
FIGURE 6: Scheme of the genetically tractable systems to study repeat expansions and repeat-mediated genome instability in yeast. **(A)** Scheme of the genetically tractable system to study repeat expansions and repeat-mediated genome instability in yeast. The selectable cassette contains flanking sequences from chromosome III, the *URA3* gene from the pYES2 plasmid (Invitrogen) (green), an intron sequence derived from the *ACT1* gene from chromosome VI (inside the *URA3* gene) (blue), part of the *tetR* coding region from the pACY184 plasmid (NEB) (gray), and the *TRP1* gene from the pYES3/CT plasmid (Invitrogen) (red). **(B)** Gel electrophoresis of the PCR analysis of 5-FOA^r^ colonies derived from a fluctuation test of a strain with 100 GAA repeats. L – ladder, M – mutation, E – expansion, D/CGR – deletion or CGR (complex genomic rearrangements), C – contraction with simultaneous repeat-mediated mutagenesis. The black arrow indicates a ∼350bp PCR fragment of the original (GAA)_100_ repeat. **(C)** Assay to measure chromosome fragility. In this system, a fragile sequence has been integrated onto a yeast artificial chromosome (YAC) between a telomere seed sequence (G_4_T_4_)_13_ and the *URA3* gene. Breaks that occur within the fragile sequence are subject to resection and telomere addition at the G_4_T_4_ sequence, which results in loss of the *URA3* gene and renders cells 5-FOA^R^. The YAC additionally contains a *LEU2* marker gene, which allows for maintenance of the YAC, a centromere (CEN4), an origin of replication (ARS1), yeast telomeres (TEL), a partially functional *ade3-2p* allele, and the *Drosophila* white gene. The orange line indicates pYIP5 plasmid backbone, the black line indicates lambda DNA, and the purple line corresponds to pUC18 plasmid backbone. **(D)** Wild-type fragility data of *S. cerevisiae* strain BY4705 where the fragile sequence integrated between the G_4_T_4_ and *URA3* marker is a CAG tract of the indicated length, in number of repeats. Assays done with this (CAG)n-URA3 YAC show that 5-FOA resistance increases with increasing number of CAG repeats. Data sourced from [107, 117, 365–367].

The lab of S. Mirkin developed a genetically tractable system to study repeat expansions and other types of repeat-mediated genome instability in budding yeast, *S. cerevisiae* [92, 93]. The key component of this system is a reporter carrying an expandable repeat within an intron of the artificially split *URA3* gene (**Figure 6A**). The reporter is linked to the *TRP1* gene to allow the whole cassette to be integrated into any *trp1* strain. Most studies were conducted for the Friedreich's ataxia (GAA)_n_ repeat. In this case, large-scale expansions that increase the intron's length beyond ∼1 kb inactivate the *URA3* gene by blocking its splicing, this allows us to detect expansion events on a selective media containing 5-FOA [92–94]. A remarkable characteristic of this system is that the longer the repeat, the more frequently it expands further [92, 95], mimicking the genetic anticipation phenomenon known for repeat expansion diseases in human pedigrees. Another important characteristic is that repeat-mediated *URA3* inactivation is a dynamic process as opposed to all-or-none mutational inactivation: the longer the expansion, the stronger the gene inactivation [92]. Consequently, clones with small-scale expansions are less resistant to 5-FOA than clones with large-scale expansions, resulting in smaller colonies on 5-FOA media. This makes it possible to adjust the concentration of 5-FOA to recover expansions of different scales. Besides repeat expansions, 5-FOA resistance can also result from point mutations or deletions in the body of the reporter, as well as from more complex genome rearrangements [92, 95–98]. The rates of the latter events are at least an order of magnitude higher in the presence of the (GAA)_n_ repeat than in its absence.

Given this complexity, a fluctuation test is first performed to determine 5-FOA-resistance (5-FOA^r^) rates for the (GAA)_n_ repeat in different genetic backgrounds. To distinguish between various repeat-mediated mutational events, PCR is then performed with primers flanking the repeat region, as shown in **Figure 6B**. Expansions are characterized by a larger PCR product. An unchanged or contracted repeat points to repeat-mediated mutagenesis, which is subsequently verified by Sanger sequencing of the reporter [95]. A lack of the PCR product is indicative of large deletions or complex genome rearrangements (CGRs), which are subsequently verified via whole genome Nanopore sequencing [96]. The rates of all three events: expansions, mutations and genome rearrangements, are then determined using the FluCalc program developed in the Mirkin lab, which is freely available at http://flucalc.ase.tufts.edu/ [99]. Note that to avoid statistical errors, one should carry out PCR and other analyses described above for all 5-FOA^r^ colonies that resulted from the fluctuation test. Practically, however, this is difficult to achieve in a fluctuation test, as the number of colonies on different 5-FOA plates can vary dramatically [44]. Thus, various approaches are employed in sampling the 5- FOA^r^colonies that are described in Radchenko *et al.* [99].

As discussed above, forward selection for the *URA3* reporter inactivation is used to detect repeat expansions in our system. Note, however, that it can be easily modified to study repeat contractions via reverse selection for uracil prototrophy. An example is a modified reporter with the (GAA)_128_ repeat within a longer artificial intron in the *URA3* gene [100]. Splicing of the latter reporter is impaired, which makes strains carrying it fully auxotrophic for uracil. If the repeat sequence in this reporter contracts significantly, splicing is re-established, so repeat contractions are detected on media lacking uracil.

This system can also be used to study genetic instability of other DNA microsatellites, such as expandable (ATTCT)_n_ repeats [101] or yeast interstitial telomeric sequences (TGTGTGGG)_n_ [98, 102]. These microsatellites inactivate the reporter when the length of the intron is significantly under its splicing threshold, showing that the mechanisms responsible for gene inactivation in our system differ depending on the nature of the repeat. Other mutational events, such as repeat-induced mutagenesis and CGRs were also observed for the latter repeats. Notably, studying yeast interstitial telomeric sequences using this system led to unraveling the mechanism of a very important class of chromosomal rearrangements: terminal inversions that occur during double-stranded DNA break repair via a single-strand annealing pathway [97].

Overall, the assay described here is a powerful and highly flexible and selectable system, which can be easily adopted to analyze various aspects of repeat-mediated genome instability. For example, an endogenous *URA3* promoter can be replaced with the inducible *GAL1* promoter to study the role of transcription [93]. The repeat itself can be inverted to study how genome instability depends on the repeat's orientation within the replication and/or transcription unit [100]. Alternatively, the whole cassette can be integrated in two orientations relative to the replication origin to study the effects of transcription-replication collisions [100]. Finally, the cassette can be integrated into a nonessential arm of chromosome V to investigate chromosomal fragility [103].

#### Utilizing a yeast artificial chromosome to study fragility of DNA sequences

Repetitive DNA sequences are common in eukaryotic genomes and are considered hotspots for breakage and genomic rearrangement in addition to the expansion described above. One reason that tracts of repetitive DNAs are thought to be prone to breakage is because they can form alternative structures, such hairpin loops, which can interfere with DNA replication and repair. Long tracts of CAG trinucleotide repeats are one well known type of repeat to form DNA secondary structures and are prone to expansion, contraction, and breakage. Expanded CAG repeats are responsible for over 14 genetic diseases, including Huntington's disease, myotonic dystrophy, spinocerebellar ataxias, and fragile X syndrome [104].

Fragile regions break and undergo aberrant repair at a higher frequency than the average DNA sequence, making it important to understand the mechanisms that promote and protect against breakage within these DNA regions. Teasing apart the cellular mechanisms that drive chromosome fragility has been a challenge, as breakage events that occur within the genome can result in loss of essential genetic material. To understand the role of DNA sequence on chromosomal breakage, an assay was developed to measure fragility of a desired sequence *in vivo* in *S. cerevisiae*. The assay utilizes a non-essential yeast artificial chromosome (YAC) which was originally designed by [105].

The YAC fragility assay is an effective way to measure breakage rates of potential fragile sequences as the YAC contains little homology to any of the natural yeast chromosomes, has no essential genes, and can withstand loss of genetic material. In the structure of the initial YAC, the (CAG)n-URA3 YAC, a CAG repeat tract has been integrated between a telomere seed sequence (G_4_T_4_)_13_ and a *URA3* marker gene (**Figure 6C**) [106]. Breakage that occurs within the CAG repeat can result in loss of the right arm of the YAC, rendering cells *ura3-* and resistant to 5-FOA. For YACs containing a CAG repeat, there is a length dependent increase in breakage rate as measured by 5-FOA resistance [106] (**Figure 6D**). The same assay has also been used for a variety of other fragile repeat sequences. For example, other derivatives of this YAC have been made that contain the CAG repeat in the opposite orientation (CTG)n [107], AT repeats that are a part of the human FRA16D common fragile site and stall replication [108], the expanded ATTCT repeat present in SCA10 patients [101], a short cruciform-forming inverted repeat [109], and an H-DNA-forming repeat from the human *c-MYC* gene that can form triplex DNA and is found at a translocation hotspot in Burkitt lymphoma [110].

In addition to the telomere seed sequence and the *URA3* reporter gene, there is also a *LEU2* marker gene on the left arm of the YAC, which allows for maintenance of the YAC, a yeast origin of replication (ARS1), and a centromere (CEN4), ensuring the YAC replicates and segregates during cell division. An *ade3-p* allele can be used to estimate the copy number of the YAC in *ade2 ade3* backgrounds; an occasional non-disjunction event can create a cell with two YACs, though the existence of these few cells do not significantly affect the assay and can generally be discounted. Since there is only one yeast origin on the YAC, the direction of replication through the fragile sequence is known. For the original (CAG)n-URA3 YAC, the CAG repeat tract was integrated such that the CAG repeat is on the lagging strand template, which has been shown to be the orientation less prone to repeat tract contractions [111]. An additional design consideration is the distance between the *URA3* gene and the telomere, such that the placement of the *URA3* gene is far enough away from the telomere not to be subjected to telomere position effect. For this reason, a buffer sequence is included between *URA3* and the telomere (the Drosophila white gene, **Figure 6C**) [105]. Outside of telomere position effect, point mutations in the *URA3* gene could generate 5-FOA resistant colonies that have not lost the right arm of the YAC. While this is a distinct possibility for certain gene deletions, PCR of the *URA3* gene from FOA resistant colonies will elucidate whether point mutation frequency is increased in a particular mutant (see [112] for an example). Alternatively, addition of a second marker gene to the end of the YAC can be utilized to eliminate events that are not due to end loss from the quantification. YACs have been constructed that contain both a *URA3* and *HIS3* marker [108] or both *URA3* and *ADE2* markers [113]. Addition of the *ADE2* marker allows a visual analysis of end loss events, which will generate red FOA^R^ colonies on FOA-Leu plates. Other derivatives of the YAC have been made that alter the level of transcription through the CAG tract by flanking it with either transcription terminators or addition of a galactose-inducible (pGal) promoter [112, 113].

A benefit of this system is the relative ease of the fragility assay. To begin, cells are plated for single colonies on yeast complete media lacking leucine and uracil (YC-Leu-Ura), which selects for colonies that have an intact YAC. For unstable repeats, colony PCR is used to amplify across the repetitive tract to ensure that starting colonies have the desired initial tract length. Typically, ten individual colonies (or portions of colonies) are each individually resuspended in YC-Leu liquid media. Cultures are grown for six to seven divisions which allows for breakage events to occur, and a portion of the culture is plated on media lacking leucine and containing 5-FOA. To obtain a total cell count, a portion of each culture is pooled, appropriately diluted and plated onto YC-Leu. Once colonies are counted, the rate of 5-FOA resistance is determined by using a fluctuation analysis such as the method of the median or the maximum likelihood method to avoid over-sampling of events that occur early in the culture [114]. Occurrence of YAC end loss can be stochastic, especially for mutants that have high rates of fragility, so it is best to repeat assays at least three times to obtain a standard deviation or standard error. If desired, the structure of the healed YACs can be analyzed by Southern blot analysis or sequencing [106]. A detailed methodology of how to perform the fragility assay was recently published [115].

One important feature of the YAC fragility assay is that a method of recovering the broken chromosome is built into the design. Proximal to the fragile sequences is a 108 bp back-up (G_4_T_4_)_13_ telomeric seed sequence from *Oxytrica* which has been shown to an efficient substrate for *S. cerevisiae* telomerase, but does not recombine with the natural yeast telomere sequence [116]. The G_4_T_4_ seed provides an efficient pathway for healing of breakage events. This is important because by providing an efficient method of healing, the primary variable influencing the rate of FOA^R^ is the rate of breakage. This aspect of the assay differs from another commonly used assay for end loss of chromosome V [77], where healing can occur by a variety of mechanisms including telomere addition onto short naturally occurring GT sequences or recombination with other chromosomes, thus a change in rate could be due to an effect on either breakage or healing. Since relatively few genes affect the efficiency of telomere addition this ensures that in most cases an increase in YAC end loss is caused by increased breakage rather than increased healing. This has the added advantage that the effect of DNA repair genes on chromosome fragility can be measured. For example, we have shown that many genes required for double strand break repair pathways, such as homologous recombination and non-homologous end-joining, are important in facilitating repair at an expanded repeat and preventing chromosome end loss [117]. Nonetheless it should be noted that the YAC fragility assay only measures the events that result in end loss after resection of DNA sequence to the (G_4_T_4_)_13_ telomere seed and telomere addition, not those that heal within the repeat without end loss, or those that fail to repair resulting in cell death. Marker loss assays underestimate the “true” rate of breakage but are useful for comparing between conditions.

In conclusion, the YAC fragility assay is a useful tool to quantitatively compare breakage rates of different sequences or of the same sequence in different conditions. As a powerful genetic assay, it can be used to identifying genes and pathways that are essential in preventing chromosome breakage. These studies have been instrumental in understanding how DNA repair and replication navigate difficult to repair and replicate sequences, which is important in our understanding of factors that influence chromosome fragility.

### Method to detect chromosomal rearrangements associated with gene amplification

It was only relatively recently that the large impact that structural genomic variation has on human genetic diversity and disease consequences has been fully appreciated [118, 119]. In addition, the realization that the rate of spontaneous (*de novo*) copy number variation (CNV) is quite high in humans [120] strongly suggested that our genomes are far more structurally plastic than previously thought. This was a significant conceptual shift, considering that, until about 15 years ago the prevalent view was that the primary nucleotide sequence of the DNA molecule alone was the main source of genetic diversity. Since then, epigenetic inheritance and structural genomic variation have come into focus as fields of investigation that are critical to our understanding of how the traits of living organisms are determined and passed along through generations. Both fields offer great opportunities for discovery, with significant gaps in knowledge yet to be filled.

A reflection of the late emphasis on CNV mechanisms is that there are relative few experimental model systems that have been developed to study this problem [70, 121], particularly in the context of diploid genomes. One of the ways to interrogate fundamental aspects of CNV mechanisms is through the detection chromosomal rearrangements associated with gene amplification. The amplification assay described here is based on a copy number reporter cassette that contains both the *SFA1* and *CUP1* genes that confer gene dosage-dependent tolerance to formaldehyde (FA) and copper (Cu), respectively. These strains have full deletions of the native *SFA1* gene, and a full deletion of the cluster of *CUP1* genes. Therefore, the only copies of *SFA1* and *CUP1* in the genome are those present in the reporter itself, which can be integrated at different sites of interest in the *S. cerevisiae* genome. The current version of the system is the result of improvements to previously described approaches [122, 123]. The earlier versions of this reporter contained the wild type *SFA1* allele, which was useful for identifying clones containing extra copies of the reporter. This version was not very effective for the quantitative gene amplification detection since it allowed a high number of small confounding background colonies to grow on selective media [123]. The improved strains carry the gene amplification reporter *SFA1*^*V208I*^*-CUP1-Kan*MX4. With the *SFA1-V208I* mutant allele, the selection for additional copies of the reporter is much tighter, supporting the effective quantitative measurement of gene amplification rates. Nearly all of the colonies that grow on the selective media contain two or more copies of the reporter (<2% false positives). Most selected clones carry just two copies of the region where the reporter is inserted, rather than higher order amplifications commonly detected by earlier versions [122].

**Figure 7:**
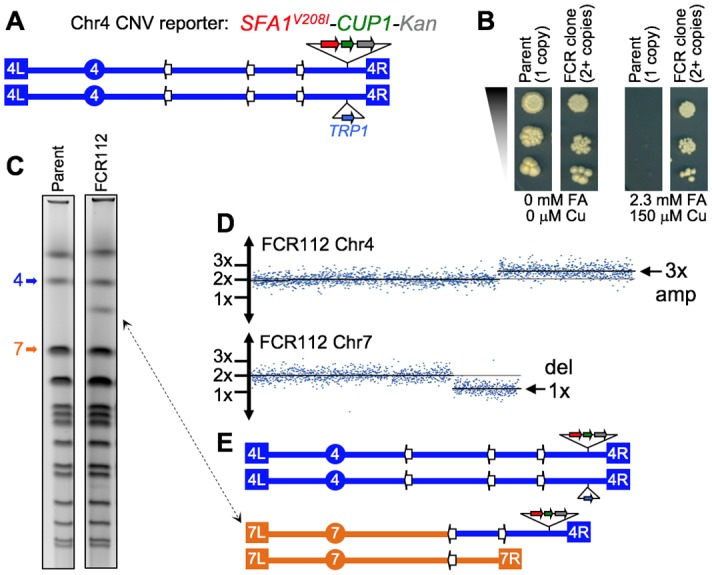
FIGURE 7: The diploid CNV assay. **(A)** Schematic map of Chr4 in a diploid test strain, showing telomeres (terminal boxes), centromere (circle), and Ty retrotransposon repeats (white arrows). One homolog has an insertion of the CNV reporter near the right end, the other has *TRP1* at the allelic position. **(B)** Reporter dosage-dependent phenotype. Cultures were serially diluted and spotted on plates. The parent strain is unable to form colonies on selective media (Copper [Cu] and Formaldehyde [FA]), while an FCR clone displays full viability. **(C)** Pulse field karyotypes showing a ∼1.3 Mb chromosomal rearrangement in CNV clone FCR112 (double-end arrow). The bands corresponding to normal Chr4 and Chr7 are indicated. **(D)** array-CGH data for FCR112. The plots show a 3x copy number amplification region on the right arm of Chr4, and a 1x deletion on the right arm of Chr7. The copy number breakpoints in both chromosomes correspond to the positions of Ty dispersed repeats. **(E)** Karyotype prediction for FCR112, showing a Chr7/Chr4 non-reciprocal translocation with a breakpoint junction at Ty sequences, suggesting a non-allelic homologous recombination mechanism of formation. Data credit to Ane Zeidler; full results to be described elsewhere (Zeidler, Argueso, *et al., in preparation*).

The diploid experimental strain shown in **Figure 7A** has one insertion of the reporter cassette on the right arm of chromosome 4 (Chr4). Parent cells carrying a single reporter copy are unable to grow on media containing high levels of FA and Cu. However, mutant cells carrying a chromosomal rearrangement resulting in two or more copies of the reporter display resistance to the combined inhibitors, and therefore are able grow and form colonies on a Petri dish. These CNV-carrying clones are labeled FCR, for Formaldehyde and Copper Resistant (**Figure 7B**). In addition, the diploid parent strain contains an insertion of the *TRP1* marker on the other homolog of Chr4 at a position allelic to the CNV reporter (**Figure 7A**). By selecting FCR clones on media containing FA and Cu but lacking tryptophan, it is possible to eliminate false positive cells that may duplicate the reporter through a mitotic crossover leading to LOH. This system is capable of identifying spontaneous chromosomal rearrangements resulting in a simple doubling of any region of interest in a diploid yeast cell's genome.

The use of this assay typically starts with a quantitative analysis of the gene amplification rate by counting the FCR colonies that grow on FA+Cu Trp drop-out plates. The basal (spontaneous) CNV rates range from ∼10^-7^ to ∼10^-6^ amplifications/cell division, depending on the genomic context of the reporter insertion. Changes to the basal CNV rates caused by environmental exposures or by genetic defects can be determined by exposing the parent cells to mutagens prior to selective plating or by mutating candidate genes of interest.

Once the amplification rates have been calculated, the next step in the analysis is the qualitative characterization of the chromosomal rearrangements leading to amplification of the reporter. This is done to obtain insight into the repair pathways involved in the formation of CNVs. The *SFA1-CUP1* experimental system has two attractive features that distinguish it from other approaches used to study chromosomal rearrangements in budding yeast. First, the initiating DNA lesion that triggers the chromosomal rearrangement does not necessarily need to occur near the reporter cassette. In fact, the initiating lesion is often a double strand break in a different chromosome. Second, using diploid cells allows for sampling of a diverse spectrum of genome rearrangements, including large deletions that are not typically viable in haploids. These two key benefits are illustrated by the karyotype of clone FCR112. Pulse field gel electrophoresis (PFGE) showed a new chromosome of ∼1.3 Mb in this clone (**Figure 7C**), and array-based comparative genomic hybridization (array-CGH) identified an amplification on the right arm of Chr4 associated with a deletion on the right arm of Chr7 (**Figure 7D**). The combined size of the preserved segment of Chr7 and the amplified segment of Chr4 is 1.3 Mb, suggesting that the new chromosome seen by PFGE is a Chr7/Chr4 non-reciprocal translocation (**Figure 7E**). Further tests (PCR, DNA sequencing) were then conducted to validate this prediction, and to determine the molecular CNV mechanism through fine breakpoint mapping. In this case, the mechanism was non-allelic homologous recombination (NAHR) between dispersed Ty1 retro-transposable repeats. The translocation detected in clone FCR112 was formed by a cellular pathway essentially identical to that recently reported to be a major driver of CNVs in humans - NAHR between human short dispersed repeats [124–126].

The *SFA1-CUP1* system has been used to select and analyze more than 300 different FCR clones, derived both spontaneously, and induced by mutagens or derived from DNA repair defective mutant backgrounds. A broad range of CNV rates were observed, associated with a diverse spectrum of CNVs including segmental duplications, translocations, and aneuploidy. The molecular mechanisms inferred from DNA sequences present at breakpoints were NAHR in most cases, but also some created by non- or micro-homology pathways (NHEJ and Micro-homology Mediated Break-Induced Replication [MMBIR])[127]. The full results derived from this work will be described elsewhere (Zeidler, Stanton, Sharif, Argueso et al., *in preparation*).

Most of the cautionary notes that should be considered when using the *SFA1-CUP1* reporter pertain to measures that must be taken to ensure that the selection is tight and that no false clones (without reporter amplification) are detected. The FA+Cu selective plates should be incubated for five days at 30°C inside a large closed plastic box with a couple of moist paper towels inside. This is done to prevent the media from drying and cracking during the incubation. The first FCR colonies are visible after three days, the last ones appear on day 5. A high plating density can also lead to background growth. The number of cells per FA+Cu plate should not exceed 10^6^ cells per normal size plate (90 mm diameter). If the frequency of amplification in the strain of interest is low, users need to plate multiple plates at ∼10^6^ cells/plate density. Plating at higher density (>10^7^ cells/plate) may in some cases lead to growth of false positive colonies that do not carry amplification of the reporter but manage to grow on selective media due to a “filtering” effect on top of a layer of dead cells. Finally, the formaldehyde in the plates tends to lose potency over time, therefore, fresh FA+Cu plates should be prepared the day before each plating experiment.

As described above, the combination of concentrations of FA and Cu is a critical parameter for the successful use of this assay. A given pair of concentrations may work very well for a specific strain (such as the example in **Figure 7B**) but would likely not be effective for a different one. This means the combination of concentrations needs to be re-optimized whenever using strains with the reporter inserted at a different genomic position. Likewise, if the reporter is transferred to a different genetic background it will be necessary to test a range of concentrations to find which are best suited to the new strain. Finally, specific users will presumably want to introduce mutations into their respective wild type strains (*e.g. pol32*Δ*, rad51*Δ, etc), and that will also require them to re-calibrate the ideal FA+Cu concentration combinations. The re-optimization of the selection concentrations should be done to find the minimal concentration combination that fully inhibits the growth of a strain with one copy of the reporter. Once a few resistant clones are selected and confirmed to carry two copies of the reporter, they can then be used as positive controls to fine-tune the ideal concentrations for the assay. It is also sometimes necessary to adjust the FA concentrations when a new bottle of formaldehyde is received, as it may be slightly more or less potent than the previous stock solution.

In addition to pre-selection considerations, it is also important to keep in mind that it is common to observe different levels of FA+Cu resistance between the clones that are selected in this assay. Clones with more than 2 copies of the reporter are always more resistant, but these multi-copy events are not very frequent (5-10%). Most resistant clones only have two copies of the reporter. Most of the difference in resistance between clones is due to the nature of the chromosomal rearrangement present in each of them. Some rearrangements make the cells grow slowly (regardless of FA or Cu) while others do not appear to interfere with growth.

In summary, the *SFA1-CUP1* amplification assay offers attractive and distinctive features for the study of CNV mechanisms. However, it is somewhat laborious and more susceptible to leaky selection than other widely adopted approaches. Specifically, the haploid GCR assay is simpler and more robust from the experimental standpoint and is thus better suited for high throughput studies [128]. The *SFA1-CUP1* assay is a highly valuable approach to take to answer a narrower set of questions, particularly those involving the role of ploidy. Used in combination, these two assays provide a powerful tool kit for answering a wider variety of questions about the mechanism of formation of chromosomal rearrangements.

## MOLECULAR BIOLOGY ASSAYS TO DETECT DNA BREAKS AND REPAIR

Living cells constantly experience chromosomal lesions that derail the duplication of genome. Some of these lesions, such as DSBs, are particularly detrimental in that they, through chromosomal fragmentation, block both chromosomal replication and segregation and also cause loss of genetic information, — all resulting in cell death. Chromosomal DSBs threaten genome integrity and provoke rearrangements that are the hallmark of cancer cells.

Breaks can be repaired by two fundamentally different pathways: NHEJ or HR. NHEJ is intrinsically mutagenic, since most events that ligate ends back together result in deletions of varying size or in small insertions. NHEJ itself can be subdivided into two pathways, termed “classic” and “alternative” (or microhomology-mediated end-joining) that differ both in their genetic requirements and especially in the small number of bases that need to be paired at the junctions.

In contrast, HR relies on repairing the DSB by copying sequences from an intact template. The most precise of the HR processes is gene conversion by synthesis-dependent strand annealing (SDSA), in which the break is repaired by a short “patch” of DNA copied from a template (gene conversion), that can be associated or not associated with crossover. A distinct, but related process, involving the formation of a double Holliday junction intermediate (dHJ), also produces a patch of newly copied DNA but often is accompanied by reciprocal exchange between the DNA segments containing the donor and recipient. SDSA and dHJ mechanisms require that both ends of the DSB share sufficient homology with the donor to effectively repair. If only one end shares enough homology, a break-induced replication (BIR) process is launched, copying a template chromosome all the way to its telomere. BIR thus can result in non-reciprocal translocations. A fourth HR process is single-strand annealing (SSA) in which a deletion is formed by 5′ to 3′ resection of DSB ends and annealing of homologous sequences flanking the DSB. Much of our understanding of DSB formation as well as of both NHEJ and HR has come from studies in bacteria and yeast.

In this section, the molecular biology assays in *E.coli* and yeast designed to detect DSBs and their repair are described (Box 2).

BOX 2: **MOLECULAR BIOLOGY ASSAYS TO DETECT DNA BREAKS AND REPAIR**

**Detection and quantification of DNA breaks by pulsed field gel electrophoresis |** Assays for physical detection of DNA breaks. The methods can detect low abundance of breaks and be quantitative.

**Introduction of site-specific DNA breaks and analysis of repair |** A combination of genetic and physical assays can monitor break formation and repair in real time.

**Quantification of strand-specific single-stranded DNA |** Assays for measurement of single stranded DNA formation in real time. These assays measure DNA unwinding or resection at a double-strand break.

**Detection of toxic recombination intermediates |** Recombination intermediates arising from excess single-stranded DNA can become toxic if not correctly resolved. These assays monitor formation of these toxic intermediates.

**Detection of hypermutable single-strand DNA in living cells |** Single-stranded DNA that occurs during the repair process of a DNA lesion can be a source of mutation, arising from error-prone replication across a damaged template. The sources of single-stranded DNA and the mutagenic processes can be detected by genetic reporters and physical methods.

### Detection and quantification of DSBs by pulsed field gel electrophoresis

When DSBs are initiated by a site-specific endonuclease, it is relatively easy to detect their formation by using Southern blot analysis and a radioactively labeled probe specific for the DSB site (reviewed in [129–131]). DSBs initiated at random places in genomic DNA are more difficult to detect. They are frequently reported by methods like neutral sucrose gradient centrifugation [132], neutral elution [133], comet assay [134], or DNA diffusion assay [135]. Unfortunately, these methods are either suboptimal for the quantification of the real density of DSBs or are purely qualitative. In contrast, PFGE offers a straightforward way to quantify DSBs with at least five orders of magnitude dynamic range (if DNA is radiolabeled and phosphorimaging detection is used) [136, 137]. The range of resolution and linearity of quantification makes the technique especially useful for DNA damage studies of prokaryotes harboring circular genomes. Here the detection of DSBs by PFGE is described.

PFGE was developed to resolve chromosome-sized linear DNA molecules and has been used extensively since its inception in genome characterization [138–143], and epidemiological studies [144–146]. The technique is highly versatile and can be used to separate linear DNA ranging from 30 kbp to 12 Mbp [147, 148], making it an excellent tool for general genome characterization in bacteria and low eukaryotes, and the standard approach for molecular genotyping, when used in combination with rare-cutting restrictases [149, 150]. Furthermore, the technique is uniquely suited for visualization and quantification of sub-chromosomal fragments in organisms harboring circular chromosomes, like most prokaryotes, because not only circular DNA in general moves slower in agarose than linear DNA of the same size [151–154], but also circles longer than 30 kbp do not even enter pulsed-field gels [155, 156], keeping the intact chromosomal DNA in the wells [157]. Previously, PFGE has been used to visualize randomly distributed DSBs induced by gamma-irradiation in the yeast *S. cerevisiae* [158]. Here we describe the use of PFGE to characterize formation of DSBs in bacteria. In particular, PFGE is used to characterize spontaneous chromosomal fragmentation due to various defects in the chromosomal metabolism [159–165] or fragmentation induced upon exposure of bacterial cells to various clastogens [166–170].

**Figure 8:**
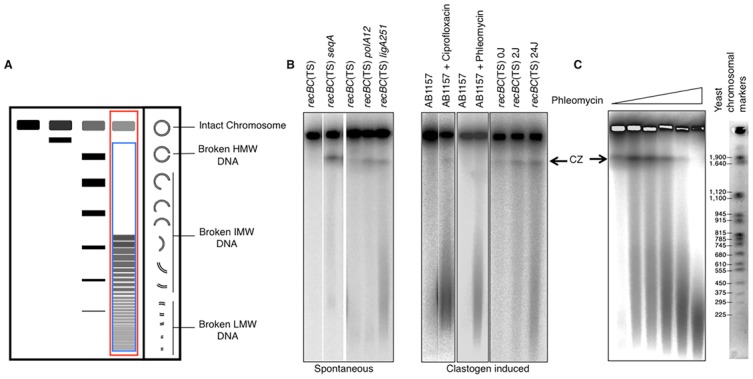
FIGURE 8: Fragmentation of bacterial chromosomes under various conditions. **(A)** Schematic representation of size-dependent migration of linear DNA through PFGs. Note that the intact circular chromosome stays in the wells. Chromosomal fragmentation is quantified as the percentage of the total signal (well+lane, red rectangle) that enters the lane (blue rectangle), that is: [Signal in lanes x 100 / total signal (well+lane)]. HMW, high molecular weight (≥2 mbp); IMW, Intermediate molecular weight (0.2 - 2.0 mbp); LMW, low molecular weight (≤0.2 mbp). **(B)** Spontaneous and clastogen-induced chromosomal fragmentation in *E. coli*. Typically, chromosomal fragmentation is detected in the *recBC* mutant, deficient both in repair of DSBs and in degradation of linear DNA. However, when fragmentation is massive, it can be detected in wild type cells (such as ciprofloxacin- or phleomycin-induced fragmentation in AB1157 in these examples). Spontaneous fragmentation is measured under conditions when the *recBC*(Ts) mutants carry additional defects in SeqA, DNA polymerase I or DNA ligase [165, 167]. Clastogen-induced fragmentation is observed upon exposure to phleomycin and ciprofloxacin. Ultraviolet light, which affects only one strand of DNA, still causes significant fragmentation in *recBC*(Ts) background [166]. CZ, compression zone. **(C)** Pulsed field gel electrophoresis can be used to calculate the density of breaks in the chromosomal DNA if the average size of the resulting chromosomal fragments is measured.

Continuous electric field resolves DNA molecules based on size as long as they are small and are able to sieve through agarose gel matrix. As the size of DNA increases, it begins to reptate (moving in a snake-like fashion), causing decrease in sieving and making DNA move with a size-independent velocity. The size-velocity correlation is ultimately lost in regular agarose gels when the size of DNA reaches 50 kb [133]. Extremely low concentration of agarose separates DNA up to 170 kbp and even ∼450 kbp in length, but the fragility of gels and long-time of electrophoresis makes these gels impractical [171, 172]; besides, they still cannot separate chromosome-sized molecules. PFGE overcomes the limitation of continuous electric field gel electrophoresis by periodically changing the directions of the electric field. Such a maneuver forces DNA molecules to change orientations, as they move through the agarose, resulting in efficient, size-dependent separations of linear DNA pieces as long as 12 Mbp [147]. The movement of DNA through the PFGs depends upon a variety of factors including field strength, pulse time, gel concentration, and time of electrophoresis. By varying these conditions, PFGE can be customized to resolve specific size ranges of DNA molecules. As circular DNA, including large plasmids, stays in wells, while the linear fragments enter the gel and resolve based on their sizes, the separation of linear and circular DNA during PFGE becomes a binary system (**Figure 8A**). The separation is especially effective for the smaller molecules (20-500 kbp), the sizes of which can also be used to calculate the density of DSBs in the chromosomes [170].

The measurement of chromosomal fragmentation is performed in three steps. In the first step, chromosomal DNA of the growing cultures of *E. coli* is labeled with ^32^P-orthophosphoric acid, and bacterial cells are embedded in agarose plugs and lysed to release the cell constituents. In the second step, the plugs are loaded onto agarose gels and subjected to PFGE using the conditions specific for separation of linear DNA in a particular (broad) size range (for example, 20-600 kbp, or 300-3,000 kbp, or 1-10 Mbp). In the final step, the gels are dried under vacuum, subjected to phosphor-imaging and quantified using phosphor-imaging software.

Using PFGE, spontaneous chromosomal fragmentation was detected in *recBCD* mutant, in which DSBs persist [161, 166]. Combining Δ*seqA, polA12*(Ts), and *ligA251*(Ts) mutations with *recBCD* defects increased the chromosomal fragmentation (**Figure 8B**), revealing the cellular roles of SeqA, DNA polymerase I and DNA ligase in the avoidance of spontaneous DSBs in *E. coli* [167].

Treatment of growing cultures of *E. coli* with clastogens also causes chromosomal fragmentation that can be detected and quantified by PFGE (**Figure 8B, C**). As expected, increasing the amount of damage, either by increasing the dose or time of exposure, led to higher number of DSBs, resulting in ever smaller sub-chromosomal fragments (**Figure 8C**). Interestingly, clastogens that affect only one strand of DNA, such as UV, also cause fragmentation of chromosomes in growing cells [166] (**Figure 8B**).

PFGE is an excellent tool to quantify chromosomal fragmentation resulting from DNA damage. However, the technique has its own (minor) limitations. First, branched DNA structures, including replication and repair intermediates, do not enter the gel [173]. If a significant fraction of these is suspected, treatments of DNA in agarose plugs with enzymes, such as T7 debranching endonuclease, may facilitate their migration into the gel. Second, presence of nicks and gaps may cause entrapment of DNA in the wells and may require longer electrophoresis at reduced field strength to facilitate their migration in the gels [167]. Third, high molecular weight linear DNA with nicks has been shown to break during PFGE, thereby inflating the density of DSBs [167]. In some cases, when sharp banding pattern is lost due to degradation, inclusion of thiourea in electrophoretic buffer may help to preserve the integrity of DNA [174]. An important point when lysing bacteria during the preparation of the chromosomal plugs is to minimize artefactual fragmentation during lysis. We have recently shown that inclusion of a non-specific RNase treatment during preparation of plugs causes extremely high spontaneous fragmentation by activating the surface endonuclease of *E. coli*, Endo I [175]. Therefore, RNase treatment should not be used during preparation of plugs, at least with bacterial cells.

Currently PFGE, in combination with labeling chromosomal DNA with ^32^P, is a gold standard for determination of chromosomal fragmentation in *E. coli*, offering excellent range of separation and exceptional dynamic range of quantification. Minor changes in plug preparation make the technique equally suited for use with Gram-positive bacteria, including pathogenic *Mycobacteria*. Since chromosomes can be also radiolabeled with ^3^H thymidine, the technique can be used without phosphorimaging, by simply slicing the gels and subjecting gel slices to scintillation counting [157].

### Analyzing the repair of site-specific chromosome breaks in yeast

#### Induction of site-specific DSBs and analysis of their repair in budding yeast

**Figure 9:**
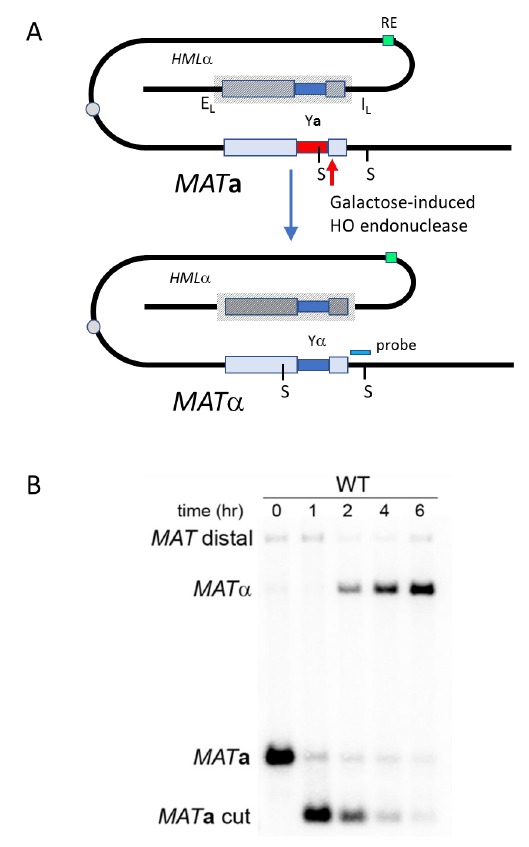
FIGURE 9: Mating-type gene switching in budding yeast. **(A)** Switching of *MAT***a** to *MAT*α by homologous recombination between the HO endonuclease-cleaved *MAT***a** locus and the *HML*α donor locus. Part of chromosome III is shown. *HML*α and *MAT***a** are each about 100 kb from the centromere (circle). *HML*α and the other donor, *HMR***a** (not shown, at the opposite end of the same chromosome), are bounded by silencer sites (E and I) that create a highly ordered nucleosome structure (hatched lines) that makes the donors transcriptionally silent and prevents HO cleavage at the same α or **a** sequences that are present at *MAT*. A Recombination Enhancer (RE) facilitates the use of *HML*α through Fkh1 protein binding both to RE and to sites near the DSB. *Sty*I sites are indicated by S. **(B)** Kinetics of *MAT* switching. The cleavage of *MAT***a** is complete after inducing HO endonuclease for 60 min. Cleavage and subsequent steps are assayed on a Southern blot, using a *MAT*-distal sequence as probe (green). HO-induced cleavage of *MAT***a** is followed by the appearance of *MAT*α, which has different *Sty*I restriction sites and is thus easily distinguished from *MAT***a**. Here, the *HML*α donor also harbors a single-base pair mutation that prevents subsequent HO cleavage.

Much of our understanding of both NHEJ and HR has come from studies in the budding yeast, *S. cerevisiae*, where DSBs can be induced in virtually all the cells of the population in a highly synchronous fashion, by placing the site-specific HO endonuclease under the control of a galactose-inducible promoter. In most such experiments, *GAL::HO* is integrated at the *ade3* locus [176]; HO induction is rapid once 2% galactose is added to cells grown in medium containing raffinose or lactate as a carbon source. The most widely used assay is the switching of budding yeast mating-type genes, from *MAT***a** to MATα (shown in **Figure 9A**) or from *MAT*α to *MAT***a**, using the opposite donor sequence, *HMR***a** (not shown) [177]. The efficiency of HO-induced cleavage can be monitored on a Southern blot by using a *MAT*-distal probe (**Figure 9B**).

In cells in which both the *HML*α and *HMR***a** donor loci are deleted, cells will die unless repair occurs through NHEJ. Because HO endonuclease is rapidly degraded, by briefly inducing HO, one can examine the perfect re-ligation of the 4-nt, 3′ overhanging ends by the re-formation of the StyI *MAT***a** band, as well as by measuring viability. When HO expression is continuous, then viability drops to about 0.2% and all the survivors have an insertion or deletion, created by NHEJ, altering the cleavage site [178].

In strains that carry the *HML*α and *HMR*a donors, it is easy to follow repair by homologous recombination real time, by a combination of genetic assays and physical monitoring of DNA (Southern blots and PCR). As shown in **Figure 9B**, HO cleavage produces a smaller *Sty*I fragment homologous to the *MAT*-distal probe. After a delay of an hour, the gene conversion product, *MAT*α, becomes visible, because the α sequences lack a *Sty*I site present in the **a**-specific sequences and hence the product is a larger, easily identified band; other restriction sites can also be exploited [179]. By similar strategies, and inserting HO cleavage sites in other genomic locations, one could follow the kinetics of SSA [180] or BIR [181]. A galactose-inducible I-SceI endonuclease, assayed in the same ways, demonstrated that the outcomes were not dependent on a particular nuclease [182].

The consequences of deleting different repair and replication-associated genes can then be assessed. It is also possible to identify intermediates of repair, such as the 5′ to 3′ resection of DSB ends [183]. The advent of chromatin immunoprecipitation made it possible to follow the recruitment of the Rad51 recombination protein and other proteins to the site of the DSB [184]. These results inaugurated the age of “*in vivo* biochemistry” ( reviewed in [185]).

Over the past 3 decades, various studies have defined the roles of many DSB repair and replication proteins in each of the processes enumerated above. These studies have been recently augmented by using inducible Cas9 site-specific nucleases to cleave DNA. As genetic modifications have become easier, several other questions have been explored in greater detail.

Using a BIR system in which homology is confined to 108 bp it has been asked: How tolerant is the Rad51 filament to carry out strand exchange with divergent substrates? It was found that even substrates with every 6^th^ base mismatched are able to be used, albeit at only about 5% of the rate of fully matched sequences [186].

By placing donor sequences, sharing 1 kb homology on either side of a DSB, it was enquired how the efficiency of DSB repair depends on the proximity, as measured by contact probability using chromosome conformation capture data. It was shown that there is a very strong correlation between the likelihood of collision and the efficiency of repair. Other performed experiments suggested that only about one in four collisions is likely to lead to a successful repair event [187, 188].

By changing the lengths of homology shared by the HO-cleaved target and a donor and by exploiting a “Recombination Enhancer” (RE in **Figure 9A**) that binds near the DSB and thus drags a nearby donor near the break, it was shown that the ability of a 35-bp homologous sequence to initiate repair was greatly improved by tethering a distant intrachromosomal donor near the DSB [189].

By deleting or overexpressing Sgs1^BLM^ and Mph1^FANCM^ helicases it became possible to demonstrate their synergistic and distinct roles in promoting or discouraging BIR and “second end capture” during gene conversion [189, 190].

By creating a counter-selectable reporter gene in a donor locus it was discovered that gene conversion repair of a DSB is 1000 time more mutagenic than simple replication of the same sequences [191, 192]. It was demonstrated that the principal causes of these mutations were replication-slippage and dissociation/reassociation of DNA polymerase δ during the copying of the donor sequences. Similar template jumps are found in BIR [193].

With the advent of CRISPR/Cas9 it is possible to compare and contrast the consequences of generating (usually) blunt ends with HO cleavages with 4-bp 3′ overhanging ends. Such studies are now ongoing.

#### DNA DSB induction and repair in fission yeast

Synchronous induction of a single genomic DSB offers one of the most effective ways to follow DNA recombination in cells. In budding yeast, induction of HO and I-*Sce*I endonucleases was used for decades to follow DSB repair by NHEJ and HR [194]. These studies uncovered molecular mechanisms of DSB repair pathways and the function of proteins involved. Similar approaches were subsequently developed in other organisms including human however often with less efficient and less synchronous DSB induction.

**Figure 10:**
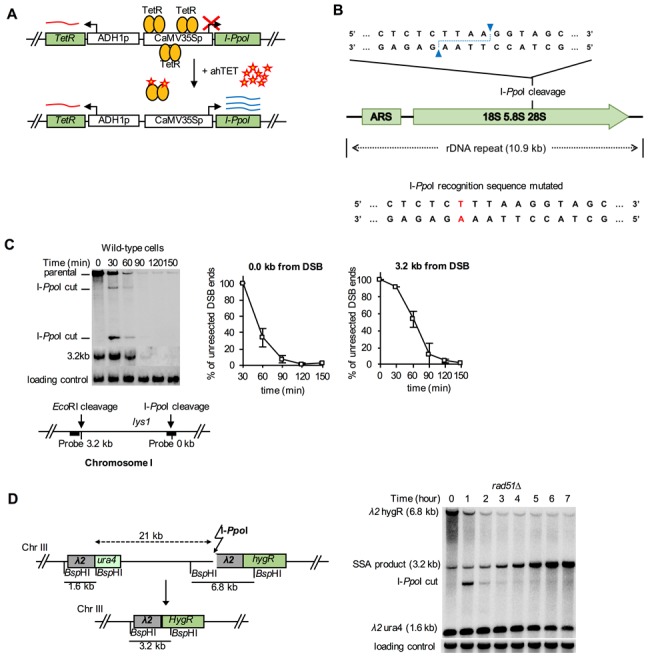
FIGURE 10: DSB induction and repair in fission yeast. **(A)** Inducible I-*Ppo*I system. I-*Ppo*I is under the control of an anhydrotetracycline (ahTET)-inducible promoter. The *ADH1* promoter drives constitutive expression of a tetracycline repressor, TetR. TetR binds to and represses the promoter of the plant viral cauliflower mosaic virus 35S (CaMV35S) carrying three Tet operators (binding site for TetR). Addition of tetracycline inducer (yellow stars) inactivates TetR and thus activates I-*Ppo*I expression. **(B)** Diagram showing the I-*PpoI* recognition sequence, cleavage site within rDNA and typical sequence change in cells resistant to I-*Ppo*I cleavage (ARS, autonomously replicating sequence). **(C)** Analysis of DSB induction by I-*Ppo*I and resection by Southern blot, and quantification of kinetics of resection at DSB ends and at 3.2 kb from the DSB. Diagram shows I-*Ppo*I cleavage site at the *lys1* locus. **(D)** Diagram of the SSA assay between two partial lambda sequences. Southern blot analysis of SSA. Lambda sequence was used as a probe.

Here we describe DSB-induced recombination assays in fission yeast, a model organism that offers some advantages when compared to budding yeast. Initial DSB inducible recombination assays with HO endonuclease induction under a thiamine-regulated *nmt* promoter, uracil-regulated *urg1* promoter [195], or glucose-repressed *inv1* promoter were inefficient in terms of synchrony of DSB induction when compared to budding yeast assays. An additional complication of these assays is that induction of the DSB requires a switch of growth media and thus alteration of cellular metabolism that could impact DSB repair analysis. Recently, the Sanders lab developed an assay where the homing endonuclease I-*Ppo*I from the slime mold *Physarum polycephalum* is placed under the control of an anhydrotetracycline (ahTET)-inducible promoter [196]. Addition of anhydrotetracycline to the growth medium inactivates the Tet repressor leading to high promoter activity (**Figure 10A**).

The drawback of I-*Ppo*I enzyme usage is that its recognition sequence lies within rDNA repeats (**Figure 10B**). While cleavage within rDNA can be used to follow DSB repair within repetitive sequences [197], in most studies it is undesirable feature. To acquire cells resistant to I-*Ppo*I enzyme cleavage within rDNA, cells are plated on anhydrotetracycline containing plates. A small number of cells (∼1/1000) survive rDNA cleavage via a typical single nucleotide change within the I-*Ppo*I cleavage site [198]. Sequencing of this region is then essential to confirm that the rDNA carries the mutation within the I-*Ppo*I cleavage sites (**Figure 10B**). To avoid rDNA cleavage, one can also use I-*Sce*I enzyme instead of I-*Ppo*I. Such an assay was recently developed by the Runge lab with codon-optimized I-*Sce*I endonuclease carrying two nuclear localization signals (NLS) at the N-terminus. In this new system I-*Sce*I is also under the control of an anhydrotetracycline (ahTET)-inducible promoter and cleavage by I-*Sce*I is nearly as efficient as with I-*Ppo*I enzyme [199].

An example of the synchronous and efficient induction of a DSB by I-*Ppo*I and analysis of DSB ends resection are shown in **Figures 10 C-D**. In this assay, the TetO7::I-*Ppo*I cassette is inserted at the *leu1* locus and a single DSB is generated at the *lys1* locus as described [196]. DSB induction and resection are followed using Southern blots and probes specific for the cleavage site or 3.2 kb away from the DSB ends. We note that induction of DSBs is faster in EMMG media when compared to rich media and best at low cell density (2-4 x 10^6^/ml). Cells are collected before DSB induction and every 30 minutes after induction. DNA is isolated by standard phenol–chloroform extraction, digested with *Eco*RI and separated on 0.8% agarose gel, then transferred to a positively charged membrane and subjected to Southern blot analysis with probes corresponding to sequences at the DSBs. The kinetics of resection directly correlates with the kinetics of disappearance of Southern blot bands corresponding to the probes used.

We also note that the strain described above cannot be used efficiently to study NHEJ associated with errors. Cells plated on tetracycline containing plates induce I-*Ppo*I enzyme permanently and normally only cells that repair the DSB by an NHEJ event that alters the I-*Ppo*I cleavage site should be able to survive. Here, however, TetO7::I-*Ppo*I was inserted at the *leu1* locus with a pDUAL plasmid that generates two direct *leu1* gene fragments [200]. Recombination between two *leu1* fragments leads to a loss of the I-*Ppo*I cassette and therefore growth on tetracycline plates. The frequency of this spontaneous event is within the range of error associated with NHEJ and therefore the system is not best suited to study error-prone NHEJ (Ira's lab unpublished results).

Another drawback of this system is that a fraction of cells induce I-*Ppo*I and DSBs without the addition of tetracycline, which makes establishing a repairable system difficult. A similar problem was observed with repairable recombination assays using HO under the control of the *urg1*promoter (e.g. [201]). To avoid such issues, a repairable system where the repair of a DSB by SSA leads to the elimination of an essential gene, *gtr2* and consequently cell death was designed (**Figure 10C**). Thus, cells in which a DSB is induced prior to tetracycline addition are eliminated from the population. The system presented in **Figure 10D** is ideal to compare the efficiency and kinetics of DSB repair by SSA in fission yeast. A single DSB is induced at an I-*Ppo*I cleavage site inserted within a partial sequence of phage lambda integrated at the *arg1* locus on chromosome III. It is repaired by SSA using an identical lambda sequence (755 bp) located 21 kb upstream. To eliminate Rad51-mediated strand invasion, the analysis is performed in *rad51Δ* cells.

An alternative way to limit background expression of I-*Ppo*I could be accomplished by incorporation of a DSR sequence (Determinant of Selective Removal) at the 3′ UTR of the I-*Ppo*I gene that directs background transcripts through the MTREC complex to the nuclear exosome for degradation [197]. This recently described system of I-*Ppo*I induction is similar to the one presented in **Figure 10A**, with the endonuclease under the control of the anhydrotetracycline (ahTET)-inducible promoter. However, this alternative scenario uses a modified *CYC1* promoter coupled with seven Tet operator sequences (tetO7) repressed by constitutively low expression of TetR (TetR-tup11). The same TetO7-*CYC1* promoter also controls expression of ncRNA-8xDSR. Addition of ahTET to the media releases TetR from the promoters of I-*Ppo*I-4xDSR and 8xDSR and high amounts of ncRNA-8xDSR compete for MTREC pathway components, thus stabilizing the I-*Ppo*I-4xDSR nuclease. Rapid DSB formation within rDNA or within a separately inserted I-*Ppo*I recognition site is observed in this system [197].

As presented here, a number of new DSB-inducible recombination assays were developed recently in fission yeast. These assays will facilitate studies of DSB-induced recombination in fission yeast.

#### Quantification of strand-specific single-stranded DNA in DNA repair by real time PCR

Single-stranded DNA (ssDNA) is an obligatory intermediate in DNA replication and transcription events and naturally forms at telomeres through unwinding of duplex DNA by DNA helicases or by nucleolytic degradation of one strand [202]. ssDNA is also generated in all types of DNA repair processes including nucleotide excision repair (NER), base excision repair (BER) and DSB repair (DSBR). Moreover, ssDNA gaps arise as a part of DNA damage tolerance when DNA lesions impede progression of replication forks. To detect and measure ssDNA intermediates and their strand-specificity within cells would be essential to monitor progression of all these events and to understand the underlying molecular mechanisms. Traditional methods to detect intracellular ssDNA typically involve Southern hybridization or immunoblotting which are time-consuming and require a substantial amount of materials and reagents. Here we describe a convenient, quantitative and highly sensitive way to detect strand-specific ssDNA in the genomic DNA in *S. cerevisiae* cells. This method could be readily adapted to other organisms.

**Figure 11:**
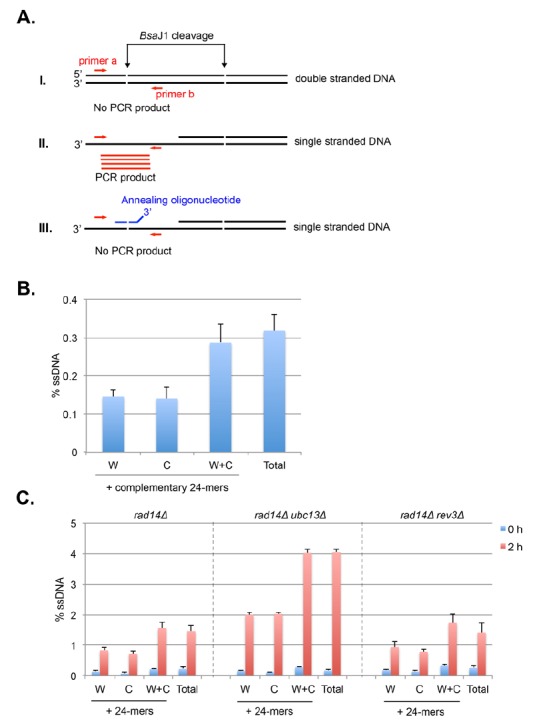
FIGURE 11: Schematic principles and determination of strand-specific ssDNA detection by restriction enzyme digestion and real-time PCR. **(A)** Detection of strand-specific ssDNA by real time PCR. **i.** Duplex DNA molecule is digested by *Bsa*J1 restriction enzyme. No PCR product will be generated using primers (a and b, red arrows) flanking the restriction enzyme recognition site; **ii**. ssDNA is resistant to enzyme cleavage and permits the formation of PCR products (red lines) flanking the restriction site using a set of primers (red arrows); **iii.** Addition of complementary oligonucleotides with 4 nucleotide mismatches at the 3′ end (blue broken line) renders ssDNA cleaved by *Bsa*J1 and suppresses the formation of PCR products. **(B)** The level of total and strand-specific ssDNA at 2.1-kb right distal to the origin *ARS305* in the genomic DNA isolated from G1-arrested *RAD14*^+^ cells. 24-mer complementary oligonucleotides to Watson or Crick strands were added to determine strand-specificity of ssDNA. Percent ssDNA (% ssDNA) is calculated using the formula: %ssDNA= 200×2^Δct^ / (1+2^Δct^) % wherein Δct = ct_control_-ct_*Bsa*J1_+PE_adj_. ct_*Bsa*J1_ is the ct value at the *Bsa*J1 site that is 2.1 kb distal to *ARS305*. ct_control_ is the ct value at the locus 0.9-kb right distal to *ARS305*, a control locus that does not contain a *Bsa*J1 restriction site; PE_adj_ is a constant value adjusting the PCR efficiency difference between the primer sets for 0.9-kb and 2.1-kb, which equals ct_2.1k_-ct_0.9k_ using undigested genomic DNA as the PCR template. The results are the average of three independent experiments + s.d.; **(C)** The level of total and strand-specific ssDNA at 2.1-kb right distal to the origin *ARS305* at G1 and 2 hours post G1 release after 10 J/m^2^ UV treatment in *rad14Δ, rad14Δ ubc13Δ,* and *rad14Δ rev3Δ* cells. 24-mer complementary oligonucleotides to Watson (W) or Crick (C) strands were added to determine strand specificity of ssDNA. Percent ssDNA (%ssDNA) is calculated as described in B above. The results are the average of three independent experiments + s.d.

In principle, restriction endonucleases can only digest duplex DNA molecules while leaving ssDNA intact. By exploiting this differential enzyme digestion pattern, this strategy quantifies the percentage of ssDNA at a specific locus in the genomic DNA by real-time PCR. To discriminate strand-specificity of the ssDNA generated, i.e. the Watson or the Crick strand, a 24-mer oligonucleotide complementary to the restriction enzyme recognition site on either the Watson or Crick strand is included in the reaction. Then based on the enzyme digestion and PCR patterns we can deduce the strand of origin. For instance, if the ssDNA corresponds to the Watson strand, inclusion of complementary oligonucleotides will render it cleaved by restriction enzyme and ssDNA from the Watson strand will no longer be amplified by PCR using primers flanking the restriction site (**Figure 11A**). To avoid excess levels of annealing oligonucleotides being used as primers to generate PCR products in the real-time PCR reaction, four mismatched nucleotides are placed at the 3′ end of the oligos, effectively blocking their contribution as PCR primers.

Several labs have used this or similar strategies [203–205] to measure the formation of ssDNA at a site-specific HO-induced DSB, a process called DNA end resection, during which both ends from a DSB undergo 5′ to 3′ degradation by a group of nucleases, generating ssDNA with 3′-OH overhangs [206]. DNA end resection is a key step in the initiation and regulation of DNA recombination and the DNA damage response in all eukaryotic cells [202]. Compared to Southern blot hybridization, the PCR based assay is fast and requires fewer cells to reliably detect ssDNA at given locations. Furthermore, from a single digestion reaction, it is possible to monitor the ssDNA formation at multiple locations in the genome and deduce the rate of resection based on the progressive formation of ssDNA at several distal locations. One routinely used restriction enzyme is the *Bsa*J1 which recognizes C^CNNG_G sequence as a frequent cutter. Previously, the Diffley lab used *Bst*U1(CG_^CG) for the equivalent PCR based assay [204]. Since *Bsa*J1 cuts at 60°C, the annealing and enzyme digestion can be performed as a single step to assess the strand specificity of ssDNA.

The sensitivity of this PCR based ssDNA detection assay depends on the efficacy of the enzyme digestion because residual DNA that remains undigested in the reaction can increase background signals and thus limit the sensitivity of the assay. It was found that *Bsa*J1 is very efficient in restriction digestion and well suited for the assay. Using the genomic DNA samples isolated from G1 arrested wild-type cells, only 0.1-0.4% background levels are detected of PCR signal at any given location (**Figure 11B**). Notably, the sum of ssDNA from Watson and Crick strands as determined by annealing of corresponding oligonucleotides is consistently close to that without adding annealing oligonucleotides, with the ratio of ssDNA from Watson and Crick strands corresponding to almost one. The result suggests that the 0.1-0.4% background levels of PCR are not derived from undigested DNA but from actual ssDNA which is likely generated spontaneously either *in vivo* or during genomic DNA isolation. The result also suggests that the complementary annealing of oligonucleotides and subsequent enzyme digestion are very efficient.

In addition to analyzing resection at DNA breaks, this technique was also used to determine the levels and the strand specificity of ssDNA in cells suffering from replication stress to study the roles of DNA damage tolerance (DDT) mechanisms in post-replicative gap repair in budding yeast cells. Multiple types of lesions on DNA templates can stall the ongoing DNA synthesis activity of high fidelity DNA polymerases, and the lesions must be tolerated/bypassed to ensure that replication is completed in time [207]. Evidence emerges that DNA synthesis can restart downstream of a lesion on the leading strand template by re-priming and a ssDNA gap is left behind. Similarly, on the lagging strand template, a ssDNA gap could be formed due to an interruption of Okazaki fragment synthesis when the lagging strand polymerase encounters a blocking lesion [207]. The resulting ssDNA gaps are then filled in by Rad18-dependent DDT mechanisms in a post-replicative manner. The DDT pathways can be further divided into two branches, error-prone translesion synthesis (TLS), and Rad5, Ubc13-dependent template switching [208, 209]. Importantly, ssDNA is the common substrate of both DDT pathways. To understand DDT mechanisms, ssDNA formation in S phase from cells exposed to UV lesions was examined using a PCR based assay and this allowed to analyze the role of Rev3 or Ubc13 in the gap repair. Briefly, NER-deficient *rad14Δ* cells were arrested in G1 phase, treated with 10 J/m^2^ UV and released cells into S phase. Then the formation of ssDNA was measured at multiple locations near the early firing ARS305 replication origin (**Figure 11C**). By determining strand specificity of ssDNA, it was also examined whether ssDNA accumulates from the leading or lagging strand (**Figure 11C**). As expected, the sum of ssDNA at leading and lagging strands is nearly identical to the total ssDNA detected, again reinforcing that the complementary annealing of excess oligonucleotides and enzyme digestion are very efficient in this assay. The results suggest that Ubc13 suppresses the formation of ssDNA in both leading and lagging strands in S phase (**Figure 11C**).

Despite many advantages of the PCR based ssDNA detection assay described herein, the method could underestimate the amount of intracellular ssDNA at a given locus if ssDNA formation relies on the temporary unwinding of duplex DNA such as that formed during undisturbed DNA replication or transcription. Unwound DNA will simply re-anneal during genomic DNA isolation and is still cleaved by restriction endonuclease. It was estimated that the lower limit of detection in this PCR assay is ∼0.1% due to the spontaneous formation of ssDNA in the genomic DNA isolated from G1-arrested cells. Levels of ssDNA below this threshold will not be detected by the current technique. The use of 20-24-mer oligonucleotides also limits determination of the strand specificity of ssDNA less than 20 nucleotides long.

In summary, we have presented here a fast, sensitive and cost-effective assay to measure the amount of ssDNA at defined locations in budding yeast cells. This technique can also determine the strand specificity (either Watson or Crick strand) of the ssDNA. Since ssDNA formation is a common feature in many DNA transactions, this assay should have wider applications and could also be applicable to analyzing the equivalent processes in other organisms including human cells.

#### Detection of toxic recombination intermediates in the yeast S. cerevisiae

ssDNA is a recombination intermediate that promotes homology search and strand invasion. However, unregulated accumulation of excessive ssDNA can also lead to the formation of structures containing both the broken and intact DNA molecules, called toxic intermediates, which if not resolved are detrimental for the cell. Accumulation of toxic recombination intermediates leading to cell lethality have been observed in various yeast mutant backgrounds, including *sgs1*Δ*, top3*Δ, and *srs2*Δ [39, 210–213], which suggested that these genes play an important role in preventing accumulation of toxic intermediates that tether recombining DNA molecules together. Recently, experiments performed in *srs2*Δ yeast strains, where a DSB induced by a site-specific HO-endonuclease was repaired by BIR allowed both visualization and subsequent investigation of toxic recombination intermediates [214]. Similar to other homologous recombination pathways, BIR is initiated by 5′-3′ resection of a DSB end which then invades into the homologous template and begins synthesis that can copy >100kb of the template until the end of the chromosome [216, 217]. BIR proceeds by a migrating bubble where asynchrony between leading and lagging strand synthesis leads to accumulation of long regions of ssDNA [218]. These regions are stabilized by RPA, a ssDNA binding protein, along with Rad51, a strand exchange protein [219]. These protein-bound ssDNA located behind of BIR bubble can undergo unscheduled pairing with the template, which forms joint molecules that if not resolved become toxic recombination intermediates, joint molecules. It was demonstrated that accumulation of toxic joint molecules is counteracted by Srs2 creating the opportunity to employ BIR experimental systems in *srs2*Δ mutants to investigate genetics, kinetics of accumulation and disassembly, and the structure of toxic recombination intermediates.

**Figure 12:**
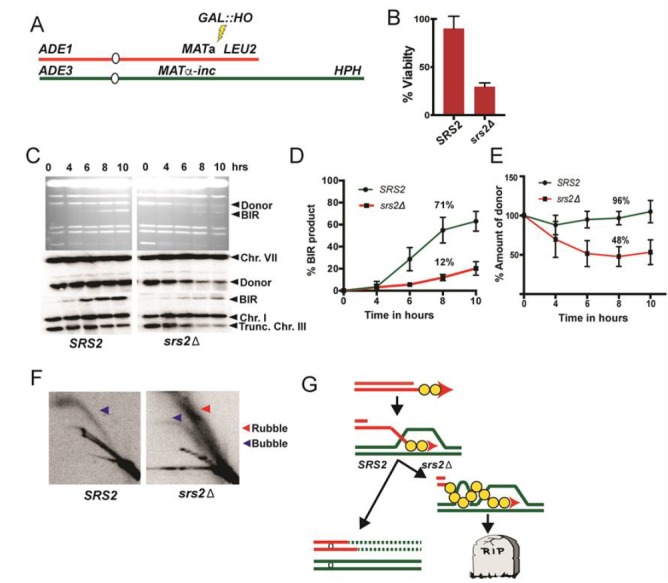
FIGURE 12: Detection of toxic recombination intermediates during BIR in yeast. (from data in Elango et al [214] published under open access under a Creative Commons Attribution 4.0 International License) **(A)** Experimental system to study BIR. BIR is initiated by DSB introduced by galactose-induced HO endonuclease at *MAT***a** locus in yeast disomic for Chr. III **(B)** Cell viability following DSB induction (%). **(C)** BIR kinetics analyzed by CHEF gel using cells taken at indicated time points following DSB induction. Upper panel: CHEF gels stained with Ethidium Bromide. Subsequent panels below show Southern blot analysis using *ADE1*-specific, and *ADE3*-specific probes, hybridizing to the recipient and the donor chromosomes, respectively. **(D)** Quantification of BIR product. **(E)** Quantification of donor chromosome entering the gel. **(F)** 2D gel analysis of BIR intermediates in SRS2 and *srs2*Δ at 7h following DSB induction. Genomic DNA was digested with BglII to detect intermediates at 24 kb position. Intermediates were detected using a probe specific to the 24 kb position of Chr III. Blue arrowheads denote bubble arc intermediates and red arrowhead denotes ‘rubble' structure. **(G)** The schematics shows broken recipient chromosome (red) invading unbroken homologous donor (green). Repair DNA synthesis is initiated and progresses by a migrating bubble. In *SRS2:* successful completion of BIR with conservative inheritance of newly synthesized DNA. In *srs2*Δ: formation of toxic joint molecules via unscheduled invasion of ssDNA located behind the BIR bubble into homologous chromosome leading to cell death.

The key features of the BIR experimental system (**Figure 12A**) used in these experiments [216] are:

1) A yeast *S. cerevisiae* strain that is disomic for chromosome III containing one full and one truncated copy of chromosome III. The truncated copy was created via replacement of the telomere proximal to the *MAT***a** region by *LEU2* followed by a telomere sequence.

2) A galactose inducible DSB that is initiated at the *MAT***a** locus of the truncated copy of Chr III, while the full-length chromosome is refractory to cutting because it contains an uncleavable *MATα-inc* allele. Following the DSB, the broken truncated copy of Chr III invades into the full-length copy and initiates BIR that proceeds for approximately 100 kb. BIR is the predominant mechanism of repair in this system because almost all homology between the recipient and the donor is eliminated centromere distal to *MAT* [216].

Formation of toxic recombination intermediates during BIR in this system is assessed in *srs2*Δ strains and can be followed by several different analyses, including genetic (viability) test, by measuring kinetics of DSB repair by contour-clamped homogenous electric field (CHEF) gel electrophoresis, two-dimension gel electrophoresis and electron microscopy. The first and second methods are the most straightforward and will be described here in more details.

To test cell viability following BIR induction, *SRS2* and *srs2*Δ cells are pre-grown in YEP-lactate media (not containing glucose, which is important for the efficient uptake and DSB induction by galactose) and then serially diluted and plated at an approximate concentration of 50 to 100 cells per plate on YEP media supplemented with 2% galactose (where DSBs are initiated and repaired) as well as on YEPD media (as a No-DSB control.) Following 3 to 5 days of incubation at 30^0^ C, the yeast colonies are counted and the viability is calculated by normalizing the number of colony-forming units (CFUs) on YEP-Gal by the CFU on YEPD. A minimum of three plating experiments are performed to calculate the average viability and standard deviation. Following initiation of BIR, the viability in *srs2*Δ was approximately 30%, which was significantly lower as compared to wild type cells (∼80%) (**Figure 12B**) [214]. Such massive death detected in *srs2*Δ mutants was unusual for this experimental system where the presence of two copies of Chr III, one of which remains unbroken, allows the cells to survive even in situations when the second, broken chromosome is left unrepaired and lost [216]. This loss of viability in *srs2*Δ is indicative of the persistence of joint molecules that trap the donor and recipient molecules together.

Toxic intermediates are formed during BIR and can be detected by examining the kinetics of BIR which can be followed by separating BIR repair products from other chromosomes using CHEF gel electrophoresis. Using this method, the BIR repair product (300 kb) can be easily distinguished from the original (truncated) Chr III that, before HO-induced DSB, is approximately 200-kb-long (**Figure 12C**) [216, 218]. BIR repair time-course is performed by collecting 50 mL samples for CHEF before and after DSB induction. BIR typically takes 4 h for initiation and 5–10 hrs for completion. Therefore, time points are collected every 2 h following BIR induction for a total of 8–10 hrs. Cells collected at each time point are spun down and embedded in 1% low melting agarose plugs after treatment with zymolyase. The resulting DNA plugs are then used for CHEF gel electrophoresis using the Bio-Rad-CHEF-DRII or other similar CHEF machines. When this experiment was performed in *srs2*Δ and *SRS2* strains*,* it was observed that the amount of BIR product (visualized by hybridization of the recipient chromosome with radioactively labeled *ADE1* probe) measured 8 h after DSB induction was nearly five-fold less abundant as compared to wild-type (**Figure 12C, D**) [214]. In addition, while the amount of template (donor) Chr. III molecule (visualized by hybridization with radioactively labeled *ADE3* probe) in wild-type cells remained constant throughout the course of BIR, in *srs2*Δ it drastically decreased (**Figure 12C, E**). At 8 h, the amount of the donor entering the gel was only 48% of the initial amount before DSB induction in *srs2*Δ as compared to 96% in *SRS2*. This decrease of donor molecules in the agarose gel in *srs2*Δ is indicative of the accumulation recombination intermediates as branched DNA structures. These toxic branched structures can be further visualized by using two-dimensional (2D) gel electrophoresis of BglII or KpnI restriction enzyme digested genomic DNA obtained from *SRS2* and *srs2*Δ cells undergoing BIR. Following 2D gel electrophoresis, DNA is transferred to a nylon membrane and is hybridized with radioactively labeled probes specific to various positions centromere-distal to the DSB located on Chr III (**Figure 12F**). Previously, this method was used to demonstrate that BIR is carried out by a migrating bubble, and ssDNA accumulates due to asynchronous synthesis of leading and lagging strands [218]. In *srs2*Δ, the bubble intermediate is barely detectable (**Figure 12F**) while another BIR intermediate becomes more prominent. This intermediate called “rubble” (**Figure 12F**) consists of heterogeneous DNA molecules resulting from toxic recombination that are more branched and heavier than those forming the bubble intermediate (see schematics in **Figure 12G**) [214]. The structure of individual molecules containing toxic intermediates can be further examined by electron microscopy, which allowed to determine that these toxic intermediates represent 3- and 4-way junctions formed by the unscheduled invasion of ssDNA accumulated during BIR and are positioned behind the BIR bubble [214].

When investigating the formation of toxic intermediates by CHEF, these branched intermediates do not migrate inside the gel and remain in the well. They can be visualized in the well by hybridization with *ADE1*-specific probe. However, since branched DNA from the well is diffi-cult for the transfer to the membrane, its quantification is complicated. In addition, accumulation of other branched intermediates takes place even in S-phase, and also even in *SRS2* cells, which further complicates its quantification. The transfer of branched DNA intermediates can be facilitated by additional UV Irradiation of the wells before transfer to the membrane. It should also be noted that the reduction of the amount of donor DNA entering the gel due to formation of branched intermediates might be difficult to detect later in the time-course (after 6-8 h following HO induction) because cells that successfully complete DSB repair (by BIR or gene conversion) continue to divide and eventually outcompete cells that stay arrested in their cell cycle due to accumulation of toxic repair intermediates. Also, toxic intermediates are likely formed in *srs2*Δ (and possibly in some other mutants) during other recombination events [214]. However, it might be more difficult to detect them due to smaller chromosomal regions affected by these intermediates. 2D analysis of toxic recombination intermediates allows their visualization in a large cell population but does not allow analysis of the intricate details of their structure. Analysis of toxic intermediates by electron microscopy might provide a better resolution. However, identifying intermediates affecting only one of 17 chromosomes is difficult and tedious. A more detailed analysis of toxic recombination intermediates will require enrichment for the region of Chr III involved in BIR, which can be achieved by: (i) inserting a LacO array into the relevant region of chromosome III, (ii) performing a restriction digest with rare-cutting endonucleases, and (iii) conducting pulldown using immobilized LacR protein that recognizes and binds to the LacO sequence with high affinity [220].

Overall, the methods of analysis presented here allow for the detection of toxic recombination intermediates formed during BIR in *srs2*Δ mutants. In the future, the same methods could be applied to the detection of such intermediates formed in other mutants, as well as in the context of other pathways involving recombination.

### Detection of hypermutable single-strand DNA formed in living yeast cells

**Figure 13:**
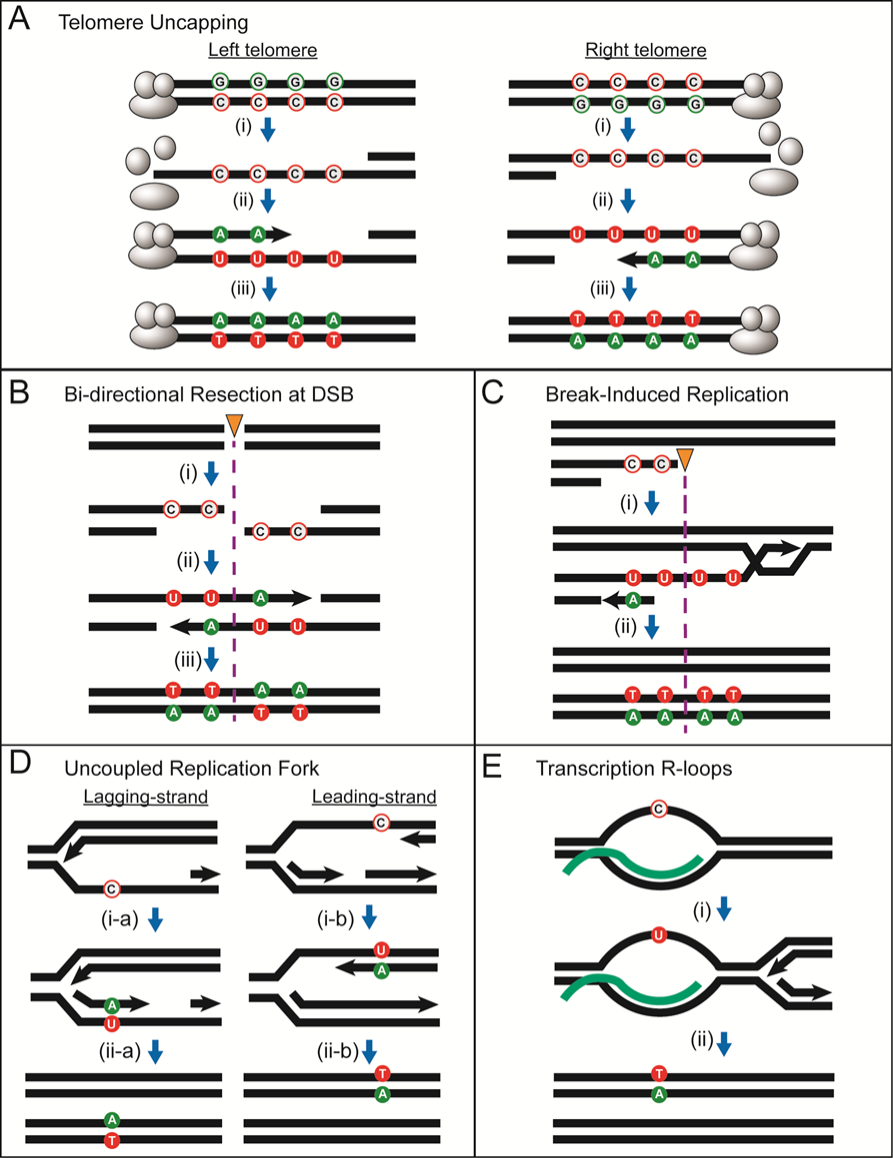
FIGURE 13: Cellular processes generating transient ssDNA vulnerable to hypermutation by APOBEC. The following symbols in the figure include: “unfilled green circles” = non-mutated guanines; “unfilled red circles” = non-mutated cytosines; “green-filled circles” = mutated guanines; “red-filled circles” = mutated cytosines; “black arrow heads” = 5′ to 3′ DNA synthesis; “orange triangle with purple dotted line” = position of double-strand break; “C” = cytosine; “A” = adenine; “U” = uracil; “T” = thymine. In all models, C to U changes are due to APOBEC cytosine deamination. **(A)** Long ssDNA generated at uncapped telomeres. (i) telomere uncapping triggers 5′ to 3′ end resection and G2 cell cycle arrest, generating long stretches of persistent ssDNA that provides a substrate for deamination of cytosines to uracils by APOBEC. (ii) the capping of telomeres is reestablished, initiating restoration of dsDNA with adenines inserted in the nascent DNA opposite the uracils. (iii) after the next round of replication, mutations are fixed, resulting in C:G to T:A transitions. Deamination of cytosines in the left telomeres result in G-coordinated clustered mutations on the top strand, while deamination of cytosines in the right telomeres result in C-coordinated clustered mutations on the top strand. **(B)** ssDNA formed from bi-directional resection at a double-strand break (DSB). (i) 5′ to 3′ end resection on both sides of a double strand break result in ssDNA in the top strand on the left-side of break and in ssDNA in the bottom strand on the right-side of the break. Cytosines in ssDNA on both sides of the break will be deaminated by APOBEC. (ii) and (iii) restoration of dsDNA using a sister chromatid or a homologous chromosome template followed by a round of replication will generate a single switch in strand coordination from C to G in a 5′→3′ direction of the top strand. **(C)** ssDNA formed from the repair of a one-ended DNA break via BIR. (i) 5′ to 3′ end resection results in a 3′ overhang that invades a homologous template and initiates DNA synthesis that progresses via a migrating replication bubble that generates a long ssDNA tail behind the bubble. Asynchronous lagging strand synthesis initiates to restore DNA to double strand form resulting in C-coordinated clustered mutations. If BIR initiated repair from the opposite side of a break such that ssDNA was generated on the bottom strand, then this would result in a G-coordinated cluster. **(D)** Long ssDNA could form from uncoupling between replicating leading and lagging strands. (i-a) and (ii-a) stalling of lagging strand synthesis resulting in ssDNA formed on the bottom strand leading to a mutated guanine. (i-b) and (ii-b) stalling of leading strand synthesis resulting in ssDNA formed in the top strand leading to a mutated cytosine. If uncoupling leads to long stretches of ssDNA, coordinated cluster formation is possible. In both cases, the origin of replication (not shown) is on the left side. The polarity of mutated cytosines or guanines depends on the fork direction. **(E)** Small stretches of ssDNA in the non-transcribed strand at transcription R-loops. (i) and (ii) ssDNA in the non-transcribed strand results in genome-wide bias towards APOBEC-mutated cytosines in non-transcribed strands. If uncoupling leads to long stretches of ssDNA, coordinated cluster formation is possible.

Resistance to DNA damage is in part assured by the double-stranded (ds) structure of DNA, which protects atoms of nitrogenous bases participating in hydrogen bonding. Moreover, the genomes of living cells can repair many thousands of simultaneously occurring DNA lesions without a trace, in many cases, due to the dsDNA structure, which provides an intact template for excision repair pathways. On the contrary, lesions in transient single-stranded (ss) intermediates of various DNA transactions (**Figure 13**) can easily be a source of mutations through error prone replication of the damaged strand. It was found in yeast that many kilobases of ssDNA formed by resection at DSBs or at uncapped telomeres, as well as by BIR, can be efficiently restored to dsDNA, even if the ssDNA contained dozens of lesions induced by ultraviolet light (UV), methyl methanesulfonate (MMS), chemical cytosine deamination by sulfites or endogenously induced abasic (AP) sites [221–225]. The recovery was assured by highly efficient TLS. Because TLS is error prone, many lesions in the transient ssDNA region were converted into closely spaced mutations (mutation clusters). Mutation density in a cluster exceed 1,000 - 10,000-fold overall mutation density in a genome, where lesions rarely occurred or were efficiently removed by error free NER or BER systems. A related source of lesions in a single DNA strand resulted in similar mutation types within that strand (strand-coordination). For example, after UV exposure, mostly pyrimidine bases were mutated in a single strand [221, 225]. Strand-coordinated clustering of mutations found in yeast prompted search for a similar phenomenon in genomes of human tumors, where such a clustering pattern was found in abundance in many cancer types [223, 226–230]. The most prominent cause of strand-coordinated clusters was identified as ssDNA specific APOBEC cytidine deaminases removing an amino group from cytosine which converts it to an uracil base. APOBECs are a part of the innate immunity system attacking retroviral and retrotransposon ssDNA intermediates. When these enzymes gain access to chromosomes they leave a trace of mutations, as long as the transient ssDNA persisted long enough to incur damage [223, 228, 231]. The exquisite specificity of APOBECs to deaminate cytosines result in exclusively mutated cytosines (or guanines) in the top strand reported in sequence data. Such C- or G-coordinated clusters could extend to many kilobases and contain dozens of mutations, all in C (or in G) nucleotides [223, 227, 229, 232]. The incidence of APOBEC-induced clusters indicated that long stretches of hypermutable ssDNA occur in human cancers and possibly in other types of cells and organisms. APOBEC enzymes were then used in yeast by several groups to explore the formation and genome-wide distribution of this unusual hypermutation substrate; long, persistent ssDNA.

By now, several different APOBEC enzymes expressed in yeast were used for this purpose: human APOBEC3A, APOBEC3B, APOBEC3G and the lamprey APOBEC-like PmCDA1 deaminase [222, 227, 233–237]. In these experiments, APOBEC ORFs are usually expressed from a strong promoter, such as Gal1-10 or Tet. In wild type yeast, uracils formed by APOBEC cytidine deamination rapidly turn into AP sites by yeast uracil DNA glycosylase (Ung1). AP sites in a ssDNA template are copied by error prone TLS polymerases placing either an A or G across an AP site, thereby resulting in C→T or C→G mutations, respectively [238, 239]. AP sites can also break spontaneously or enzymatically, preventing the detection of long ssDNA based on mutagenesis. Thus, in order to improve detection of ssDNA stretches by APOBEC-induced cytosine deamination, *UNG1* ORF can be deleted. During restoration to dsDNA, uracils in the template do not impede DNA polymerases and do not cause DNA breakage. Instead, all uracils are accurately copied with insertions of adenines resulting in C→T mutations. Also, since *UNG1* defect blocks BER, mutations are generated even when there was a chance of BER to correct the damage using a second intact strand as a template (**Figure 13D, E**). Mutation detection is performed in isolates with selectable mutation reporters and/or by sequencing the entire yeast genome. This approach provided a useful tool for detecting different types of hypermutable ssDNA substrates described below.

#### Uncapped telomeres (***Figure 13A***)

Long ssDNA is formed at uncapped telomeres because they are recognized similar to the ends of DSBs and processed by 5′→3′ end resection proteins [240]. In these studies, the telomere uncapping and the generation of long ssDNA overhangs were triggered by shifting temperature-sensitive yeast with a *cdc13-1* defect in the telomere capping protein to a non-permissive temperature of 37°C [221, 222, 225, 227]. Uncapping of telomeres triggered 5′→3′ end resection and G2 cell cycle arrest. Cells can be kept in non-permissive temperature for 6 hours in rich medium or for as long as 48 h in buffer, which allows sufficient time for APOBEC to deaminate multiple cytosines in the resulting stretch of ssDNA. Telomere capping is then restored when cells are moved back to a permissive temperature of 23°C, followed by the restoration of dsDNA with adenines inserted opposite the uracils independent of a TLS system. Mutation reporters (different combinations of *LYS2, CAN1, URA3* and *ADE2* ORFs) placed in the vicinity of the left telomere of chromosome V showed over a 100-fold increase in mutagenesis as compared to the same reporter placed in the middle of chromosome II, where end resection could not reach [222, 227]. Sequencing of the reporter region followed by whole genome sequencing revealed strand biased C-coordinated (in right telomeres) or G-coordinated (in left telomeres) clustered mutagenesis consistent with long, up to ∼ 40 kb, 3′-overhangs generated by 5′→3′ resection followed by multiple (up to 36) cytosine deaminations and resulting in C→T (or G→A) mutations. The median density of mutations in a cluster was about 1.4 mutations/kb, indicating the size limitation for detection of a single stretch of persistent long ssDNA. Usually, clustered mutations were at several telomeres of a sequenced genome in addition to a cluster in the region of the selected reporter. So far, this approach has been applied only to *cdc13-1* mutant yeast. In the future, it may become useful to detect problems with telomere capping in a variety of mutant strains carrying candidate genetic defects.

#### Long bi-directional resection at DSBs (***Figure 13B***)

Efficient recombinational repair of DSBs in yeast can occur with relatively short, as little as 150 bp, resected regions [189, 241]. However, some breaks can undergo many kilobases of resection in the absence of a homologue and still be repairable with an oligonucleotide introduced into the cell by transformation [242]. Long bi-directional resection followed by repair using a sister chromatid was also suggested to explain the pattern of MMS-induced mutation clusters in yeast [223]. Hypermutable ssDNA formed by long bi-directional resection can be identified by switching the strand coordination pattern of mutation clusters. A single switch in strand coordination from C to G in a 5′→3′ direction of the top strand (the strand reported in sequence data) is expected, because 5′→3′ resection leaves single stranded DNA on the top strand to the left of the break and on the bottom strand to the right of the break. In the opposite case of single switch coordination where G is followed by C, and/or in cases of multiple switches in strand coordination, bidirectional resection alone cannot explain these cluster patterns, and therefore they may be assigned to events involving more than a single break at the incidence of the cluster or to a cascade of breakage in the course of repair of a single initial break [243]. It is important to note that resection is not necessarily symmetric around a double-strand break [244]. If a DSB with long asymmetrical resection is repaired it could generate hypermutable ssDNA on only one side of a DSB which could result in completely C- or G- coordinated clusters similar to outcomes of BIR described below.

#### Break-induced replication (**Figure 13C**)

When only a single side of a break participates in repair by BIR, long hypermutable ssDNA is generated behind a BIR replication-like bubble upon initiation of DNA synthesis [218, 224]. Importantly, BIR would generate a stretch of ssDNA in the same strand as long asymmetrical resection at the end of the break involved in BIR, thereby resulting in identical patterns of strand coordinated cluster. Non-switching, completely C- or G-coordinated clusters were observed in yeast grown in the presence of APOBEC expression [233, 236]. A similar type of non-switching strand coordination conforming to the mutagenic specificity of MMS in ssDNA were reported in yeast growing in the presence of MMS or in association with BIR events that were triggered by a site-specific DSB repaired in the presence of MMS [224].

#### Replication forks (**Figure 13D**)

Long stretches of ssDNA are either rare or do not form in normal replication forks, however ssDNA specific mutagenesis can be detected by whole genome sequencing of yeast grown in the presence of APOBEC by preference of mutated cytosines over guanines in the lagging strand template [233]. This preference should be even more evident for mutations conforming to a known signature of APOBEC enzyme used in the experiment [245]. Hypothetical long-range uncoupling between copying leading and lagging strands in the presence of APOBEC expression would result in completely C- or G-coordinated clusters, depending on the strand delayed for copying as well as on fork direction. This pattern is the same as expected for one-end resection and/or BIR (**Figure 13C**)**.** The orientation of clusters in relation to known replication origins, and the involvement of known replication defects would reveal uncoupling as an underlying mechanism for coordinated cluster formation.

#### Transcription R-loops (**Figure 13E**)

Because of the relatively small size of ssDNA formed in transcription associated R-loops, APOBEC mutagenesis is not expected to generate mutation clusters. Similar to replication forks, APOBEC mutagenesis in R-loops can be detected by a preference of mutations in cytosines over guanines in whole-genome sequenced APOBEC-mutagenized yeast, but in this case, preference should be for mutations in the non-transcribed versus the transcribed strand. APOBEC mutagenesis and the formation of mutation clusters biased towards non-transcribed strand was increased by inactivation of transcription initiation factor Sub1 [234]. APOBEC3B mutagenesis turned to be extremely efficient in non-transcribed strand of yeast tRNA genes [246]. These examples suggest that APOBEC mutagenesis may be a good additional tool for evaluating the scale of formation and persistence of R-loops in yeast.

While investigating mutagenesis associated with ssDNA, several potential limitations should be taken in account:

**1) Overcoming high mutation load.** It has long been known that haploid yeast strains with very high spontaneous or induced mutation rates, such as strains carrying a combination of defects in DNA polymerase proofreading and in mismatch repair, or in strains growing in the presence of the very strong mutagen 6-hydroxylaminopurine, hypermutated cells die out due to frequently occurring lethal mutations [235, 247]. However, diploid strains show very high viability because the vast majority of lethal muta-tions are recessive and therefore harmless in a heterozygote. Therefore, the use of diploid strains is advised in conditions when very high levels of APOBEC-induced genome-wide hypermutation is expected.

**2) Non-uniform distribution of ssDNA amount in a population.** If ssDNA formation is occurring in a small fraction of cells with excessive levels of replication, break-repair or transcription problems (concerns even in systems with site-specific DSBs), then choosing candidates for whole-genome sequencing can be problematic. In these cases, the use of specially designed strains allowing selection of cells with clustered mutagenesis in a reporter can be helpful to reduce sequencing efforts [222, 223, 227]. Isolates with a mutated reporter can then be chosen for sequencing of either just the reporter area and/or the entire genome.

**3) Low density of APOBEC-induced cytosine deamination.** APOBECs deaminate less than 1 cytosine per 1000 nucleotides of ssDNA [227], therefore many ssDNA stretches can escape detection. Thus, the results can be interpreted only as a minimum estimate of the amount of long persistent ssDNA in the genome. On the other hand, this is close to the density of UV-, alkylation- (MMS) and chemical deamination- (bisulfite) induced mutagenic lesions in long ssDNA formed at DSBs and uncapped telomeres [221, 222, 225], which aids in predicting ssDNA-associated hypermutation capability in a variety of experimental settings.

**4) Unknown continuity of ssDNA stretches.** A low density of APOBEC mutagenesis would not allow the distinction between long stretches of continuous ssDNA versus relatively short ssDNA regions intercepted with dsDNA. This pitfall may be overcome in the future by developing hyperactive highly processive versions of APOBEC enzymes.

Overall, the patterns and organization of APOBEC-induced clusters and single mutations in model studies may help us to understand APOBEC mutagenesis and ssDNA formation in cancers by analyzing the spectra, mutation signature and topography of mutation calls [223, 248, 249]. Knowledge about the scale and pattern of ssDNA formation is also important for understanding genome wide potential for hypermutation caused by a variety of endogenous and environmental factors other than APOBEC enzymes acting on ssDNA.

## CYTOLOGICAL ASSAYS TO MONITOR DNA REPAIR *IN VIVO*

Cytological assays in living cells are based primarily of spectral variants of genetically encoded fluorescent proteins used to mark DNA repair proteins or chromosomal loci through in-frame fusion to DNA repair proteins or proteins that bind to DNA, respectively. Most microscope systems allow for simultaneous imaging of 3-4 different fluorophores, so that several DNA repair proteins and genetic loci can be imaging in the same cell. Some of these imaging techniques take advantage of photo-activation and photo-bleaching to turn on or off the fluorescence of specific fluorophores, which can be used to obtain information about the dynamics of single molecules in the cell.

The simplicity and amenability of microbial model organisms to genetic manipulation has made it possible to use the cytological approaches described in this section to provide mechanistic insight into the details of DNA repair pathways, including the order of events during repair, the duration of individual repair steps, the proteins and complexes involved in each step, and the correlation of these events with cell cycle stage and location of the lesion within the nuclear.

While often site-specific and inducible DNA lesions are used in population-based assays, the cytological assays can be designed to monitor spontaneous DNA damage caused by endogenous processes in the cell under various physiological conditions. This possibility can allow for a better understanding of natural processes of mutagenesis and genome dynamics.

In this section, cytological assays in *E. coli* and yeast designed to detect DNA damage and monitor repair in living cells are described (**Box 3**).

BOX 3: **CYTOLOGICAL ASSAYS TO MONITOR DNA REPAIR *IN VIVO***

**Detection of double-strand breaks in living cells |** Use of methods to trap and monitor fluorescent proteins that bind to DSB ends. Can be applied to bacterial and mammalian cells.

**Detection of Holliday junctions in living cells |** Use of methods to trap and monitor fluorescent proteins that bind to Holliday junctions..

**DNA repair protein dynamics through single-particle tracking |** Tracks motion of DNA repair proteins to determine diffusion as well as kinetics and spatial distribution of DNA binding.

**FRAP measurements of DNA repair centers |** Methods of fluorescence redistribution after photobleaching to determine dynamic behavior of repair proteins.

**Visualization of chromatin dynamics in cells |** Methods to monitor chromosome dynamics following double-strand breaks. The method can follow chromosomal locus subnuclear mobility and relocalization. Mobility can be quantified using a mean-square displacement calculation.

**Replication fork stalling |** Use of a fluorescently-tagged locus can monitor stalling of replication forks at a specific replication fork barrier. Resolution of a blocked fork in time and space can be determined.

**Double-strand break formation and resection methods |** DSB end resection *in vivo* can be monitored by loss of a fluorescence signal located adjacent to a DNA break. Position and dynamics of the break can be studied simultaneously with break end resection.

### *In vivo* probes to detect DNA repair intermediates

#### Engineered proteins for quantification and trapping of DNA DSBs in living bacterial and mammalian cells

DNA DSBs are transient intermediate structures in genome-instability reactions, including activation of DNA-damage responses and repair. DSBs also occur in programmed developmental processes including meiotic recombination and antibody gene diversification in mammals. Though DSBs can be generated and engineered experimentally for study of repair, an important void in DNA biology has been accurate quantification and detection of the amounts, sources, and frequencies of spontaneous, endogenously generated DSBs in cells: i.e. how important are DSBs relative to other DNA-damage types in genome instability and routine DNA-repair reactions? Several methods used to study DSBs in cells and in genomes have been either non-specific, recognizing various DNA damage substrates or surrogate markers, like single-stranded DNA or DNA-damage-signaling events, rather than specifically DSB ends. There are only two demonstrated highly specific tools for detection of DSBs in bacterial cells, one also useful in mammalian cell culture: (i) genetic studies that compare *recBCD-*null-mutant with wild-type *E. coli* (or other bacterial) cells—phenotype differences indicate a role for DSBs at some stage of the process underlying the phenotype observed; and (ii) engineered fluorescent fusion proteins based on the DSB-end specific binding protein Gam of bacteriophage Mu, which we developed [250]. To our knowledge there is no strictly DSB-end-specific reagent reported yet in yeast models. Because DNA structure is conserved, GamGFP and other Gam-fluorescent proteins (GamFPs) work to label and “trap” DSB ends, blocking repair and other proteins' action on them, when produced in *E. coli*, other bacteria, and mammalian cells.

##### Conceptual description of the method

We engineered gene fusions to encode the phage Mu Gam protein, a double-strand-end specific DNA-binding protein, fused to various fluorescent proteins to create GamGFP, GamMcherry, and others [250]. Mu Gam binds and protects DSB ends from degradation by nucleases and so is a natural DSB-end trap. GamGFP or GamMcherry are produced in *E. coli* from a chromosomally encoded regulatable cassette at a non-genic locus, the phage lambda attachment site. The doxycyline-inducible Δ*attλ*::P_*N25tetO*_*gam-gfp* cassette is transducible into other strains. In mammalian cells the GamFPs are engineered into plasmid vectors that are transfected into cells [250].

##### Uses of GamFPs

i) GamGFP binds and labels DSBs as fluorescent foci in single living bacterial cells

**Figure 14:**
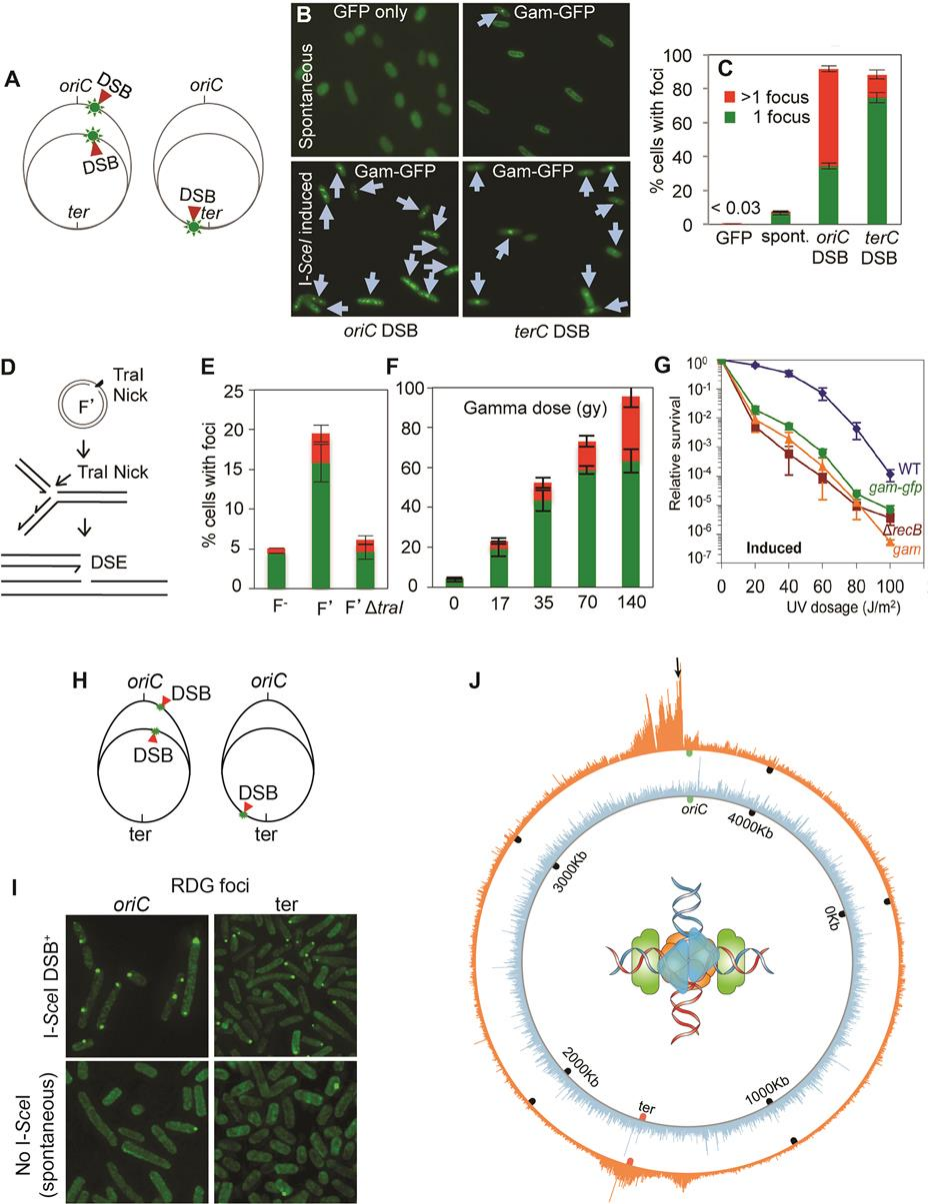
FIGURE 14: GamGFP and RDG quantify DSBs and HJs, map HJ footprints by ChIP-seq in living cells. **(A-G)** GamGFP foci quantify double-strand breaks (DSBs) in living cells and trap DSB ends causing DNA-repair deficiency. Data from [250]. **(A)** Strategy for *E. coli* chromosome cleavage with chromosomally encoded I-*Sce*I ds endonuclease at engineered cut sites (red arrows). **(B)** Representative images of GamGFP foci without I-*Sce*I cleavage (spontaneous, top row) and after I-*Sce*I cleavage at sites either proximal to the replication origin (left column), or distal to the replication origin (right column). In replicating *E. coli* cells, there are more copies of the origin-proximal DNA than the origin-distal sequences, and more DSBs generated, and more GamGFP foci, with origin-proximal than -distal cleavage. **(C)** Numbers of GamGFP foci correspond with expected numbers of I-*Sce*I-generated DSBs. Quantification of GamGFP focus data from multiple experiments diagrammed in parts A and B of this figure. **(D)** GamGFP recognizes one-ended DSBs generated by replication-fork collapse at an enzymatically-generated single-strand nick, made by the TraI single-strand endonuclease at its recognition sequence in a F' single-copy conjugative plasmid. **(E)** Quantification of one-ended DSBs generated by replication-fork collapse at the TraI-generated single-strand nick in the F' single-copy conjugative plasmid. F-, no plasmid. Δ*traI*, no TraI nuclease. **(F)** Linear dose response of GamGFP foci with gamma irradiation. With these data and the known number of DSBs per Gray of gamma in *E. coli*, we estimated the efficiency of GamGFP labeling of DSBs to be about 70% (30% of DSBs are not seen as foci). **(G)** GamGFP production blocks DSB repair, causing a sensitivity to ultraviolet (UV) light similar to that of DNA-repair-defective Δ*recB* null-mutant cells. **(H-J)** RDG foci represent HJs in living cells and RDG ChIP-seq maps HJ footprints. Data from [260]. **(H)** Strategy for *E. coli* chromosome cleavage with chromosomally encoded I-Sce I endonuclease at engineered cut sites (red arrows, DSB) **(I)** Representative images of RDG foci after I-*SceI* cleavage (top row) or spontaneous foci (bottom row), with cleavage near the replication origin (*oriC*) or terminus (ter). **(J)** HJ ChIP-seq at repairing DSBs shows directionality of DSB repair. The orange wheel shows the circular chromosome of *E. coli* bacteria. The spikes indicate where a molecular intermediate in DNA repair— four-way DNA junctions —accumulate near a reparable DSB in the genome (black downward arrow), and also at and after the replication terminus in the chromosome replication path. Blue, RDG ChIP-seq map of an uncleaved *E. coli* chromosome showing sites of spontaneous HJs. Center: diagram of HJ bound by RuvC (blue) assisted by *E. coli* RuvB (green) which stabilizes RuvC on HJs.

**DSB Quantification.** We showed that GamGFP detects DSBs generated site-specifically with I-SceI endonuclease cleavage of a chromosomal cutsite at each of several locations (e.g., **Figure 14A-C**) [250]. We used dose-response curves of ionizing radiation (IR) to estimate the efficiency of labeling of DSB ends based on physical quantification of DSBs at those doses in *E. coli*, and determined that GamGFP labels about 70% of DSBs expected to be present, and its dose-response was linear over a broad range [250] (**Figure 14F**).

**One- and Two-ended DSBs.** One-ended DSBs are generated by a single-strand endonucleolytic cleavage (ssDNA nick) in DNA then replication, which leads to a single DSB end when the fork collapses at the nick (**Figure 14D**, DSE). These were quantified similarly to two-ended DSBs [250]. Our data suggest that the two ends of two-ended DSBs form a single focus.

**Cytogenetic mapping.** Cytogenetic mapping of DSB locations in the *E. coli* chromosome showed that a site bound by a (red) TetR-mCherry repressor protein could be distinguished from GamGFP bound to an I-SceI-endonuclease generated DSB 55kb or more away [250]. At 10 kb apart the foci overlapped. Thus, gross-level mapping of DSB ends with respect to a labeled site in the genome is possible using GamGFP in single living cells.

**Quantification and origins of spontaneous DSBs reveal fewer than predicted.** Using time-lapse microfluidic imaging, we quantified formation of spontaneous GamGFP DSB foci in *E. coli* at various growth rates [250]. We discovered that spontaneous GamGFP DSB foci form in a generation-dependent manner, implicating DNA replication as a component of spontaneous DNA breakage. Because GamGFP traps DSB ends preventing repair (e.g., **Figure 14G**), once formed, the foci remained visible for at least 18 h, and the cells with foci stopped dividing. The rate of spontaneous DSB formation detected with GamGFP was 0.015 ± 0.006 DSB foci per cell division [250], a rate in agreement within a factor of two with our previous quantification of spontaneous DSBs measured somewhat more indirectly as the RecBCD-dependent component of spontaneous SOS-response-positive cells, measured using flow cytometry and a chromosomal SOS-activated promoter fused to *gfp* also at a non-genic site: *attλ*::P_*sulA*_*gfp* [251]. An mCherry version of this SOS-responsive DNA-damage detection cassette now also exists and is highly sensitive [252]. Importantly, with both GamGFP or SOS flow-cytometric assays, DSBs arise about 50-times less frequently than the once per *E. coli* cell division predicted from indirect estimates of DNA breakage by many previous authors. The standardization of both methods with known physically detected DSBs after IR or by I-SceI induction in *E. coli* [250, 251], and the agreement between the different methods support these rates as accurate.

ii) Blocking DSB repair in *E. coli*

GamGFP blocks repair of DSBs causing phenotypes in *E. coli* like those of RecBCD-null mutant cells. These include sensitivity to UV light (**Figure 14G**) and large plaque formation by phage lambda recombination-defective and RecBCD-inhibitor-defective *red gam* mutants [250]. GamGFP is thus useful for blocking the action of other proteins on DSB ends, including stopping repair mechanisms.

iii) General Utility

Several other studies of bacterial cells have used GamGFP successfully to block DNA repair [253] or detect DSBs as fluorescent foci [254–259] including our use of Gam to help quantify the efficiency of recognition of four-way DNA (Holliday) junctions in repair by RuvCDefGFP (RDG) [260], discussed in this paper.

iv) Mammalian Cells

Several groups have now used GamGFP and derivatives for detection of DSBs or inhibition of repair of DSB ends in mammalian cells [250, 261–263]. We first showed that GamFP fusion proteins label laser- and IR-induced DSBs as fluorescent foci, and block the early stages of DSB repair by inhibiting exonucleolytic resection that creates single-stranded DNA, detected by RAD51 binding and immunofluorescence [250]. Phage Mu Gam is an orthologue of mammalian (and bacterial) Ku, and we found that the labeling of DSBs was more efficient in Ku-defective than wild-type mouse cells, implying that Ku may compete with GamFPs for DSB end binding [250]. We also used GamGFP in human and mouse cells to demonstrate that foci of gamma-H2AX histone staining really do label DSBs specifically [250]. However, another commonly used DSB/DNA-damage marker, 53BP1, did not label all GamGFP-labeled foci and appeared to label some sites other than GamGFP-labeled DSBs, calling into question the DSB-specificity of its labeling [250].

##### Cautionary notes

Quantification of DSBs in *E. coli* and other bacteria by GamFP foci:

**Focus quantification challenge**: when analyzing images taken from deconvolution microscopy, false positives may occur. Projected images of raw Z-stacked images may artificially generate images of cells with denser polar pixels, which may be mistakenly identified as foci. These artifacts can by filtered by applying a shape filter in the focus analysis software.

**GamGFP foci are brighter than GamMcherry foci**, in our hands. This suggests that the FP multimerization possible with GFP but not mCherry aids formation of detectable foci.

**Cell sizes and numbers of chromosomes can vary** between different genotypes. During an SOS DNA-damage response when cells “filament” because of inhibition of cell division, forming long cells with multiple chromosomes, cell length can be >5 times longer than normal [264]. Thus, normalizing numbers of foci/cell to DNA content is critical to accurate determination of the number of DSBs per chromosome or DNA amount in cells [250].

**GamGFP is a “freeze-frame protein” trap**. Because GamFPs bind DSB ends and inhibit their repair, cells with DNA damage are counterselected from growing populations because they cease to proliferate upon binding of GamFPs [250]. Care should be taken with extended growth with induction of GamFPs.

**Protein levels of GamFPs must be comparable** between different genotypes compared for focus levels to reflect DSB-formation rates. Because focus accumulation is cumulative—foci formed do not disappear, we showed [250]—similar lengths of induction time with similar inducer concentrations should be used.

**Background**. Both for *E. coli* and mammalian cells, the cell or nucleus becomes green when GamGFP is produced. Higher contrast between foci and other space than seen in live cells can be obtained in fixed cells, in which free GamGFP is removed. However live-cell imaging in *E. coli* is quantitative and straight forward [250].

#### Engineered proteins for quantification, trapping and genomic mapping of Holliday junctions in living bacterial cells

Holliday junctions (HJs) are transient DNA reaction-intermediate structures with four double-stranded arms. HJs are central intermediates in DNA recombinational repair, and also occur when replication is stalled and a fork "reverses." HJs are intermediates in, and can lead to, genome instability. Despite the centrality of HJs to DNA repair, replication, and genome-instability mechanisms, the ability to visualize, quantify, and map HJs in genomes of living cells has been limited, as has our understanding of the proteins that create, prevent, and remove them.

##### Conceptual description of the method

We engineered proteins that trap, map, and allow quantification of HJs in living *E. coli* cells and biochemically [260]. The new HJ visualization, quantification and mapping approaches are based on RuvCDefGFP (RDG), a GFP-tagged catalytically inactive version of the *E. coli* RuvC endonuclease. RuvC is the most four-way DNA-junction-specific protein known [265–267], and RDG can bind to but not cleave HJs [260].

We showed that RDG binds HJs and inhibits the action of other HJ-processing proteins on them both biochemically and in living cells [260]. That is, RDG “traps” HJs. RDG also forms quantifiable fluorescent foci that are correlated with HJs (representative images of foci in *E. coli* in **Figure 14H-I**), and is estimated to detect about half of HJs present in *E. coli* [260].

Moreover, the locations of HJs can be mapped in the *E. coli* genome using RDG chromatin immunoprecipitation followed by sequencing (ChIP-seq) [260]. We used RDG ChIP-seq to generate high-resolution maps of HJ footprints during repair of site-specific DSBs (**Figure 14J**) [260]. Using this powerful tool, we demonstrated the genome-scale directionality of homology-directed repair (HDR) of DSBs: that HJs accumulate at a site-specific DNA break undergoing repair and downstream from it in the chromosome replication path. These data provided experimental evidence for models of one-ended HR-DSB repair in the *E. coli* genome [268], in which the DSB-end attached to the replication origin primes processive break-induced replication that drags a HJ to the replication terminus [260, 269].

##### Uses of HJ-trap proteins

**RDG binds and labels HJs in single living cells.** There are many key outstanding questions in genome instability and replication that can be addressed using HJ trap proteins. Various assays measure HDR of, e.g., artificially induced DSBs, and have illuminated several HDR mechanisms. Yet, little is known about which mechanisms predominate spontaneously in somatic or vegetative cells. The HJ trap protein can detect and label HJs as fluorescent foci, thus enabling exploration of the following questions: What are the primary uses for and instigators of HDR in vegetative/somatic cells? How important is (what is the frequency of) HDR repair? RDG focus quantification combined with genetic analysis allowed us to address these questions [260]. We found that *spontaneous* HDR-HJs are replication-dependent, mostly resulting from non-DSB damage (ssDNA gaps) in vegetative *E. coli.* We also discovered the rates of formation of HDR-HJs, and that that their main sources are single-stranded DNA gaps, not DSBs. Recently, high-throughput microscopy techniques have been developed to allow genetic and chemical screening using RDG foci. In addition, the dynamics of HJ formation in living cells can be measured by tracking RDG foci in living cells by time-lapse imaging [260].

**RDG for dissecting the stage(s) of HDR at which elusive HDR proteins act.** Some proteins are required for HR repair but their primary role(s) are ambiguous. Trapping and quantifying HJs during HR repair allowed us to assess whether HDR proteins act before or after HJ formation. Timed expression of RDG and focus quantification can be used to dissect the stages at which recombination proteins act. Using this approach, we discovered a new “junction-guardian” role of *E. coli* RecQ DNA helicase: promoting HDR-HJs and preventing reversed-fork HJs. The-fork-reversal-prevention role was also supported by bioinformatic data in human cancers for two human RecQ orthologs: BLM and RECQL4 [260].

**ChIP-Seq approach combined with genetics.** ChIP-Seq is a technique for genome-wide mapping of DNA-interacting proteins. We generated a high-resolution genome-wide map of the HR repair landscape upon I-*Sce* I induction of DSBs (**Figure 14J**). These first glimpses of the genomic footprints of HJs during DSB repair demonstrate a directionality of DSB repair along the chromosome not observed previously, with more HJs ori-distally than ori-proximally of the DSB site. We have applied the ChIP-Seq technique with classical genetic analyses to understand DSB repair [260].

ChIP-seq peaks are prone to artifacts, so proper controls and careful experimental designs are essential for reliable interpretation of the results. Among these controls, the genetic controls, which we used in generating site-specific DSB-induced HJ maps, give the most definitive answers.

##### RDG: a candidate universal HJ trap

RDG may potentially act as a universal HJ trap for organisms other than bacteria due to the conserved DNA structure of HJs. This has been supported by a recent study showing that RuvCDef binds t-loop generated HJs in human telomeres [270].

##### Cautionary notes

i) Quantification of HJs in *E. coli* as RDG foci:

**Focus quantification challenge**. When analyzing images taken from deconvolution microscopy, false positives can occur. Projected images of the raw Z-stacked images may artificially generate images of cells with denser polar pixels which may be mistakenly identified as foci. However usually these artifacts can by filtered by applying a shape filter in the focus analysis software.

**Cell sizes and numbers of chromosomes can vary between different genotypes.** For example, in some “filamented” cells—*E. coli* that have undergone an SOS DNA-damage response, with cell division block and thus have multiple chromosomes per cell—cell length can be >5-times longer than normal [264]. Thus, normalizing numbers of foci/cell to DNA content is critical to accurate determination of the number of HJs per chromosome or DNA amount in cells [260].

**Expression levels of “trap” proteins must be comparable when comparing different HJ levels between genotypes.** The easiest way to ensure this is measuring mean/median florescence intensity from the GFP in flow cytometry. In addition, prolonged trap-protein production is not recommended because trapping HJs can be toxic to cells, preventing proliferation [260].

**Limited throughput**. The numbers of cells that can be generated per image are still limited. Although a high-throughput-screening approach has been developed, only 10^5^ cells can be imagined per hour. Additionally, the large numbers of raw and deconvoluted images require significant data storage.

ii) ChIP-Seq:

**Sources of antibody** are critical to ChIP-Seq [271]. Although the commercial RuvC antibody we used (Santa Cruz Biotech) has been validated extensively, using antibodies from other sources to ChIP RDG requires further evaluation.

**Low data quality** such as low sequencing coverage will limit the sensitivity of ChIP signal [272]; so it is advisable that samples should have sufficient and similar sequencing depth.

**ChIP-Seq displays profiles of bulk cell populations** and does not provide single-cell resolution as foci do. Small peaks may be ambiguous due to high background signal; thus, use of multiple peak-calling algorithms is recommended for noise reduction. More importantly, genetic controls are the gold standard for interpreting peaks. To further enhance signal/noise ratio, it may be helpful to enrich target populations by cell sorting or other physical methods and to increase total volumes of cells.

### Methods to determine DNA repair protein dynamics in the cell

#### Single-particle tracking of nucleotide excision repair proteins inside living bacteria

Single-particle tracking (SPT) combined with Photoactivated Localization Microscopy (sptPALM) provides an opportunity to perform complex molecular biology experiments inside living cells. By tracking the motion of DNA repair proteins *in vivo*, information can be extracted not only about their diffusion, but also about the kinetics and spatial distribution of DNA binding [273–275]. From a methodological point of view, a Total Internal Reflection Microscope equipped with a sensitive detector (usually an EM-CCD camera [276]) is commonly used, allowing detection of individual fluorophores. The signal from individual emitters can be analysed and the position of a given fluorophore established with high accuracy (up to a single nm) by Gaussian fitting. To determine the mobility of each fluorophore, the positions of individual molecules are linked into trajectories over multiple frames using a tracking algorithm [277].

Since most proteins in bacteria are present at a copy number, which is too high to resolve individual fluorophores, photoactivable fluorescent proteins (PAFPs i.e. PAmCherry) can be used, allowing the level of active fluorophores to be controlled (e.g. by varying the intensity of a 405 nm photoactivation laser) such that ∼1 fluorophore is active per cell. This allows for the consecutive observation of all labelled proteins [273–275]. As an alternative to PAFPs, protein tags (i.e. HaloTag), which bind organic fluorophores provided externally can also be used. Once a functional fusion of the protein of interest to a fluorescent label has been constructed, the experiment can begin.

**Figure 15:**
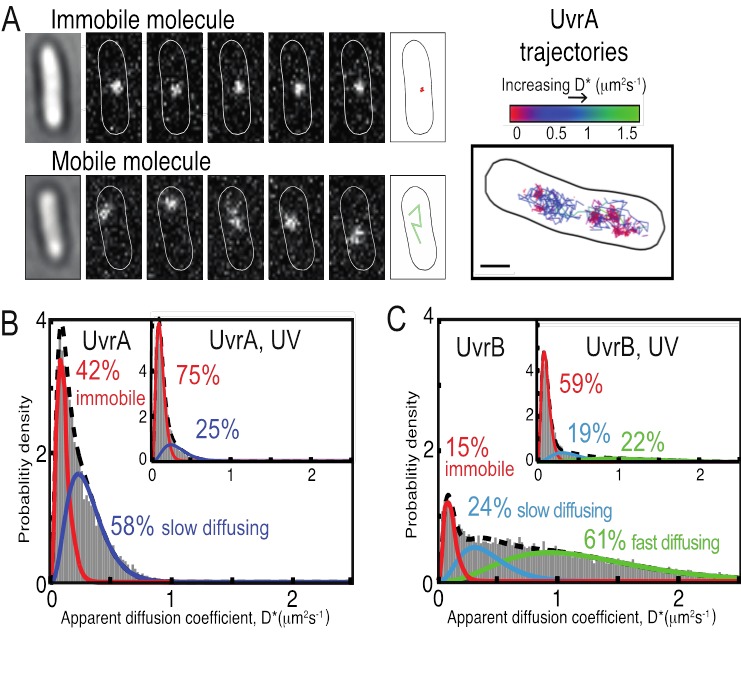
FIGURE 15: *In vivo* characterization of UvrA and UvrB proteins. **(A)** The example image of a single immobile UvrA-PAmCherry molecule localized and tracked at 15 ms exposures within five consecutive frames (top) and the example image of five consecutive frames showing fast diffusing UvrB-PAmCherry molecule (bottom). On the right, example cell is shown with multiple trajectories recorder for many individual UvrA molecules. **(B)** Distribution of apparent diffusion coefficients (D*) of tracked UvrA molecules, fitted with a two species model: first immobile, DNA-bound population (∼42%) and second mobile population of slowly diffusing molecules (∼58%). (Inset) The distribution of D* values of tracked UvrA molecules after exposure to 50 J m^−2^ ultraviolet light (UV). **(C)** Distribution of D* values of tracked UvrB molecules, fitted with a three species model established that ∼15% of UvrB molecules were immobile, ∼24% diffusing slowly and ∼61% fast diffusing. (Inset) The distribution of D* values of tracked UvrB molecules after exposure to ultraviolet light (UV).

One example of the power of sptPALM was a study of the NER pathway in *E. coli[278]*. Fusions of UvrA and UvrB - the proteins that initiate NER, to PAmCherry were introduced into the chromosome and their behaviour was studied in cells, before and after DNA lesions were induced by exposure to UV light. A movie was recorded with 15 ms frame rate and the positions of the fluorophores were localised in each frame and linked into trajectories. For each trajectory, an apparent diffusion coefficient was calculated based on the distance between subsequent localisations [277] (**Figure 15A**). Molecules bound to DNA showed a minimal change in position, whereas freely diffusing molecules showed large displacements between consecutive frames. The different populations of UvrA and UvrB molecules were quantified by fitting the distribution of apparent diffusion coefficients from tens of thousands of molecules (**Figure 15B, C**). UvrA was found to bind DNA stably for ∼3 s (∼40% of molecules) or interact with DNA transiently (low ms range) (**Figure 15B**). It was proposed that the transient protein-DNA binding is a part of the initial DNA search process, whereas longer binding is a damage verification step. On the other hand, UvrB showed very different behaviour, with the majority of UvrB molecules freely diffusing throughout the cytoplasm (**Figure 15C**). Cell exposure to UV light caused the recruitment of most UvrA and UvrB molecules to DNA (75% and 60% of molecules, respectively) to repair UV-induced lesions. These sptPALM experiments showed that, in contrast to some historical *in vitro* experiments, UvrA and UvrB rarely form a complex in solution; instead, UvrA is a DNA damage sensor, recruiting UvrB to DNA only after damage detection. Furthermore, by using catalytic mutants of UvrA, it was possible to decipher the roles of the two ATP binding sites present in each UvrA molecule, showing that cooperative action in both sites is necessary to recruit UvrB to DNA damage sites [278].

##### Cautionary notes

Previously, one of the key factors preventing the wider adoption of SPT and sptPALM in microbiology has been limited access to the sophisticated equipment and custom-written data analysis tools required for imaging single molecules. However, as these techniques increase in popularity, commercial single-molecule microscopes are becoming more affordable and data analysis tools for SPT are becoming more available [279], opening access to this technique for non-specialist users.

Last but not least, the critical step in all SPT experiments is the construction of a fusion between the protein of interest and the fluorescent tag. Occasionally, this results in an inactive protein. Therefore, the functionality of each fusion protein must be carefully verified. If the fusion is non-functional, the fluorescent tag can be placed at the other end of the protein or the length and nature of the linker can be adjusted to suppress the interference of the tag.

##### Conclusion

SPT is a powerful method, allowing biochemical experiments to be performed in the native environment of living cells. When combined with perturbations such as protein mutations, deletions, or overexpression it can be used to gain deep mechanistic understanding of molecular pathways, and it has been applied not just to the field of DNA repair [274, 275, 280], but also to study the stringent response [281], transcription [273, 282] and translation [283]. SPT is becoming more and more popular, not only in the field of microbiology but also in the eukaryotic field [284]. Furthermore, the availability of user-friendly microscopes and analysis tools [285] will pave the way for STP to become a standard tool in any laboratory.

#### Inter-foci fluorescence redistribution after photobleaching (iFRAP) to measure dynamics of DNA repair centers

DNA recombination and repair involve the assembly of large protein complexes at the site of DNA damage. The high local concentration of DNA repair enzymes at sites of DNA damage can be examined in living cells by GFP-tagging of the involved proteins, which results in the appearance of cytologically discernable foci [286]. These repair foci are highly dynamic in time and space with proteins associating and dissociating from the site of DNA damage on a time scale of seconds to minutes. The dynamic behavior of DNA recombination foci *in vivo* contrasts the very stable recombination complexes formed *in vitro* [287], suggesting that chaperones, remodelers and segregases may play a role in modulating the organization of the DNA recombination and repair machinery *in vivo*. The dynamic redistribution of proteins in living cells can be monitored by various photobleaching techniques such as fluorescence loss in photobleaching (FLIP) and fluorescence redistribution after photobleaching (FRAP), where fluorescently tagged proteins are locally photobleached by a brief exposure to intense laser light and the kinetics of redistribution of fluorescence due to diffusion of fluorophores from neighboring areas recorded. In the case of foci, this allows for the calculation of association/dissociation rates and mobile/immobile fractions to be quantified. In organisms with small size e.g. yeast and bacteria, these photobleaching techniques are challenged by the high rate of diffusion of most proteins, which results in complete redistribution within a time frame that is typically shorter than the time required for efficient photobleaching. For example, the mean squared displacement of GFP in the mammalian nucleus has been measured to 50 µm^2^/s [288], which would allow it to diffuse across the 2 µm of the yeast nucleus in approximately 0.01 s. As a consequence, the photobleaching of a focus, which typically takes 0.5 s, would lead to the simultaneous bleaching of the entire pool of soluble fluorescent protein in the nucleus. To address this challenge, we have established the inter-foci fluorescence redistribution after photobleaching (iFRAP) technique, where redistribution is monitored between two DNA repair foci within the same cell. In this technique, one focus is bleached, while the other focus serves as a pool for unbleached fluorescent molecules during the subsequent redistribution.

##### Description of the iFRAP method

In *S. cerevisiae*, the iFRAP method is best applied to recombinational repair foci after induction of approximately two-three DNA DSBsper cell. It is an advantage to use diploid cells, because their nuclei are slightly larger (Ø = 2.3 µm) than those of haploid cells (Ø = 1.8 µm) [289], making it easier to identify cells with two foci that are separated by more than 1 µm, which is the minimum distance required to photobleach one focus without collateral bleaching of the adjacent focus with most microscope configurations. Under standard time-lapse imaging conditions [290], two-three DSBs per diploid cell can be induced by treatment with 200 µg/ml Zeocin for 2 h or 30 Gy of ionizing radiation. After this treatment, 10-20% of the cells will have two foci of for example the Rad52 recombination mediator or the single-stranded DNA binding protein RPA (Replication protein A) [291], while most of the remaining cells have one focus. The low number of cells with two recombination foci is due to the clustering of multiple DSBs at a single recombination focus [292]. The iFRAP calculations are based on a time series of Z-stack images. We usually acquire one Z-stack before photobleaching and nine stacks after photobleaching. Some fluorophores bleach more readily than others depending on the laser wavelength. We have good experience with bleaching of enhanced GFP [293] and yellow fluorescent protein (YFP, clone 10C) [294] using a 50 mW 488 nm laser.

**Figure 16:**
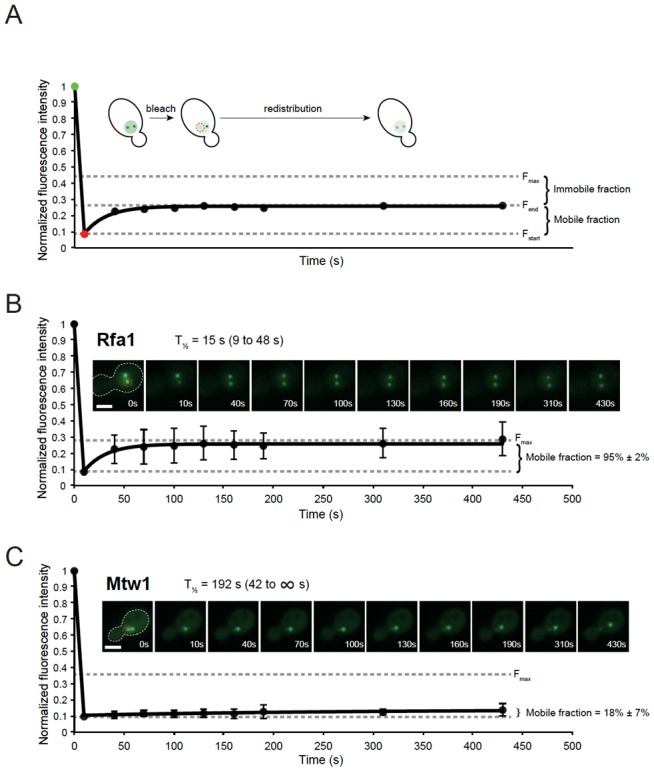
FIGURE 16: iFRAP analysis of Replication protein A and kinetochore subunit Mtw1. **(A)** Principle of iFRAP method. An image is acquired immediately before (green data point) and after (red data point) laser bleaching of one of two subnuclear foci (indicated by dashed red circle). The fluorescence intensity of the focus before bleaching is set to 1 and all subsequent fluorescence measurements are normalized accordingly. F_start_ indicates the fluorescence intensity of the bleached focus immediately after laser bleaching. F_end_ indicates the plateau fluorescence intensity that the bleached focus approaches during redistribution. F_max_ indicated the maximum fluorescence intensity that the bleached focus can reach at the end of the experiment assuming 100% redistribution. The mobile protein fraction can be calculated as (F_end_ − F_start_)/(F_max_ − F_start_). **(B and C)** Cells with two foci were subjected to photobleaching of one focus (indicated by dashed red circle) at t = 8s. Fluorescence redistribution was quantified at subsequent time points. The redistribution half-time (T_1/2_) is indicated with 95% confidence intervals in parentheses. Scale bar, 3 µm. The protein mobile fraction is indicated ± standard deviation. **(B)** RPA dynamically exchanges at DNA damage-induced foci. Cells expressing Rfa1-YFP (ML306) were grown in the presence of 200 µg/ml Zeocin for 2 hours to induce DNA repair foci. **(C)** Mtw1 is a stable component of the kinetochore. Cells expressing Mtw1-GFP [368] were arrested in M phase by treatment with 10 µg/ml nocodazole for 2 hours. One-phase association non-linear regression was performed using GraphPad Prism software. Error bars indicate SEM.

After image acquisition, three fluorescence measurements are performed for each time point: the total nuclear fluorescence, the fluorescence of the bleached focus, and the fluorescence of the unbleached focus. Next, all fluorescence values are background-subtracted. Since image acquisition also causes a low level of photobleaching, the total nuclear fluorescence will continue to decrease in an exponential manner with each image acquisition. This bleach factor is determined by curve-fitting the total nuclear fluorescence for each time point after the laser event to an exponential decay function. The bleach factor allows for all fluorescence values after laser bleaching to be adjusted to compensate for the photobleaching caused by image acquisition. The maximum normalized fluorescence intensity F_max_ that the bleached focus can reach at the end of the experiment assuming 100% redistribution equals the total nuclear fluorescence after laser bleaching divided by the total nuclear fluorescence before laser bleaching. The recovery half-time T_½_ and plateau fluorescence F_end_ that the bleached focus approaches during redistribution is determined by curve-fitting using one-phase association non-linear regression. By comparison to the fluorescence of the bleached focus immediately after laser bleaching, F_start_, the mobile protein fraction can be calculated as (F_end_ − F_start_)/(F_max_ − F_start_) (**Figure 16A**).

In the presented examples (**Figure 16B and C**), the ssDNA binding protein Rfa1 rapidly turns over at the site of DNA damage and the Rfa1 molecules in foci are almost 100% mobile (**Figure 16B**). The turnover of Rfa1 is slower than the Rad52 recombination protein, but faster than the Rad51 recombinase [287], which could reflect the need to dissociate RPA from single-stranded DNA in order to nucleate a Rad51 filament. In comparison, the kinetochore subunit Mtw1 only slowly redistributes between the bleached cluster of kinetochores and the unbleached kinetochores (**Figure 16C**), and the majority of Mtw1 is immobile. This behaviour of Mtw1 is consistent with Mtw1 being a stable subunit of the MIND kinetochore subcomplex, which joins kinetochore subunits contacting DNA to those contacting microtubules [295].

##### Cautionary notes

The degree of laser photobleaching should be selected to allow for the most accurate quantitation. We have good experience with bleaching the target focus to around 10% of its original intensity, but for low abundant proteins such as Rad54, limiting the bleaching to 20% of its original intensity improved the accuracy of the subsequent quantitation [287].

The fluorophore should be selected to avoid reversible photobleaching, which has been observed for some variants of GFP, especially at low pH [296]. This is easily controlled for by measuring the total nuclear fluorescence during the redistribution phase. No reversibility was observed for the enhanced GFP [293] and YFP [294] used in this study.

Post-acquisition fluorescence measurements are greatly facilitated if cells with foci of roughly equal size are selected for iFRAP. This is due to the greater accuracy associated with measuring the fluorescence intensity of relatively bright foci.

The advantage but also a potential limitation of the iFRAP method is that the rate of diffusion from one focus to another is a composite of the on-off rate at the unbleached focus and the on-off rate at the bleached focus, whereas the diffusion in solution for most proteins will be several orders of magnitude faster and therefore negligible. The iFRAP method does not allow us to independently determine the on-off rate at the unbleached focus and the on-off rate at the bleached focus. Thus, the on-off rates for the bleached protein may be different for the two foci of a cell. In the context of DNA repair, such differences could reflect different on-off rates at different stages of repair. Moreover, most genotoxic agents, including Zeocin, induce DNA damage at random in the genome and the biophysical properties of repair foci could be different at different genomic loci.

##### Conclusion

The iFRAP method offers a tool to study the dynamics of DNA repair foci *in vivo* in microorganisms and to uncover the role of molecular chaperones, segregases and remodelers in facilitating the assembly and disassembly of DNA repair machinery at sites of DNA damage. This will potentially help explain some of the differences observed between the *in vitro* biochemical properties and *in vivo* cytological behavior observed for DNA repair proteins [287].

### Methods to determine chromatin dynamics in the cell

#### Visualizing chromosomal dynamics following DNA DSB induction in yeast S. cerevisiae

DNA DSBs are the most deleterious type of DNA damage when they are not repaired by an error-free mechanism. Thus, understanding their behavior in living cells is of major importance. Using *S. cerevisiae* as model organism, where most of the DNA repair proteins are similar to those in humans, allows us to use genetic tools to characterize the properties of DNA DSBs. Using live microscopy, one can visualize the subnuclear localization of DSBs and examine how changes in position can influence the pathway of repair and/or repair efficiency. Three-dimensional tracking of a single DSB enables an in-depth characterization of the motion of the chromatin locus, and allows one to model it as a polymer fiber.

##### Determining chromosomal locus subnuclear position following DNA damage

**Figure 17:**
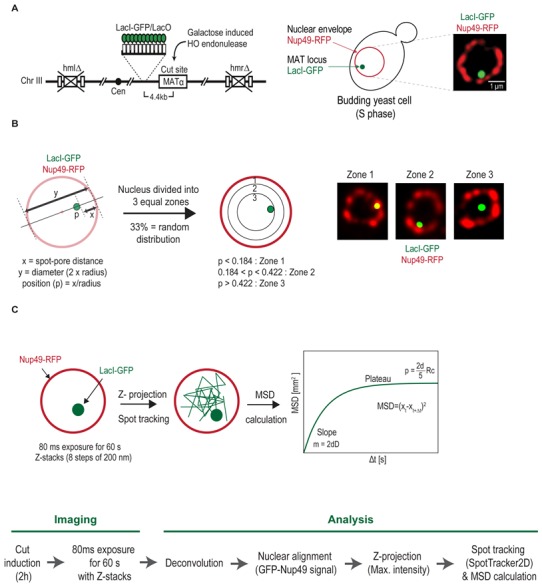
FIGURE 17: Visualizing chromosomal dynamics at a DNA DSB in the yeast *S. cerevisiae*. **(A)** Schematic representation of the chromosomal locus tagging system. HO endonuclease is used to generate a single DSB at the mating type locus (*MAT* locus) that is tagged with a lacO array. The LacO array binds the LacI repressor fused to a green fluorescent protein (LacI-GFP). The nuclear envelope is visualized using a fluorescently tagged nuclear pore protein (Nup49-RFP). **(B)** Analyzing chromosomal locus position. Relative locus position (p) is calculated by normalizing the distance pore-locus (x) by the nuclear radius (y/2). The radial distances are then classified into three groups - Zone 1 (peripheral width=0.184 x nuclear radius(*r*)), 2 (middle width between 0.185*r* and 0.422*r*) and 3 (central width=0.578*r*) - of equal surface. **(C)** Analyzing chromosomal locus mobility. Overview of the imaging procedure. Single cell, multi-stack images are acquired every 80 ms for 60 s. After deconvolution and nuclear alignment, the 3D images are converted to 2D using a maximum z-projection. The tagged locus is tracked using SpotTracker2D and an absolute MSD calculation is applied.

It has been shown in budding yeast that when a DSB cannot be immediately repaired by recombination with its sister chromatid, it relocates to the nuclear periphery where it binds either the Nup84 nuclear pore subcomplex or an inner nuclear membrane SUN-domain protein, Mps3. The relocation and interaction with these two distinct sites has different effects on repair outcome, given that pore mutants and Mps3 mutants lead to different repair outcomes very differently. A three-zone technique for determining the position of damaged or undamaged loci [297, 298] has been useful for determining the precise positioning of the damaged locus relative to the nuclear envelope. One version of this imaging technique takes advantage of the bacterial Lac operator (*lacO*) sequence which binds the LacI repressor fused to a green fluorescent protein (LacI-GFP). One can then exploit a site-specific budding yeast endonuclease to create a single HO endonuclease cut at specific loci that are tagged by a *lacO* array. This permits a highly accurate determination of the subnuclear position of the induced DSB. The nuclear periphery is generally identified with a fluorescent tag (e.g., RFP) on the pore protein Nup49 (**Figure 17A**). Often the mating type locus, MAT, is tagged with lacO repeats and tracked, as it is the physiological target site of the HO endonuclease.

**Method and data analysis.** By placing the HO endonuclease under control of the *GAL1* promoter, cleavage can be induced by the addition of 2% galactose for up to 2h. Cut efficiency is quantified by qPCR. Multi-stack images are acquired immediately, usually with a spinning disk confocal microscope. Spherical nuclei are needed for a proper statistical analysis of relative subnuclear positioning, thus G1 or early S phase cells are usually analyzed, using the imageJ plugin *Point Picker* [298]. Briefly, the positions of the DSB and diameter of the nuclear pore-defined circle are determined in the same plane of focus. Using a pre-designed excel sheet, one can calculate the diameter of the nucleus, set this to one, and then determine a relative value for the distance between the periphery and the spot (**Figure 17B**). Hundreds of such measurements are taken for a given time point. To confirm that the site monitored is actually cleaved, it is often helpful to express a Rad52-YFP fusion, which will colocalize with the DSB.

Using a nuclear pore mutant *nup133*Δ*N*, which forces the majority of nuclear pores to form a large, single cluster at the nuclear periphery, one can accurately score the co- localization of the DSB with nuclear pores. On the other hand, DSB binding to Mps3 is best scored by Mps3 Chromatin Immunoprecipitation, because Mps3 gives only a weak fluorescence at the nuclear rim, apart from the spindle pole body. There may be additional perinuclear anchorage sites that remain to be characterized.

**Cautionary notes.** This technique relies on accurate dual-color imaging techniques, and it is crucial to take into account and correct for emission wavelength phase shifts. To score accurate distances, it is important only to score nuclei if the fluorescent spot is within the middle 50% of the Z-stack (not in the upper 25% or lower 25% of the nuclear sphere), due to the fact that resolution in the Z direction is less good than in the X-Y plane. One should not use DNA fluorescence as the boundary of the nucleus because the edge depends on thresholding, which is often subjective. Therefore, we recommend introducing a nuclear pore marker is essential for proper three-zone scoring. For a theoretical discussion of why the three-zone measurement is appropriate for such analyses, and the error inherent in the method, see our discussion of Cavalieri's principle and its validation through both empirical and theoretical troubleshooting [297].

##### Analyzing chromosomal locus mobility upon DNA damage

It has been shown that chromatin dynamics increase upon DNA DSB induction [321, 322]. This probably improves repair efficiency by accelerating the homology search or by favoring relocation to a repair center. Using a similar cellular system for cut induction, as described above for the three-zone method of DSB localization (**Figure 17A**), a live cell tracking strategy has been developed to monitor DSB mobility over time.

**Method and analysis.** High speed time-lapse fluorescence microscopy allows one to track the LacI-GFP tagged locus over time in living cells. In this case the center of the nucleus is used as a reference to correct for nuclear oscillations or translational movement of the entire field of vision. Different scales of image capture (i.e variation in the interval between image capture as well as the time of image capture) can be used. One system that has been useful has been the acquisition of single cell images every 80 ms taking Z-stacks of 200 nm for 1 min overall imaging time. Alternatively, longer times between stack can be introduced and image capture can be extended to 5 or 7 min. One must always monitor for laser- or light-induced cellular damage, given that activation of a checkpoint response may bias subsequent measurements.

After deconvolution and Z projection, the ImageJ plugin *Spot Tracker 2D* can be used to track the LacI-GFP-tagged fluorescent locus relative to the Nup49-RFP-tagged nuclear envelope (**Figure 17C**). To compare locus position from one image stack to the next, one must align the nuclear centers through means of an idealized circle (the nuclear perimeter), and then determine the movement of the tracked locus within the aligned nuclear sphere. To quantify locus mobility, the mean square displacement of the fluorescent locus is calculated over time using a pre-designed excel sheet [299]. Mean-squared displacement is described in detail below.

Biophysical parameters derived from polymer model analysis can be applied to the spot trajectories to further characterize the motion. Briefly, the length of constraint Lc measures the locus confinement in distance traveled, the effective diffusion coefficient Dc reflects its velocity, the effective spring coefficient Kc estimate the forces acting on this specific locus and the anomalous exponent α describe the nature of the motion [300].

**Cautionary notes.** The quantification of the trajectories relies directly on the imaging scale used. Recently, a study showed that changes in chromatin dynamics upon DNA damage depend on the tracking scale used [301]. It is therefore crucial to take into account this variation when choosing the time interval used for imaging. Basal levels of chromatin mobility (i.e without damage) vary during the cell cycle, and chromatin in G1-phase cells, is more mobile than in S/G2. This has been attributed to the loading of cohesin in S/G2, to hold the two sister chromatids together after replication. Its removal and/or degradation allows an increase in the chromatin mobility [302]. Thus, to eliminate cell cycle -induced variability in movement, it is essential to compare mobility changes within the same cell cycle stage. For determining cell cycle stage accurately, see Neumann *et al.* 2006 [303].

Using *S. cerevisiae* as model organism allows us to accurately characterize chromatin dynamics following DNA damage. The use LacO/LacI arrays for chromatin tagging enables scientists to determine specifically the position and dynamics of chromatin loci in response to DSBs. We and others have shown that chromatin dynamics and re-localization plays a central role in response to DNA damage, but how this impacts DNA repair efficiencies remains unclear.

### Quantifying the mobility of a chromosomal locus

The dynamic organization of the genome is essential for many biological processes such as transcription, DNA repair, differentiation, etc. Indeed, the mode of diffusion of a chromosomal locus inside the nucleus dramatically affects the speed with which it interacts with surrounding molecules as well as with other chromosomal loci [304]. The mode of diffusion of a locus reveals aspects of how it explores nuclear space, how it deals with the obstacles it encounters, and how its movement relates to the organized structure of the nucleus. Thanks to the development of advanced microscopy techniques during the last ten years, it has become possible to measure and quantify chromatin mobility in living cells with unprecedented resolution.

#### How to measure chromatin mobility

The most common method to measure chromatin mobility consists of inserting a fluorescently tagged array at a given genomic locus and measuring its position through time. To tag a locus, repeated bacterial sequences, such as Lac-Operator (*lacO*) or Tet-Operator (*tetO*) arrays are inserted in the genome [305]. These arrays are bound by Tet-Repressor (TetR) and Lac-repressor protein (LacI), which are fused to fluorescent proteins. The genomic locus is visible as a fluorescent spot by wide field microscopy and can be tracked over time. It should be noted that, in some cases, tightly bound LacI and TetR repressors can create fragile sites or constitute a barrier of unknown penetrability to DNA processing enzymes [306–308]. To overcome these barriers, variants of LacI, such as the LacI** mutants [309], have been used as alternatives to bypass any possible bias. Another tagging method, consisting of the ParB-INT DNA labelling system, has also been developed to fluorescently mark genomic loci [310].

To measure accurately chromatin motion, it is essential to correct for the motion of the nucleus during the acquisition of images. One method uses a fluorescent marker in the nuclear membrane to subtract the nuclear motion from the motion of the tagged genomic locus [311]. Alternately, it is possible to fluorescently mark a point that is relatively immobile in the nucleus during the time of the acquisition, such as the spindle pole body [312]. Finally, it is also possible to fluorescently mark two genomic loci and measure their relative positions over time. However, this method is used only when both loci have the same diffusion properties since it does not measure their motion independently [312, 313].

#### How to quantify chromatin mobility

**Figure 18:**
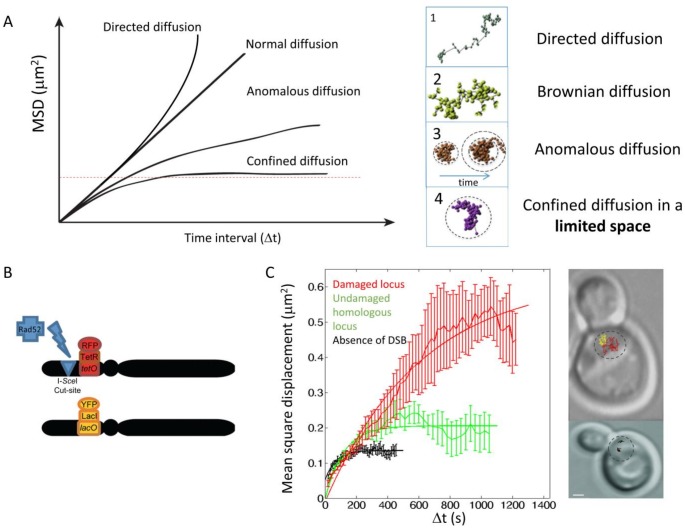
FIGURE 18: Studying chromatin mobility. **(A)** Left, theoretical Mean Square Displacement (MSD) curves for directive, Brownian, anomalous and confined diffusion. Right: corresponding typical trajectories for each mode of diffusion. **(B)** Experimental design to measure mobility at a chromosomal locus before and after DNA damage. A strain harboring two homologous and tagged loci (a *tetO/*TetR-RFP array inserted at the *URA3* locus, and a *lacO/*LacI-YFP array inserted at the homologous *URA3* locus) contains, a single I-*Sce*I cut-site located 4 kb from the *tetO* array. A galactose-inducible I-*Sce*I inserted at the *LYS2* locus allows regulated induction of a single DSB under galactose control. **(C)** Left, experimental MSD curves obtained from the diploid strain described in (B) Black: MSD curve of either tagged locus in the absence of DNA damage. Red: MSD curve of the *tetO* locus, after an I-*Sce*I-induced DSB next to the *tetO* site (local mobility). Green: MSD curve of the homologous locus harboring a *lacO* array, after an I-*Sce*I-induced DSB next to the *tetO* site (global mobility). Right: to illustrate the change in chromatin mobility, typical trajectories are shown (black trace: *URA3* locus before DNA damage; red trace: damaged *URA3* locus; yellow trace: undamaged *URA3* homologue). The MSD curves are reproduced from [369].

Once the trajectory of a locus is determined, its diffusion properties are quantified by calculating its mean-square displacement (MSD) [314]. The MSD curve represents the amount of space a locus explores in the nucleus, and its shape reveals the nature of chromatin motion (**Figure 18A**). The time-averaged MSD of a single trajectory is calculated using the following equation:

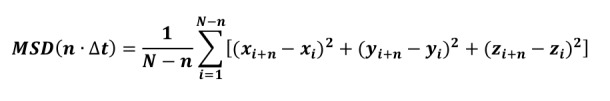
,

where N is the number of points in the trajectory, (*x, y, z*) the coordinates of the locus in 3-dimensions.

In practice, chromatin dynamics is measured in several nuclei and individual MSD curves present some variability between cells. Averaged MSD curves among several cells (time-ensemble-averaged MSD) are usually calculated and fitted to characterize the type of diffusion of a locus in a specific condition. To understand the type of motion a chromatin locus undergoes, MSD curves are fitted using the different models presented below (illustrated in Figure 18A).

##### Brownian diffusion

When a particle freely diffuses, its MSD curve is linear with time and its motion is called “Brownian”. In this case, the MSD follows:

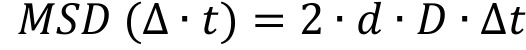


where *d* is the dimension of the movement, *D* is the diffusion coefficient of the locus, and Δ*t* is the time interval.

##### Sub-diffusive diffusion

In living cells, DNA motion is often slower than Brownian diffusion and is called “sub-diffusive diffusion” [315]. Two types of sub-diffusive diffusion have been described: confined sub-diffusion and anomalous sub-diffusion.

**Confined sub-diffusion.** When a chromosomal locus stays confined inside a sub-volume of the nucleus, its motion is called confined sub-diffusion. The MSD exhibits a plateau [313] and follows the equation:

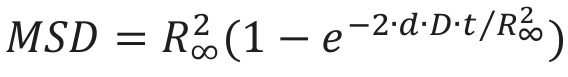


where *R*∞ is the measured plateau of the MSD, and *D* is the diffusion coefficient of the locus.

The confinement radius (*Rc*) of the motion is given by the relation: 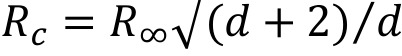 where *d* is the dimension of the motion. The MSD curve starts to bend at time 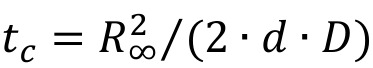 representing the characteristic, equilibration time, after which the effect of boundaries appears.

**Anomalous sub-diffusion.** When the force or structure that restricts the motion is not a simple confinement but is modulated in time and space with scaling properties, the motion is called anomalous sub-diffusion [315, 316]. In this case, sub-diffusive loci are constrained, but, unlike confined loci, they can diffuse without boundary and thus reach further targets if given enough time. For sub-diffusive motion, the MSD exhibits a power law,

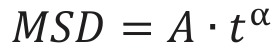


where *α*, the anomalous exponent, is smaller than 1.

The anomalous exponent α is linked to the degree of recurrence of DNA exploration, that is, the number of times a DNA locus reiteratively scans neighboring regions before reaching a distant position [317]. When α is small, the locus explores recurrently the same environment for a long time, while a large α indicates that the locus is able to explore new environments often. The anomalous diffusion coefficient A represents the amplitude of DNA motion; it is proportional to the diffusion coefficient only in the case of normal diffusion (when α = 1), which is rarely observed in biological systems [315].

#### Limitations of experimental MSD and cautionary notes

In practice, several artefacts can alter experimental MSDs. First, it is essential to take into account the effect of the inherent localization precision of the position of the spot. Localization precision can be divided into two contributions: i) the error in the determination of the accurate spot position, ii) the error due to the movement of the spot during the camera acquisition. Therefore, the finite localization precision adds a constant term to the MSD, which, if not properly accounted for, can limit or bias the exact quantification and interpretation of the MSD data. The exact analytic formula linking this constant term to the pointing accuracy and the motion blur have been calculated in the case of Brownian motion [318], and anomalous sub-diffusive motion [301].

Second, experimental MSDs are calculated from a finite trajectory length. Although each data point of the MSD is the result of an average over different times, at large time intervals, this average is performed over a smaller number of data points resulting in more scattered data and less statistical significance. To counteract this effect, the fit of MSD curves must be performed on a limited number of points to generate significant statistics. In practice, MSD curves are fitted using the first third of the MSD data points [318, 319]. To increase statistical significance, MSD fits are often performed on the time-ensemble-averaged MSD (calculated by averaging several MSDs of individual trajectories). In this case, it is important to keep in mind that fitting the time-ensemble averaged MSD prevents the detection of heterogeneities in the population and provides ensemble-averaged behavior and diffusion parameters.

Finally, the MSD approach assumes that the kind of motion the locus undergoes is the same during the time of the acquisition. When diffusion is more complex, tracking chromatin motion at different time scales uncovers the different components of the motion.

#### Multi-time scale tracking

When studying the diffusion of a specific locus, the time scale at which the data are collected reflects the behavior of the locus at that specific time scale. Thus, this experiment allows the extraction of effective diffusion parameters that are true only at that specific scale. However, chromatin presents several levels of organization, which translates into different scales in chromatin mobility. To obtain a comprehensive understanding of chromatin diffusion, several studies have investigated chromatin mobility at multiple time-scales [301, 320]. Multi-scale analysis of the trajectories reveals the superimposition of different diffusion regimes, which may comprise different natures. For example, a locus can exhibit an anomalous diffusion of *A*_micro_, in a region that itself diffuses with a coefficient *A*_macro_. Similarly, it is also possible that a locus exhibits anomalous diffusion at one time scale, but confined diffusion at a larger time scale. The MSD curves obtained at several times scales are represented altogether on log-log scale.

#### Application in the context of DNA repair:

Chromatin mobility is of particular interest in the context of DSB repair. Several studies have shown that chromatin mobility increases significantly in the presence of a DSB [321–324]. **Figure 18B** and **18C** illustrate the motion of two homologous loci in diploid yeast cells, before and after the induction of a single DSB on one locus [312]. Using multi-scale tracking following DNA damage, a recent study revealed the existence of several diffusion regimes that simultaneously drive chromatin motion [301]. In particular, chromatin exhibits increased mobility at large time scales, compared to undamaged cells, but reduced mobility at small time scales [301].

Altogether, quantifying chromatin mobility is a powerful tool to understand how chromatin explores nuclear space. Importantly, tracking and MSD analysis allows the measurement of apparent diffusion coefficients at a specific time scale. To gain a comprehensive view of chromatin diffusion, it is necessary to perform multi-scale tracking and multi-scale modeling to account for motion and rearrangements at different time scales.

### A fluorescently-tagged site-specific fork stalling assay: *LacO*-marked *RTS1*-RFB

**Figure 19:**
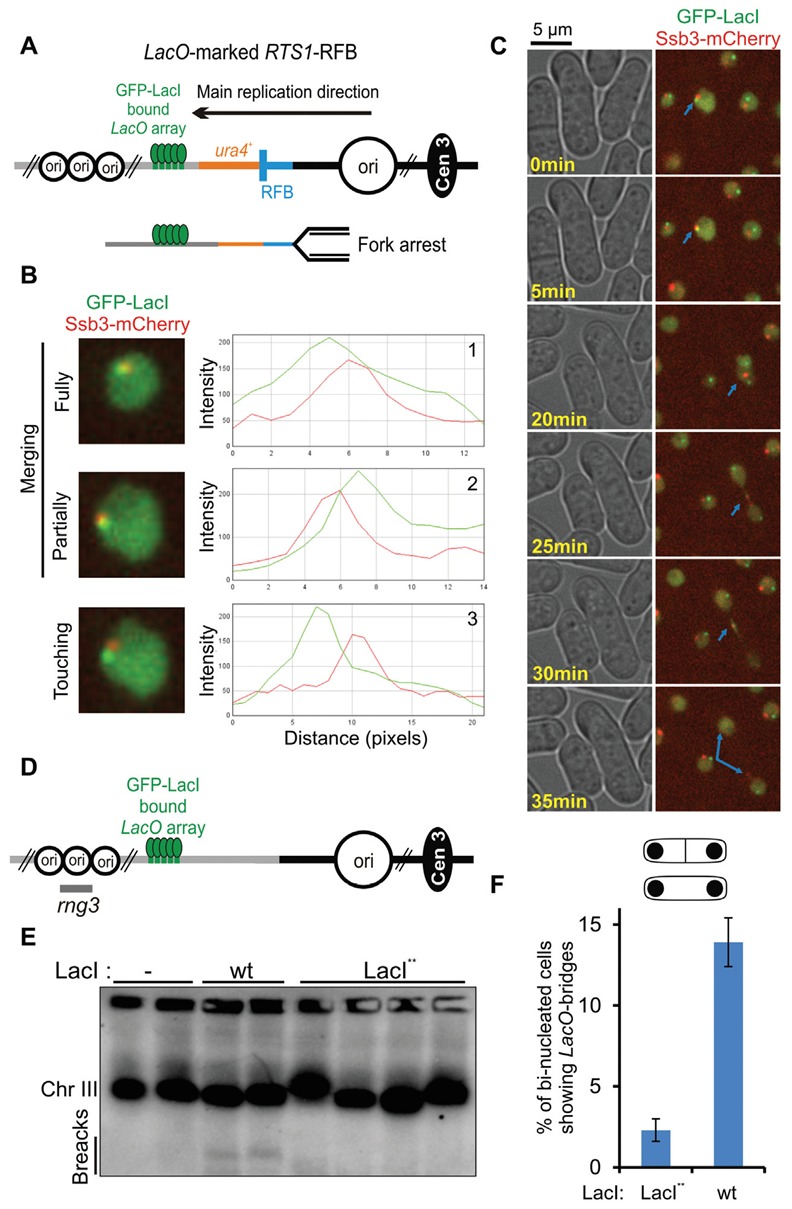
FIGURE 19: A fluorescently-marked RFB to follow *in vivo* the fate and processing of a single blocked fork in fission yeast. **(A)** Diagram of the *LacO-*marked *RTS1*-RFB locus located on the chromosome III of *S. pombe*. The blue bar indicates the *RTS1*-RFB and its polarity. Main replication origins (ori, black circles) located upstream and downstream from the *RTS1*-RFB are indicated. GFP-LacI (green ellipses) bound to *LacO* arrays (green bars) are integrated ∼7 kb away from the *RTS1*-RFB, on the telomere-proximal side of *ura4* gene (red bar). When Rtf1 is expressed, >90% of forks emanating from the strong centromere-proximal replication origin, and moving towards the telomere, are blocked. **(B)** Example of Ssb3-mCherry foci being recruited to the active RFB. Three situations were considered: GFP-LacI and Ssb3-mCherry foci were fully (1) or partially merging (2), or they were touching each other (3). **(C)** Example of an unprotected fork (identified via the presence of Ssb3-mCherry-positive signal on *LacO*-marked *RTS1*-RFB in ON condition, blue arrow) in G2 (time point 0 to 20 minutes) converted into a sister chromatid bridge during mitotic progression (from 20 to 30 minutes) and finally resolved (at 35 minutes). **(D)** Diagram of the GFP-LacI bound to *LacO* arrays located on the chromosome III of *S. pombe* without *RTS1*-RFB. The *rng3* probe is indicated. **(E)** Detection of chromosome breakage by Pulse Field Gel electrophoresis followed by Southern-blotting using the rng3 probe, in the following conditions: No LacI repressor expressed (-), expression of the wild-type (wt) LacI from a multi-copy expression vector [307], expression of the LacI^**^ from a single copy gene and SV40 promoter [333]. **(F)** Quantification of *LacO*-bridges in indicated conditions.

A fluorescence-based assay that is designed to track *in vivo* the fate of a single halted replication fork in the fission yeast *S. pombe*. Impediments to replication fork progression are a prevailing source of genome instability occurring during each cycle of cell division, contributing to the devel-opment of human diseases [325]. To address the consequences of disrupted replication forks on genome stability, site-specific fork stalling assays have been developed in yeast models and in mammalian cells [326–330]. These Replication Fork Barriers (RFBs) rely on secondary DNA structures or on the tight binding of a non-histone protein to a specific DNA sequence to act as a roadblock to fork progression. The ∼ 960 bp *RTS1* (for *Replication Termination Site 1*) sequence is a natural RFB from *S. pombe*, originally identified near the *mat* locus as an RFB necessary to fine-tune mating switching [331]. The *RTS1*-RFB was then integrated at ectopic sites, near a strong replication origin, to induce a polar roadblock to a single replisome (**Figure 19A**). Fork-arrest is mediated by the *RTS1*-bound protein Rtf1, the expression of which is regulated by the *nmt41* promoter, allowing for the *RTS1*-RFB activity to be controlled (“OFF” and “ON”). Replication forks arrested at the *RTS1*-RFB are resolved in 20 minutes via the homologous recombination pathway which protects and restarts replication forks [328, 332]. Combining the *RTS1*-RFB with fluorescently marked locus-based approaches permits to follow *in vivo* the dynamic resolution of a single blocked fork in space and time [333]. Around 120 interrupted *LacO* repeats were introduced ∼7 Kb away of the *RTS1*-RFB to create the *LacO-*marked *RTS1*-RFB locus (Figure 19A). *LacO* repeats are detected *in vivo* through the binding of the repressor LacI whose N- and C-terminals are fused to a fluorescent epitope (GFP or mCherry) and to a NLS, respectively. Snapshot and time-lapse fluorescently-based microscopy can be employed to visualize LacI foci which mark the site of fork arrest in living cells using samples prepared in agarose pads or cells injected into dedicated 4-5 μm-thick poly-dimethyl-siloxane (PDMS) microfluidic chambers on glass coverslips. Detailed protocols for yeast cell imaging and sample preparations have been described [333, 334].

A first utility of the *LacO*-marked *RTS1*-RFB is to investigate the dynamic mobility of a single arrested replisome, respectively to surrounding nuclear compartments, by monitoring the Mean Square Displacement (MSD) of LacI foci, as reported for other fluorescently-marked site-specific DNA damage assays [298]. A second approach is the analysis of the recruitment of DNA repair factors tagged with a fluorescent epitope (taking care not to overlap their emission spectra with that of the tagged LacI), to decipher the mechanisms of fork processing. For example, the fusion protein Ssb3-mCherry, one subunit of the trimeric single stranded DNA binding factor RPA (for *Replication Protein* A) was used to analyze the formation and repair of single stranded DNA (ssDNA) at the *RTS1*-RFB during cell cycle progression (**Figure 19B**). A limited amount of S-phase cells showed RPA recruitment to LacI foci, revealing the highly transient nature of ssDNA which is generated by the concerted action of several nucleases and then quickly resolved as the arrested fork is successfully restarted or rescued by an opposite fork [333]. In specific genetic backgrounds (in the absence of the recombinase Rad51 or its loader Rad52), numerous S-phase and G2 cells showed an extensive RPA recruitment to LacI foci. These observations indicate that the *RTS1*-RFB accumulated large stretches of ssDNA (*i.e.* defined as unprotected forks) which are left unrepaired when cells enter mitosis, revealing that the arrested fork is neither restarted nor rescued by an opposite fork. Unprotected forks are then converted into RPA-positive intertwined sister chromatids resembling unconventional mitotic bridges (*i.e.* refractory to classical DNA dyes), containing both ssDNA (marked by Ssb3-mcherry) and double stranded DNA (dsDNA) (labelled by GFP-LacI which binds only double stranded *LacO* sequences) (Figure 19C). These approaches have revealed that unconventional mitotic bridges resembling human ultra-fine bridges are more complex than anticipated. In addition, the *LacO*-marked *RTS1*-RFB has proved to be sensitive enough to monitor the amount of RPA recruited at the arrested fork by quantifying the fluorescence intensity of Ssb3-mcherry being recruited to LacI foci, after normalization by the nucleus area and background fluorescence intensity [335].

#### Cautions

Beyond already described issues when using *LacO*-arrays bound by LacI as a fluorescently genomic tag, specific cautions are raised when combining with a site-specific RFB. The tight binding of LacI to *LacO* arrays acts as an intrinsic and non-polar RFB, resulting in mitotic bridges, chromosomes breakage and gene silencing [307–309]. Two approaches help to overcome this issue. First, a LacI variant (called LacI**) was reported to alleviate the impact of *LacO*-bound LacI to fork progression while allowing LacI foci to be detected by cell imaging [309]. Second, over-expression of LacI should be avoided to limit nucleoplasmic LacI signal which might be confused with DNA bridges [307]. In fission yeast, the use of LacI** expressed as a single gene copy from SV40 promoter allowed to avoid chromosome breakage and mitotic *LacO*-bound LacI bridges (**Figure 19D-F**). In all cases, appropriate controls should be employed (*i.e. LacO*-bound LacI without an active RFB) to demonstrate that cellular transactions occurring at LacI foci reflect the activity of the RFB.

The location and distances of *LacO* arrays relatively to the inducible RFB are one limitation of the system. If possible, both *LacO* arrays and the RFB should be replicated by two sister replication forks [330]. Alternatively, *LacO* arrays can be placed downstream the RFB in a way that the replication fork encounters first the active RFB. The duplication of the *LacO* arrays, revealed by two adjacent sister LacI foci can be used as a readout of the replication of genomic sequences located downstream the RFB, either by the restarted fork or by the opposite fork [333]. In this situation, distances between *LacO* arrays and the RFB should be taken into consideration. Restarted replication forks are often associated with error-prone DNA synthesis whose mutation rate decreases as the restarted fork progresses [336]. Such erroneous DNA synthesis might make the repetitive nature of *LacO*-arrays unstable and difficult to maintain over several cell divisions. Increasing the distance between the *LacO*-arrays and the RFB may render difficult to conclude whether or not a specific DNA repair factor is recruited to the RFB. For example, RPA was reportedly being recruited to the active RFB when Ssb3-mCherry foci were either touching or fully or partially merging with LacI foci (**Figure 19C**). Finally, *LacO* arrays can be located upstream the RFB. However, the processing of replication forks includes the resection of newly replicated strands, up to 3 Kb in some specific genetics backgrounds, resulting in single stranded *LacO* arrays which will not be bound by LacI.

### A microscopy-based assay to measure DNA double-strand break end resection in single fission yeast cells

The decision of whether to repair a DNA DSB through canonical (NHEJ or HDR is orchestrated by control over DSB end resection. End resection, or the programmed 3′ to 5′ nucleolytic degradation at the DSB that generates the ssDNA necessary for homology-dependent pairing, commits a DSB to HDR [202, 337, 338]. Thus, repression of resection initiation is essential to promote canonical NHEJ, the preferred repair mechanism in cells prior to DNA replication [337–339]. Numerous factors, such as the Ku heterodimer, the Mre11-Rad50-Nbs1/Xrs2 (MRN/MRX) complex and Sae2/Ctp1/CtIP regulate the initiation of resection (<100 bp) [338, 340–344]. Downstream of initiation, long-range resection (∼100s bps) can be catalyzed by two machineries: the exonuclease Exo1 and the combination of a RecQ helicase (e.g. Sgs1/Rqh1/BLM) and Dna2 [339, 345–348].

How cells determine and execute the preferential engagement of one of these long-range resection pathways is poorly understood. While Exo1- and Sgs1-depenent end resection appear redundant in budding yeast [338, 348], in fission yeast wild type cells utilize specifically the Exo1 pathway [349, 350]. Our work suggests that one consequence of up-regulation of the RecQ helicase-mediated end resection pathway is more rapid (and therefore likely more extensive) resection [350]. However, the mechanisms and consequences of resection mechanism choice remain poorly understood. Further, as chromatin remodeling has been linked to long-range resection efficiency [345], likely many additional factors quantitatively impinge on resection rate, which will require further study. Lastly, as the generation of ssDNA by resection may be uncoupled from loading of repair factors depending on the nuclear context (for example, with heterochromatin [324]), approaches that can monitor DSB end resection within the nuclear context will be enabling technologies of further discovery. Numerous approaches to measure end resection in populations of cells have been developed, such as Southern blotting or quantitative PCR to detect ssDNA generation or protection from restriction enzyme digestion. In intact individual cells, DSB end resection is often inferred by monitoring the loading of ssDNA binding proteins such as RPA or Rad51.

To facilitate the dynamic measurement of resection within the context of the nucleus, we recently developed a live-cell assay capable of revealing long-range resection rates in individual fission yeast cells [350]. This assay is based on signal loss of LacI-GFP from a LacO array adjacent to a site-specific, inducible DSB. This technology allows the position of a DSB to be interrogated simultaneous with quantitative measures of resection rate with single cell resolution.

#### Description of the assay

**Figure 20:**
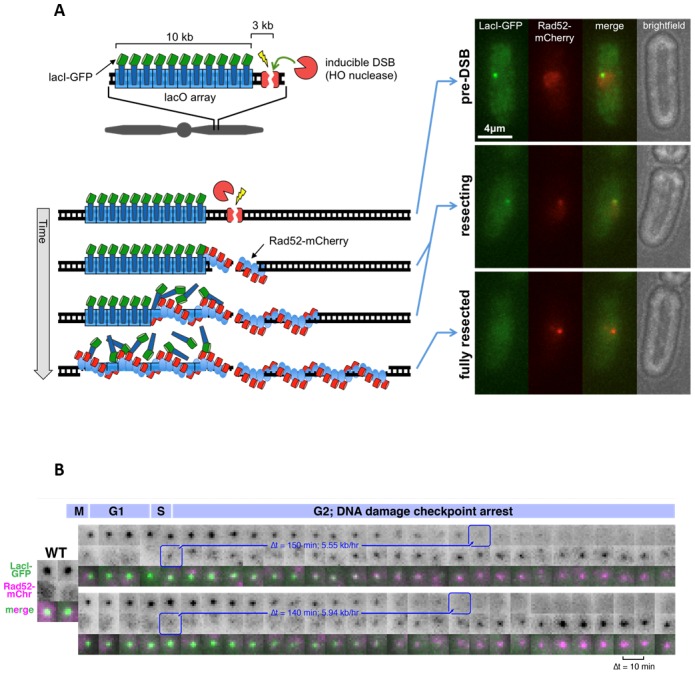
FIGURE 20: Microscopy-based assay for quantitative measurement of DSB end resection rates in single cells. **(A)** Cartoon diagram of the assay principles, including a recognition site for the HO nuclease adjacent to a LacO array. Resection drives loss of LacI-GFP and binding of Rad52-mCherry. On the right are examples of images that highlight the phases of DSB induction and processing. **(B)** Time-lapse movie of two sister cells that each form a DSB in S phase and undergo resection. The first frame when Rad52-mCherry is detected at the DSB and last frame in which the LacI-GFP focus is detected are boxed in blue. This gives rise to the resection duration, from which the resection rate can be calculated (blue). The time intervals are 10 minutes.

The principle of the live-cell DSB end resection assay is that the conversion of dsDNA to ssDNA across a LacO array can be monitored by the loss of LacI-GFP binding. By engineering the LacO array proximal to a site-specific DSB (in this case, a recognition site for the HO endonuclease from budding yeast, which is absent in fission yeast), processive resection can be monitored (**Figure 20A**). As the induction of the DSB is neither synchronous nor occurs in all cells, we identify cells that have initiated end resection by the loading of Rad52-mCherry, which colocalizes with the LacI-GFP/LacO focus (**Figure 20A**). While this assay provides indirect information about the kinetics of the initiation of DSB end resection following induction of the HO nuclease by proxy of the loading of Rad52-mCherry, validation of slow initiation of end resection requires orthogonal approaches to ensure that the efficiency of DSB induction and the loading of Rad52 are unaffected. Thus, this assay is best suited to monitor the kinetics of long-range resection after initiation. Given that we know both that <300 bps of resection are required to visualized Rad52-mCherry onto the resulting ssDNA at the DSB [350, 351] and the distance (in bps) between the HO cut site and the start and the end of the LacO array, we can convert the time it takes to resect the full LacO array to a long-range resection rate (**Figure 20B**). For WT fission yeast cells, which rely on Exo1-mediated long-range resection, the median rate is 7.6 kb/hr [350].

Further insights into long-range resection mechanism can be obtained by employing genetic perturbations. For example, deletion of the fission yeast RecQ helicase, Rqh1 (orthologous to budding yeast Sgs1 and human BLM), allows Exo1-dependent resection to be studied [350]. While loss of Exo1 disrupts long-range resection in otherwise wild type fission yeast [349], deletion of Crb2 (orthologous to budding yeast Rad9 [352] or human 53BP1 [353–355]) derepresses Rqh1-dependent resection, allowing this mechanism to be explicitly studied [350].

Lastly, because this assay is microscopy-based, it enables the position and dynamics of the DSB to be interrogated simultaneously with end resection. We note that resection from the DSB through the full LacO array (∼13 kb), which takes on average just under 2 hours, appears insufficient to drive targeting to the nuclear periphery. Thus this relocalization of a persistent DSB, described by others in diverse eukaryotes [324, 356–361] and by us in fission yeast [362], appears to require very extensive resection and/or DSB persistence. However, many critical questions into how nuclear subcompartments influence DSB repair mechanism choice and efficiency remain. Future adaptations of this assay will include employing mechanisms to test how tethering the DSB to different nuclear structures and altering the local chromatin state impacts on DSB processing.

#### Cautionary Notes

##### Obtaining reproducible, HO-driven DSB induction in fission yeast

One major challenge is to maintain a strain containing the site-specific nuclease cut site in the presence of the integrated, inducible cognate nuclease, as even transient leakiness of the nuclease promoter leading to nuclease expression can lead to cells with insertion-deletions in the cut site; these cells can then take over the cell population and disrupt the efficiency of inducible DSBs within the observation window. To overcome this, we use the Cre-driven HO nuclease integration system developed by Tony Carr's laboratory [363], which allows for induction in the presence of uracil. Importantly, we obtain the best results when we transduce the Cre/HO nuclease plasmid just prior to carrying out the assay and induce Cre expression overnight immediately prior to DSB induction by addition of uracil, thereby taking advantage of the high efficiency of the Cre recombinase, which mitigates the need for selection prior to DSB induction. We have also observed that transformants selected for uptake of the Cre donor plasmid demonstrate the most reproducible HO-induced DSB induction if the cells are used between five and ten days after transformation.

##### Photobleaching

As this assay interprets the loss of LacI-GFP intensity, it is essential that the user both mitigates (during the experiment) and assesses (during the analysis) the photostability of the LacI-GFP signal. Photobleaching is reduced by the addition of the oxygen scavenger, *n*-Propyl gallate (NPG; 0.1 mM), to both the growth media prior to imaging and the agarose pad on which the cells are maintained and imaged. We suggest initially dissolving the NPG in ethanol at 10 mM followed by a 1:100 dilution in water. Prior to analysis, each field is assessed for photostability over the 5 h image acquisition period by monitoring the GFP intensity of control cells at the end of the movie that did not sustain the targeted DSB (that is, lacking a Rad52-mCherry focus). Only fields that maintain robust LacI-GFP intensity in these control cells are further analyzed.

##### Validation of DSB induction and resection at the population level by qPCR

Our previous characterization demonstrates that the induction efficiency of the site-specific DSB is largely unaffected by mutations that compromise the resection machinery [350]. Moreover, we find that the vast majority of site-specific DSBs commit to homology-directed repair by initiating resection in G2 fission yeast, which can be assessed by Rad52-mCherry loading at the LacO array [350]. However, confirming the efficiency of DSB induction and the influence on resection at the population level is still recommended. To achieve this, we routinely employ quantitative real-time PCR (qPCR). To determine the DSB induction frequency, PCR primers across the DSB site can be employed; here loss of this product compared to a control PCR product can be quantified [350]. It should be noted, however, that since this is a signal-loss experiment, the PCR product across the cut site typically decreases by only ∼15-20%. An orthogonal method to investigate resection at the population level is to monitor protection from restriction enzyme digestion by qPCR [204, 349] using the same assay strains and conditions used for microscopy. Importantly, in this case the efficiency of resection can only be timed relative to the induction of the HO nuclease (addition of uracil to the media), and thus includes the delay for HO nuclease production, individual cells to reach S phase (when the HO nuclease has access to cut its site-specific recognition site), and the process of resection itself. Moreover, if there is a delay in resection initiation this will dominate the observations, making it impossible to independently measure the long-range resection rate. In the microscopy-based assay we isolate the long-range resection phase, which can be observed even if relatively fewer cells initiate timely resection (that is, load Rad52-mCherry at the LacO array).

##### An irreparable DSB

One additional caveat is that resection monitored using this assay is extensive (over 10 kb) driven by the fact that this is an irreparable DSB. We have evidence that in the vast majority of cells both sister chromatids are cut by the HO nuclease (data not shown). When the assay is carried out in haploid cells (as described), this renders this a lethal DSB that is subjected to prolonged resection.

#### Conclusion

Microscopy-based approaches that enable real-time visualization of distinct events in DSB processing and repair hold great promise for providing quantitative, dynamic, single-cell information previously inaccessible with genetic marker assays. Combined with assays of repair outcome, we are at the cusp of realizing new insights into the mechanisms that underlie the observed inhomogeneity across the genome in the susceptibility to DNA damage and/or the outcome of DNA repair [45, 364]. Combining the power of yeast genetics with existing and future live-cell DSB repair assays will continue to provide powerful insights into the cell biology of DNA repair.

## SUMMARY

Here we have presented an overview of three broad areas of assays for mutagenesis and recombination in bacterial and fungal systems, focusing on genetic, molecular and cytological approaches. Each singular assay is described with its utility and caveats, and has been shown to advance the field and our knowledge regarding the mechanisms and central protein components of the multiple DNA repair and recombination pathways. The synergistic application of multiple assays that includes assays from each broad category has made the bacterial and fungal systems the foundation for recombination and repair studies.

## SUPPLEMENTAL MATERIAL

Click here for supplemental data file.

All supplemental data for this article are available online at http://www.microbialcell.com/researcharticles/2019a-klein-microbial-cell/.

## Abbreviations:

5-FOA: 5-fluoro-orotic acid,
ahTET: anhydrotetracycline,
BER: base excision repair,
BIR: break-induced replication,
CGR: complex genome rearrangement,
ChIP-seq: chromatin immunoprecipitation followed by sequencing,
DSBs: double-strand break,
DSBR: double-strand break repair,
FLIP: fluorescence loss in photobleaching,
FRAP: fluorescence redistribution after photobleaching,
GamFP: Gam-fluorescent protein,
GCR: gross chromosome rearrangement,
HJs: Holliday junctions,
HR: homologous recombination,
iFRAP: inter-foci Fluorescence Redistribution After Photobleaching,
LOH: loss of heterozygosity,
MMEJ: micro-homology mediated end joining,
MMS: methyl methanesulfonate,
MSD: mean-square displacement,
NER: nucleotide excision repair,
NHEJ: nonhomologous end joining,
PAFPs: photoactivable fluorescent proteins,
PFGE: pulsed field gel electrophoresis,
RDG: RuvCDefGFP,
RFBs: replication fork barriers,
RTS1: replication termination site 1,
SCE: sister-chromatid exchange,
SCR: sister-chromatid recombination,
SDSA: synthesisdependent strand annealing,
SPT: single-particle tracking,
sptPALM: single-particle tracking combined with Photoactivated Localization Microscopy,
SSA: single strand annealing,
UV: ultraviolet,
YAC: yeast artificial chromosome.
