# Supplementary material for: Guidelines for DNA recombination and repair studies: Cellular assays of DNA repair pathways
Source: Microb Cell. 2019 Jan 7;6(1):1–64. doi: 10.15698/mic2019.01.664 (PMC6334234; doi:10.15698/mic2019.01.664)
Supplement: Supplementary file 1 [file mic-06-001-s01.pdf]

Title	Authors	Contact information of lead authors
1.1 Forward mutation assays	Nayun Kim	nayun.kim@uth.tmc.edu
1.2, 1.3 Reversion mutation assays	Nayun Kim, Sue Jinks-Robertson	sue.robertson@duke.edu
2.1 Sister chromatid recombination	Belén Gómez-González, Andrés Aguilera	andres.aguilera@cabimer.es
2.2 Direct repeat assays	Anastasiya Epshtein, Hannah Klein	hannah.klein@nyumc.org
2.3, 2.4 Ade2 assays for mitotic recombination in haploids and diploids	Léa Marie, Lorraine Symington	lss5@cumc.columbia.edu
2.5 Detection and analysis of mitotic recombination events	Yi Yin, Thomas Petes	tom.petes@duke.edu
3. Gross chromosomal rearrangements	Christopher Putnam, Richard Kolodner	rkolodner@ucsd.edu
4.1 Repeat expansions and repeat-mediated genome instability	Elina Radchenko, Sergei Mirkin	sergei.mirkin@tufts.edu
4.2 Yeast artificial chromosomes and fragility of DNA sequences	Erica Polleys, Catherine Freudenreich	Catherine.Freudenreich@tufts.edu
5. Chromosome rearrangements associated with gene amplification	Juan Lucas Argueso	lucas.argueso@colostate.edu
6. Detection and quantification of DSBs by pulsed field gel electrophoresis	Sharik R. Khan, Andrei Kuzminov	kuzminov@life.illinois.edu
7.1 Induction of site-specific DSBs and analysis of their repair in budding yeast	James E. Haber	haber@brandeis.edu
7.2 DNA double-strand break induction and repair in fission yeast	Sandeep Kumar, Grzegorz Ira	gira@bcm.edu
7.3 Quantification of strand-specific single-stranded DNA in DNA repair by real time PCR	Jun Che<br>Sang Eun Lee	lees4@uthscsa.edu
7.4 Detection of toxic recombination	Rajula Elango,<br>Anna Malkova	anna-malkova@uiowa.edu

intermediates in yeast *Saccharomyces cerevisiae*		
8. Detection of hypermutable single-strand DNA formed in living yeast cells	Cynthia J. Sakofsky, Dmitry A. Gordenin	gordenin@niehs.nih.gov
9.1 Engineered proteins for detection of DNA double-strand breaks in living bacterial and mammalian cells	Kyle M. Miller, Devon M Fitzgerald, Susan M. Rosenberg	Kyle.miller@austin.utexas.edu<br>smr@bcm.edu
9.2 Engineered proteins for detection of Holliday junctions in living bacterial cells	Jun Xia, Qian Mei, Susan M. Rosenberg	smr@bcm.edu
10.1 Single-particle tracking of nucleotide excision repair proteins inside living bacteria	Alicja Piotrowska, Mathew Stracy, Pawel Zawadzki	zawadzki@amu.edu.pl
10.2 Inter-foci fluorescence redistribution after photobleaching (iFRAP) to measure dynamics of DNA repair centers	Giedrė Bačinskaja, Michael Lisby	mlisby@bio.ku.dk
11.1 Visualizing chromosomal dynamics upon DNA double-strand breaks in yeast *S. cerevisiae*	Anais Cheblal, Susan M Gasser	susan.gasser@fmi.ch
11.2 Quantifying the mobility of a chromosomal locus	Judith Miné-Hattab, Rodney Rothstein	rjr4@cumc.columbia.edu
12. A fluorescently-tagged site-specific fork stalling assay	Anissia Ait Saada, Sarah AE Lambert	sarah.lambert@curie.fr
13. A microscopy-based assay to measure DNA double-strand break end resection in single fission yeast cells	Bryan A. Leland, Megan C. King	megan.king@yale.edu
